# Fine scale population structure of *Acropora palmata* and *Acropora cervicornis* in the Colombian Caribbean

**DOI:** 10.7717/peerj.13854

**Published:** 2022-08-30

**Authors:** Rocio García-Urueña, Sheila A. Kitchen, Nikolaos V. Schizas

**Affiliations:** 1 Facultad de Ciencias Básicas, Universidad del Magdalena, Santa Marta, Magdalena, Colombia; 2 Division of Biology and Biological Engineering, California Institute of Technology, Pasadena, CA, United States of America; 3 Department of Marine Sciences, University of Puerto Rico, Mayagüez, PR, United States of America

**Keywords:** Genetic connectivity, Conservation status, Scleractinian corals

## Abstract

Using a standardized SNP array, we identified two populations of *Acropora cervicornis* and one population of *A. palmata* in the Caribbean coast of Colombia. San Andrés was the most genetically differentiated location for both species. An average pairwise F_ST_ value of 0.131 and 0.050 between San Andrés and neighboring collection sites was estimated, for *A. cervicornis* and *A. palmata,* respectively. Based on population patterns of both acroporid species, we inferred that Magdalena River is not a barrier of genetic connectivity among Colombian populations. Genetic comparisons between the Colombian coast of Caribbean with other Caribbean locations agree with previous studies for both species*,* where four populations were identified in *A. cervicornis* and three in *A. palmata.* Our results support published bio-physical model predictions and highlight the Panama-Colombia gyre as a possible isolating mechanism within the western Caribbean. However, the genetic diversity in both species was about half (mean HE per site = 0.321 in *A. palmata* and 0.369 in *A. cervicornis*) than previous estimates in acroporid populations in the Caribbean. The lower genetic diversity as well their relative isolation and high levels of reef degradation may be of particular conservation concern that may require species-specific management coupled with science-based restoration efforts.

## Introduction

The two species of *Acropora* are found throughout the entire Colombian Caribbean; however, they present different states of conservation. While there are still important patches of *Acropora palmata*, *A. cervicornis* patches are rare and fragmented, and in some locations they are practically absent (García-Urueña & Garzón-Machado, 2020). The deterioration and reduction of populations has been documented since the 1990s and was attributed to increased sedimentation, reduced light, increased nutrients, diseases, and the use of dynamite (Garzón-Ferreira & Cano, 1991; Díaz et al., 2000). These factors add to what have been identified as stress factors throughout the Caribbean such as coastal development, climate change, overfishing, tourism practices (Hoegh-Guldberg, 1999; Buddemeier, Kleypas & Aronson, 2004), specific acroporid diseases such as white band and white pox (WPX) (Porter et al., 2001; Patterson et al., 2002) and bleaching (Muller et al., 2008; Rogers & Muller, 2012). Consequently, the two species have been listed as threatened under the US 1973 US Endangered Species Act (ESA), ever since 2006 (Aronson et al., 2008a; Aronson et al., 2008b). In Colombia, these two species are classified in the red book as endangered and critically endangered (Ardila, Navas & Reyes, 2002).

The population genetic structure of both *Acropora* species has been extensively studied in the Caribbean. *Acropora palmata* stands are structured into two long-separated populations, Eastern and Western, with the northern genetic break being located around the Eastern Puerto Rican region (Baums, Miller & Hellberg, 2005; Baums, Miller & Hellberg, 2006; Mège et al., 2014; Devlin-Durante & Baums, 2017) and the southern being located somewhere between Panama and the Netherlands Antilles (Baums, Miller & Hellberg, 2005). Regional subpopulations have also been documented. According to Porto-Hannes et al. (2015), *A. palmata* populations were grouped into four sub-regions from Mesoamerican Barrier Reef System, Panamá, Puerto Rico and Venezuela, and Kitchen et al. (2020) identified three populations consistent with Devlin-Durante & Baums (2017) recovering the East/West divide with additional substructure between Puerto Rico and Curaçao in the East. The population structure of *A. cervicornis* has additional subdivisions at regional and local scales throughout the Caribbean, with limited larval dispersal over moderate to long distances (>500 km) (Vollmer & Palumbi, 2007; Hemond & Vollmer, 2010; Baums et al., 2010; Drury et al., 2017). At least three populations have been identified with substructure detected between the Western Caribbean populations of Florida and Belize (Kitchen et al., 2020).

Regional circulation patterns are important to understand the genetic connectivity of populations, in this regard along the Central and South American coast of the Caribbean, Andrade, Barton & Mooers (2003) strongly suggests the existence of an eastward flow from Panama to the Antilles, counter to the Caribbean Current. However, numerical simulations suggest that this flow is a semi-continuous feature along the entire southern boundary of the Caribbean and is associated with offshore cyclonic eddies. In addition, the Panama-Colombia Countercurrent is stronger (6 Sv) off the Panamanian coast, but most of its transport is recirculated in the Southwest Caribbean Gyre rather than continuing along the Colombian coast, with a portion (1 Sv) of the flow continued eastward along the coast of Colombia and Venezuela (Andrade, Barton & Mooers, 2003). Galindo, Olson & Palumbi (2006) developed a genetic model that used connectivity estimates from oceanographic models to predict genetic patterns resulting from larval dispersal in a Caribbean coral and the results indicated similar geographic groupings of genetically clustered populations. These groupings included Colombia with the Panamá cluster in the southwestern Caribbean. Although Porto-Hannes et al. (2015) mentioned that populations from Panama and Venezuela may be the result of geographic distance combined with the circular gyre of the Caribbean current in the Colombian basin, likely preventing larval dispersal from Venezuela to Panama, and argued that this result may be a barrier formed by a plume of low salinity runoff from the Magdalena River (Colombia) as was studied by Restrepo & Kjerfve (2000).

Species of the reef-building coral genus *Orbicella* have also been studied throughout the Caribbean, and in general the same genetic separation of populations has been observed between the East and West, with a genetic break around the Mona Passage in the North, and in the southern extent they observed a significant level of gene flow between Curaçao and Mexico (Rippe et al., 2017). Foster et al. (2012) integrated a spatially realistic Lagrangian model of larval dispersal and a theoretical genetic model for *Orbicella annularis* including samples for the Colombian Caribbean. They observed a genetically differentiated species, with three groups of populations: an Eastern cluster (Lesser Antilles, Venezuela and Curaçao), a Western cluster (the Bahamas Archipelago, Cuba, Belize and Cayman Islands) and the central group that identifies the Colombian population with Honduras, Nicaragua, Jamaica, Dominican Republic, Puerto Rico and British Virgin Islands.

Estimating the genetic diversity and connectivity of the populations of *A. palmata* and *A. cervicornis* in the Colombian Caribbean is necessary to know to what extent the populations of these species in the Southern Caribbean are connected and their importance as a source to contribute to the management and conservation programs. For these reasons we used a Single Nucleotide Polymorphism (SNP) array and standardized analysis work flow for the genus *Acropora* recently developed by Kitchen et al. (2020) to (1) to estimate genetic diversity of *A. palmata* and *A. cervicornis* populations and compare them to those of previously studied Caribbean populations, (2) identify patterns of genetic connectivity of the acroporid subpopulations in the Colombian Caribbean that can provide information for their management and conservation and (3) to analyze different factors explaining the genetic differences among *A. palmata* and *A. cervicornis* subpopulations in this region.

## Material & Methods

### Sample collection

**Figure 1:**
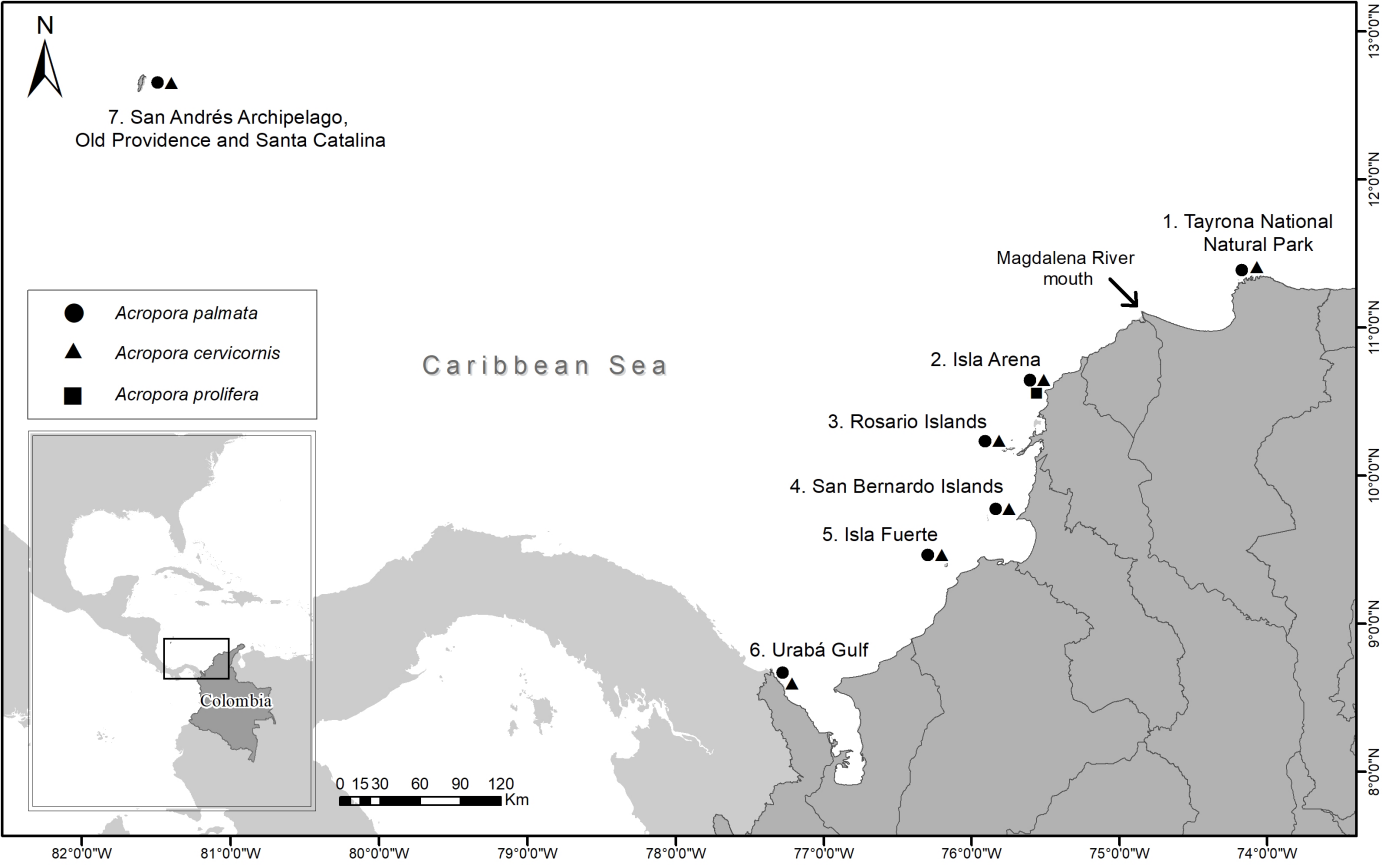
Sampling locations of *Acropora palmata* and *A. cervicornis* colonies along the Colombian Caribbean.

Samples were collected between 2016 and 2018 from 35 reef sites for seven locations (Tayrona National Natural Park (TNNP), Isla Arena, Rosario Islands, San Bernardo Islands, Isla Fuerte, Urabá Gulf and San Andrés Archipelago, Old Providence and Santa Catalina) along the Colombian Caribbean (Fig. 1). A total of 97 *A. palmata,* 86 *A. cervicornis* and seven *A. prolifera* colonies were biopsied. At each site, colonies were sampled at least 5 m apart and at depths between 1 and 10 m (Table 1) (National Natural Parks Unit of Colombia, permit 001, March 6, 2017). From each colony, ca. 1 cm^2^ of live tissue was taken and preserved in 95% ethanol.

### SNP-based genotyping and taxonomic classification

Total genomic DNA was extracted using a DNeasy Tissue Extraction kit (Qiagen) and quality was quantified using a Nanodrop 2000 and an Agilent 2100 Bioanalyzer. Genomic DNA was submitted to Thermo Fisher for genotyping analysis on the Axiom™ Coral-Algae Genotyping Array (Thermo Fisher, Waltham, MA, USA). The raw genotype data was processed using the Standard Tools for *Acropora* Genotyping analysis portal (https://coralsnp.science.psu.edu/galaxy, Kitchen et al., 2020). In brief, each sample was genotyped at 19,694 SNP loci and compared to a database of previously genotyped samples to calculate a genetic distance matrix. Samples were assigned a multilocus genotype (MLG) based on an absolute genetic distance threshold of 0.032 to samples within this study and prior samples (Table S1). To determine the genetic taxonomic identification, the percentage of observed heterozygosity and homozygosity of a set of species-specific markers was calculated as previously described (Kitchen et al., 2020).

### Analyses

The two Caribbean species were separated for the remaining analyses. For each species, a representative sample for each MLG was extracted and combined with representative samples spanning the geographic range of the Caribbean acroporids (Table S1, (Kitchen et al., 2020)). An additional filter of 5% minor allele frequency was applied to the SNP loci of each species separately using VCF tools (Danecek et al., 2011) resulting in 6,201 SNPs from the 121 *A. cervicornis* genets and 7,078 SNPs from the 159 *A. palmata* genets.

**Table 1:** Sampling locations and estimates of genetic diversity of *Acropora cervicornis* and *A. palmata* obtained from reefs along the Colombian Caribbean.

Species	Locality	Reef	Latitude	Longitude	Nc	N	Ng	He	Ho	Fis	*π*
*A. cervicornis*	TNNP	Nenguange	11,32649	−74,07825	3	0	0	0,3083	0,3067	0,0054	0.364 ± 0.011
		Chengue	11,32130	−74,12826	4	3	3				
		Cinto	11,33625	−74,05276	3	2	1				
	Isla Arena	Isla Arena	10,73864	−75,3498	3	3	1	NA	NA	NA	NA
	Rosario Islands	Isla Fiesta	10,18555	−75,73805	1	1	1	0,3165	0,3243	−0,0246	0.367 ± 0.011
		Isla Grande	10,18444	−75,73194	2	2	2				
		Caribarú	10,17194	−75,75527	4	3	3				
		Luis Guerra	10,16962	−75,75046	5	5	5				
		Pavitos	10,17306	−75,76778	3	3	2				
	Cartagena	Cartagena	10,24831	−75,62425	1	0	0	NA	NA	NA	NA
	San Bernardo Islands	Ceicen	9,70666	−75,85111	4	3	1	0,3167	0,3212	−0,0144	0.372 ± 0.032
		Batea	9,80222	−75,8194	2	2	2				
		Bajo Hojuela	9,81472	−75,85583	4	4	2				
		De los Santos	9,75014	−75,86858	5	5	3				
		La Pared	9,80244	−75,81946	5	5	4				
	Isla Fuerte	Isla Fuerte	9,36818	−76,20421	2	0	0	NA	NA	NA	NA
	Urabá Gulf	Bajo Naui	8,64472	−77,33972	4	3	3	0,3405	0,3333^*^	0,021	0.364 ± 0.011
		Aguacate	8,62193	−77,32524	3	3	1				
		Cabo Tiburón	8,67194	−77,35722	3	3	3				
	San Andrés Islands	Roncador	13,50027	−80,03194	2	2	2	0,3257	0,3236	0,0064	0.393 ± 0.012
		Quita Sueño	14,24861	−81,23861	4	4	4				
		Providencia	13,37777	−81,38666	3	3	3				
		West Point	12,59528	−81,71111	3	3	3				
		Cayo Bolívar	12,43034	−81,48384	1	1	1				
		Plaza de Toros	12,59528	−81,71111	4	3	3				
		Serrana	14,36250	−80,16138	4	4	4				
TOTAL					82	70	57				
*A. palmata*	TNNP	Isla Aguja	11,32000	−74,20083	5	5	5	0,3618	0,362	−0,001	0.364 ± 0.012
		Concha	11,32222	−74,16666	5	5	4				
		Chengue	11,31749	−74,13393	5	4	3				
		Gayraca	11,32319	−74,11334	4	4	4				
		Nenguange	11,32097	−74,07801	3	0	0				
		Cinto	11,33172	−74,05947	5	1	1				
		Aguja	11,31080	−74,19032	5	5	4				
	Isla Arena	Isla Arena	11,23472	−75,60111	4	4	4	0,3627	0,3608	0,005	0.361 ± 0.014
	Rosario Islands	Isla Fiesta	10,18553	−75,72790	4	4	3	0,3693	0,3593	0,027	0.367 ± 0.011
		Isla Rosario	10,16262	−75,79881	2	2	2				
	Cartagena	Punta Brava	10,18500	−75,74556	3	3	3	0,3654	0,3771	−0,032	0.372 ± 0.006
	San Bernardo Islands	Maravilla	9,76130	−75,87303	8	6	5	0,3659	0,3935	−0,755	0.372 ± 0.032
		Bajo Hojuela	9,81472	−75,85583	10	10	3				
		Ceicen	9,70666	−75,85111	5	5	1				
		Batea	9,80444	−75,82111	1	1	1				
		Llantas	9,80808	−75,83299	1	1	1				
	Urabá Gulf	Bajo Naui	8,64472	−77,33972	13	13	13	0,3617	0,3612	0,0014	0.365 ± 0.011
		Cabo Tiburón	8,67111	−77,35805	4	4	4				
		Coquera	8,65278	−77,34556	3	3	3				
	San Andrés Islands	Serrana	14,36250	−80,16138	4	3	3	0,3942	0,3881^*^	0,0155	0.393 ± 0.012
		Roncador	13,56500	−80,04055	3	2	2				
					97	85	69				
*A. prolifera*	Isla Arena	Isla Arena	11,23472	−75,60111	5	1	1	NA	NA	NA	NA
	Roncador		13,50027	−80,03194	1	1	1				
	Cabo Tiburon^*^		8,67194	−77,35722	1	1	1				
					7	3	3				

**Notes.**

Nc total number of samples collected N total number of samples successfully genotyped

* identified as *A. cervicornis* in the field.

Ho observed heterozygosity He unbiased expected heterozygosity *F* IS inbreeding coefficient

Pairwise F_ST_ estimates were calculated using the Weir & Cockerham (1984) equation in the StAMPP R package (Pembleton, Cogan & Forster, 2013) with 100 bootstrap replicates to calculate 95% confidence intervals. Population genetic statistics estimations (H_e_, Ho, *F*_IS_ and nucleotide diversity *π*) were performed with either hierfstat R package (Goudet, 2005) or SambaR (de Jong et al., 2021). Differences between observed and expected heterozygosity for all loci within a species or among regions within a species were tested using paired Student’s *t*-test. Differences in nucleotide diversity (*π*) between species was performed using a two-tailed Student’s *t*-test and among regions within a species using a 1-way ANOVA with a Tukey post hoc test. Analysis of molecular variance (AMOVA) was conducted by region and sub-region with *poppr.amova* function of the poppr R package (Kamvar, Tabima & Grünwald, 2014) using the absolute genetic distance matrix calculated by the *bitwise.dist* function with 9,999 random permutations. Isolation by distance was evaluated within Colombia using the *mantel.rtest* function in the ade4 R package (Bougeard & Dray, 2018) with 9999 random permutations by correlating the pairwise *F*_ST_ genetic distances (*F*_ST_/(1- *F*_ST_)) with geographic distances. Geographic distances were computed from the latitude and longitude of the collection sites using the *distm* function of the geosphere R package (Hijmans, 2019).

To assess population structure of the two species, two methods were used: discriminant analysis of principal components (DAPC) and ADMIXTURE v1.3.0 (Alexander, Novembre & Lange, 2009). DAPC was performed using the adegenet R package (Jombart & Ahmed, 2011). The optimal number of clusters (K) was identified based on the smallest BIC value from 10 replicate runs of k-means clustering over a range of two to ten groups. The membership probability for each sample was calculated using the optimal K. An unsupervised ADMIXTURE analysis was run on populations of K ranging from two to ten with 20 replicates each. The inferred K with the lowest cross-validation error was chosen. ADMIXTURE replicates were combined and merged using the CLUMPAK server (Kopelman et al., 2015). For samples collected within Colombia, a Principal Components Analysis was performed using the *glPCA* function in the adegenet R package (Jombart & Ahmed, 2011).

To identify loci underlying the differentiation of the collection sites within Colombia for each species, we performed outlier loci analysis using PCAdapt v4.3.3 (Privé et al., 2020), Bayescan v2.1 (Foll & Gaggiotti, 2008) and OutFLANK v0.2 (Whitlock & Lotterhos, 2015) on all genotyping loci (*n* = 19,694). PCAdapt correlates each loci with the principal components (axes) retained from a Principal Component Analysis. The number of components retained is equal to the number of PCs that explain the largest proportion of genetic variation. Loci were additionally filtered for linkage disequilibrium (LD.clumping = list (size = 200, thr = 0.2)) and minor allele frequency threshold of 5% for the PCAdapt analysis with *K* = 2. *P*-values of loci with significant correlations were transformed into q-values using the qvalue R package (Storey et al., 2020) with a False Discovery Rate (FDR) threshold of 1%. OutFLANK is an R package that uses trimmed F_ST_ values to infer the distribution of F_ST_ for neutral markers and assigns q-values to each locus (Whitlock & Lotterhos, 2015). Finally, the Bayesian method Bayescan was run with default settings (burn in = 50,000, sample size = 5,000, iterations = 100,000) while testing for differences in allele frequencies between regions within Colombia. A FDR threshold of 5% was used for Bayescan and OutFLANK. Candidate outlier loci and their potential functional effects were identified using SNPeff v4.3 (Cingolani et al., 2012) with the *Acropora digitifera* genome (NCBI accession GCF_000222465.1, Shinzato et al., 2011).

## Results

**Figure 2:**
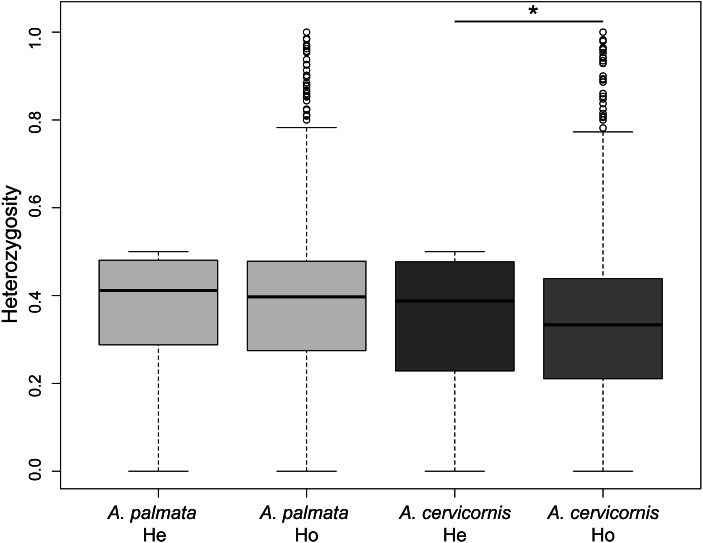
Genetic diversity estimates of *Acropora palmata* and *A. cervicornis* from Colombia. The observed heterozygosity for each locus was significantly lower than expected for *A. cervicornis*. Asterisk above the paired estimates indicate a significant *p*-value for a Student’s paired *t*-test.

Of the 186 colonies sampled, we identified unique multi-locus genotypes (MLG) of 57 *A. cervicornis*, 69 *A. palmata* and 3 hybrids (Table 1). The genetic diversity, measured as the difference in observed heterozygosity to expected heterozygosity, was significantly lower for *A. cervicornis* but not *A. palmata* samples (Student’s paired *t*-test, *A. cervicornis t* = 16.691, *Df* = 6200, *p* < 0.001; *A. palmata t* =  − 3.0964, *Df* = 7077, *p* = 0.999; Fig. 2). However, genetic diversity was significantly lower for specific regions in both *A. cervicornis* and *A. palmata* (Table 1). Consistent with these results, there was a small increase in homozygosity (*F*_IS_) for all *A. cervicornis* subpopulations and some of the *A. palmata* subpopulations (Rosario Islands, Urabá Gulf, and San Andrés, Table 1) suggesting differences in population declines between the species and among the regions. The nucleotide diversity, another metric of genetic diversity, also significantly differed among regions in both species (Fig. S1). The highest nucleotide diversity for *A. cervicornis* was in Urabá Gulf and lowest in TNNP whereas the highest nucleotide diversity for *A. palmata* was in San Andrés and lowest in San Bernardo.

### *Acropora cervicornis* clonal structure

All pairwise comparisons of *F*_ST_ between Colombia and Caribbean collection sites were significant but varied regionally, ranging from 0.069 with Florida to 0.243 with Puerto Rico (Table 2). Significant differentiation between the populations and among the regions within the populations was detected, explaining 9.81% of the total variance in allelic frequencies (AMOVA Df = 3, sum of squares = 2.994, *p* = 0.0001; Table 3). Within Colombia, the most divergent site was San Andrés with an average pairwise *F*_ST_ value of 0.124 between neighboring regions. Colonies sampled from San Bernardo Islands were not significantly different from neighboring collection sites of Rosario Islands, I. Arena and TNNP (Table 2). We found a significant positive correlation between geographic distance and genetic distance within the Colombian reefs (Mantel Test, *r* = 0.910, *p* = 0.022).

**Table 2:** Pairwise F_*ST*_ values between regions and within Colombia. Weir & Cockerham (1984) pairwise F_*ST*_ values (above the diagonal) and *p*-values (below the diagonal, red text = not significant) were calculated using the R package StAMPP. Average pairwise F_*ST*_ values are for within Colombia comparisons only.

	San Andrés	Urabá Gulf	San Bernardo	Rosario	Cartagena	I. Arena	TNNP	Belize	Cuba	Florida	Curacao	Puerto Rico	USVI	Avg. *F*_ST_
*A. cervicornis*														
San Andrés		0.109	0.137	0.138		0.140	0.139	0.142	0.140	0.069	0.114	0.104	0.103	0.124
Urabá Gulf	0.000		0.013	0.014		−0.008	0.013	0.118	0.098	0.130	0.112	0.168	0.168	0.020
San Bernardo	0.000	0.000		−0.001		0.000	0.002	0.158	0.166	0.176	0.157	0.239	0.239	0.030
Rosario	0.000	0.000	**0.730**			0.010	0.003	0.159	0.178	0.177	0.160	0.238	0.239	0.033
Cartagena														
I. Arena	0.000	0.000	**0.950**	**0.560**			0.002	0.139	NA	0.166	0.163	0.257	0.254	0.028
TNNP	0.000	0.000	**0.8**	0.000		0.030		0.159	0.171	0.176	0.147	0.243	0.232	0.032
Belize	0.000	0.000	0.000	0.000		0.000	0.000		0.121	0.101	0.175	0.203	0.190	–
Cuba	0.000	0.000	**0.080**	**0.050**		**0.350**	0.000	0.000		0.117	0.151	0.187	0.172	–
Florida	0.000	0.000	0.000	0.000		0.000	0.000	0.000	0.000		0.166	0.164	0.156	–
Curacao	0.000	0.000	0.000	0.000		0.000	0.000	0.000	0.000	0.000		0.181	0.161	–
Puerto Rico	0.000	0.000	0.000	0.000		0.000	0.000	0.000	0.000	0.000	0.000		0.012	–
USVI	0.000	0.000	0.000	0.000			0.000	0.000	0.000	0.000	0.000	0.000		–
*A. palmata*														
San Andrés		0.049	0.046	0.047	0.055	0.054	0.048	0.112		0.063	0.082	0.085	0.069	0.050
Urabá Gulf	0.000		0.005	0.003	0.007	0.007	0.007	0.124		0.098	0.139	0.147	0.149	0.013
San Bernardo	0.000	0.000		0.013	0.019	0.001	0.001	0.117		0.090	0.131	0.142	0.151	0.015
Rosario Islands	0.000	0.000	0.020		−0.001	0.002	0.008	0.121		0.089	0.119	0.143	0.144	0.012
Cartagena	0.000	0.000	0.000	**0.650**		0.009	0.015	0.120		0.087	0.131	0.147	0.159	0.017
I. Arena	0.000	0.000	**0.050**	**0.180**	0.000		−0.002	0.121		0.095	0.136	0.158	0.158	0.012
TNNP	0.000	0.000	**0.050**	0.000	0.000	**0.990**		0.122		0.090	0.136	0.147	0.152	0.013
Belize	0.000	0.000	0.000	0.000	0.000	0.000	0.000			0.026	0.160	0.169	0.160	–
Cuba														
Florida	0.000	0.000	0.000	0.000	0.000	0.000	0.000	0.000			0.108	0.115	0.093	–
Curacao	0.000	0.000	0.000	0.000	0.000	0.000	0.000	0.000		0.000		0.073	0.046	–
Puerto Rico	0.000	0.000	0.000	0.000	0.000	0.000	0.000	0.000		0.000	0.000		0.028	–
USVI	0.000	0.000	0.000	0.000	0.000	0.000	0.000	0.000		0.000	0.000	0.000		–

**Table 3:** Analysis of Molecular variance (AMOVA) among and within sampling sites and regions.

Species	Source of variation	*Df*	Sum of squares	Variance %	Fixation indices
*A. cervicornis:*	Among regions	6	2.883	16.66	0.026^*^
by geographic regions^a^	Among samples within regions	114	15.128	83.33	
	Total	120	18.011	100	
by DAPC assigned populations^a^	Among populations	3	2.994	9.81	0.015^*^
	Among regions within populations	5	1.008	10.98	0.017^*^
	Among samples within regions	112	14.010	79.21	0.125^*^
	Total	120	18.011	100	
*A. palmata:*	Among regions	5	3.854	15.969	0.028^*^
by geographic regions^a^	Among samples within regions	153	22.153	84.031	
	Total	158	26.007	100	
by DAPC assigned populations^a^	Among populations	2	2.878	8.91	0.015^*^
	Among regions within populations	4	1.148	7.88	0.014^*^
	Among samples within regions	152	21.981	83.21	0.144^*^
	Total	158	26.007	100	

**Notes.**

* Significance at alpha= 0.05 based on 9,999 permutations.

a Colombia collection sites were combined.

**Figure 3:**
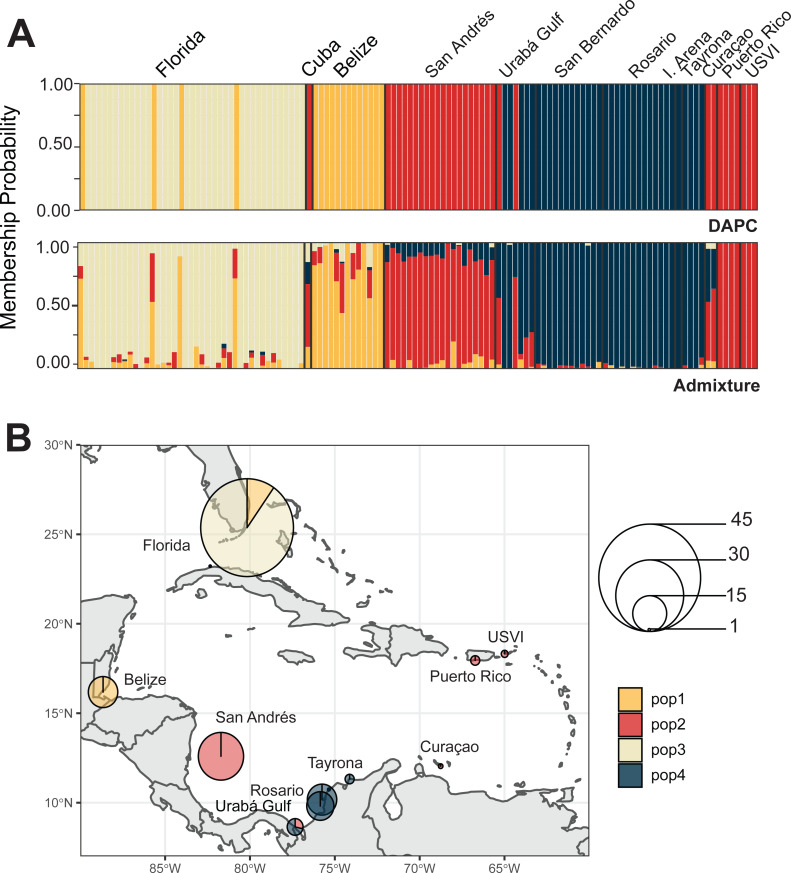
Population assignments for *Acropora cervicornis* across the wider Caribbean region. (A) DAPC, on top, and ADMIXTURE, on bottom, assignments for 121 unique *A. cervicornis* MLGs. Columns represent individual samples and their associated probability of assignment to *K* = 4 color-coded genetic clusters (pop1 = yellow, pop2 = red, pop3 = tan, pop4 = blue). Additional Ks are presented in Supplemental Figs. S4 and S5). (B) Pie charts of the population assignments for each collection region based on the DAPC results above. Circle sizes are proportional to the number of samples analyzed.

We identified four *A. cervicornis* populations across the Caribbean in agreement with previous studies (Vollmer & Palumbi, 2007; Hemond & Vollmer, 2010; Drury et al., 2016). Both DAPC and ADMIXTURE analyses supported inferred population cluster size of four (Fig. 3A and Fig. S2). The first population is composed of Cuba (*n* = 1), San Andrés (*n* = 20), Urabá Gulf (*n* = 2), Curaçao (*n* = 2), Puerto Rico (*n* = 4) and USVI (*n* = 3). The second is composed solely of collection sites within Colombia, including Urabá Gulf (*n* = 5), San Bernardo Islands (*n* = 12), Rosario Islands (*n* = 13), Isla Arena (*n* = 1) and TNNP (*n* = 4). There are two populations in Colombia with Urabá Gulf harboring *A. cervicornis* colonies from both San Andrés and the other Eastern Colombian locations; there is also evidence of gene flow between the two populations (Fig. 3B and Figs. S3–S5). Moreover, Curaçao samples also show evidence of admixture between the populations in the East (Puerto Rico and USVI) and samples from Colombia (Fig. 3B).

**Figure 4:**
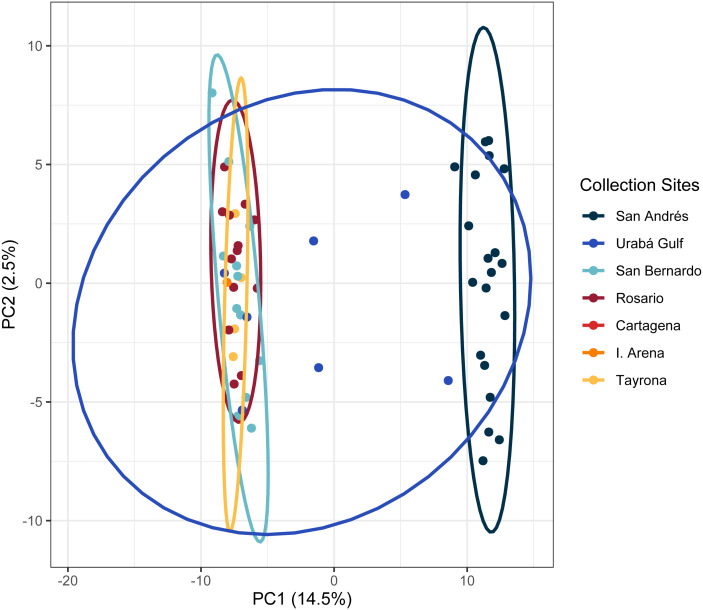
Principal components for *Acropora cervicornis* Colombian Caribbean populations. Locations are shown by different colors and dots represent individuals.

In the regional analysis, Principal Components Analysis (PCA) showed Urabá Gulf as a transitional zone between San Andrés and the other localities and variable population separation, with San Andrés separating from the cluster of Rosario Island, San Bernardo Islands, and TNNP (Fig. 4). Overlap between Rosario Island, San Bernardo Islands, and TNNP supported similarities based on pairwise *F*_ST_ values, although patterns of structure in the *F*_ST_ comparisons between TNNP and Rosario Islands populations are not apparent.

Between the two populations within Colombia, we identified 584 and 260 candidate loci under divergent selection with PCadapt and Bayescan, respectively (Table S2). Both methods shared 165 loci. Of these, 65 loci were located within 58 genes (*n* = 33 intronic, *n* = 32 exonic) and seven loci were predicted as missense mutations (Table S2). These missense mutations fall in genes that function in transmembrane transport (major facilitator superfamily domain-containing protein 12-like), Golgi organization (golgin subfamily B member 1-like), protein turnover (leucyl aminopeptidase) and uncharacterized functions (*n* = 4).

### *Acropora palmata* clonal structure

Pairwise *F*_ST_ estimates between Colombia and Caribbean collection sites were significant (Table 2). Allelic frequencies differed moderately between populations (AMOVA, *Df* =2, sum of squares = 2.878, *p* = 0.0193) and regions within populations (AMOVA, *Df* = 4, sum of squares = 1.148, *p* = 0.001; Table 3). Similar to *A. cervicornis*, San Andrés was the most divergent site with an average pairwise *F*_ST_ value of 0.050 between neighboring collection sites. Colonies sampled from San Bernardo Islands were not significantly different from neighboring collection sites of TNNP, and colonies from Cartagena were not significantly different from those in Rosario Islands (Table 2). Unlike *A. cervicornis*, we found no significant correlation between geographic distance and genetic distance within the Colombian reefs for *A. palmata* (Mantel Test, *r* = 0.876, *p* = 0.091).

**Figure 5:**
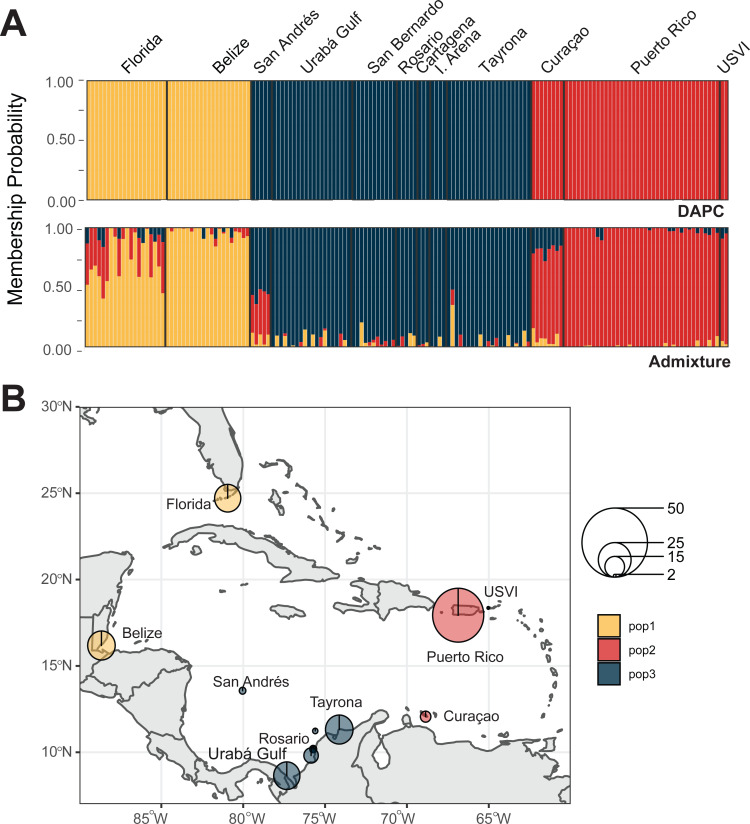
Population assignments for *Acropora palmata* across the wider Caribbean region. (A) DAPC, on top, and ADMIXTURE, on bottom, assignments for 159 unique *A. palmata* MLGs. Columns represent individual samples and their associated probability of assignment to *K* = 3 color-coded genetic clusters (pop1 = yellow, pop2 = red, pop3 = blue). Additional Ks are presented in Supplemental Figs. S8 and S9. (B) Pie charts of the population assignments for each collection region based on the DAPC results above. Circle sizes are proportional to the number of samples analyzed.

We identified three *A. palmata* populations across the Caribbean regions. Both DAPC and ADMIXTURE analyses supported inferred population cluster size of three (Fig. 5A and Figs. S6–S9). However, cross-validation error was only marginally higher for *K* = 4 with ADMIXTURE (Fig. S6), which separated Curaçao from Puerto Rico and USVI samples (Fig. S9). One population was found to encompass all collection sites from Colombia (Fig. 5B). However, low levels of gene flow were evident between the Eastern population and the Colombian population in samples from San Andrés and Curaçao (Fig. S7 and S9).

**Figure 6:**
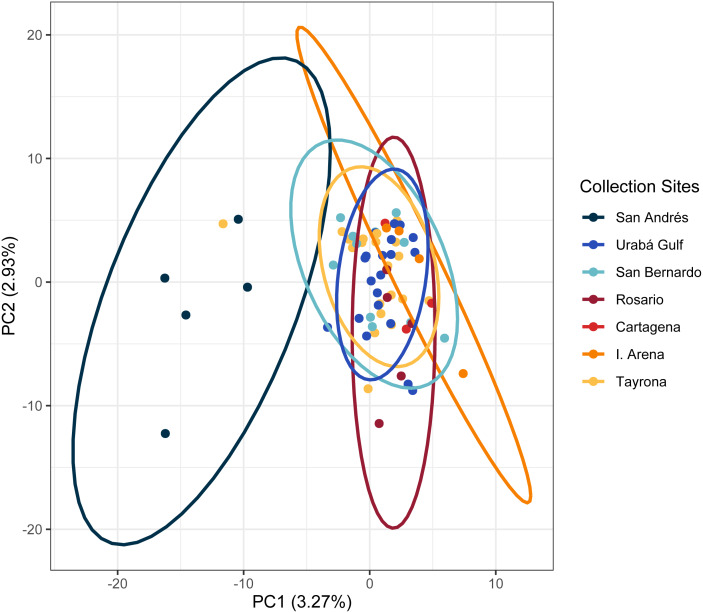
Principal components for *Acropora palmata* Colombian Caribbean populations. Locations are shown by different colors and dots represent individuals.

The PCA in the regional analysis showed San Andrés separating from the other localities similar to *A. cervicornis*, but not nearly as far in variance on PC1 and also separating from the core cluster overlapping Urabá Gulf, Rosario Islands, Cartagena, San Bernardo Islands, I. Arena and TNNP (Fig. 6). Overlap between Rosario Islands with Cartagena, and San Bernardo Islands with TNNP supported similarities based on pairwise *F*_ST_ values, although patterns of structure in the *F*_ST_ comparisons between Urabá Gulf and the rest of populations are not apparent.

We identified 32 and 17 candidate loci under selection with PCadapt and OutFLANK, respectively (Table S3). Eleven loci were shared between the two analyses, five of which were found within intragenic sequences (*n* = 3 intronic, *n* = 2 exonic) (Table S3).

## Discussion

The population structure of *Acropora* species in the Caribbean has been broadly defined between the two Eastern and Western provinces (Baums, Miller & Hellberg, 2005; Baums, Miller & Hellberg, 2006; Vollmer & Palumbi, 2007; Hemond & Vollmer, 2010; Mège et al., 2014; Porto-Hannes et al., 2015; Devlin-Durante & Baums, 2017; Kitchen et al., 2020). Based on these observations, we expected to find separation of populations somewhere in the southwest Caribbean between Panama and Colombia (Baums, Miller & Hellberg, 2005; Porto-Hannes et al., 2015). Instead, based on the *Acropora* spp. samples from Colombia we revealed no separation in the Southwestern Caribbean; rather we identified isolated populations in both species, although with different patterns of genetic flow. For *A. palmata* we found one population to encompass all Colombia collection sites with low levels of gene flow. In contrast, *A. cervicornis* harbors two populations with reefs in Urabá Gulf (Capurganá) having colonies from both populations, and evidence of gene flow between Eastern Caribbean colonies and Colombia colonies. Moreover, Curaçao samples also show evidence of admixture between the populations in the Northeast (Puerto Rico and USVI) and samples from Colombia.

The contrasting result between the geographical distance and the genetic distance within the reefs of Colombia, significant for *A. cervicornis*, but not for *A. palmata* is important in terms of the resilience and conservation of the species, because the status of *A. palmata* is currently better in the Colombian Caribbean, with formations in very good condition, while for *A. cervicornis* the condition is critical (García-Urueña & Garzón-Machado, 2020). We found no significant isolation-by-distance in *A. palmata*, but with a small number of candidate loci under selection (11 shared between PCadapt and OutFLANK), which could indicate that a higher priority management is required for this species. In *A. cervicornis*, isolation-by-distance was detected and between 260 and 584 candidates were identified under divergent selection, sharing 165 loci among the methods used (PCAdapt and Bayescan). The presence of two distinct subpopulations in *A. cervicornis* poses long term conservation risks for the species. Reduced gene flow, in addition to the confirmed poor state of *A. cervcornis* thickets in Colombia, may jeopardize the resilience of the species under different stress scenarios. However, this scenario could indicate a greater risk for *A. palmata* in terms of this species being considered in the Colombian Caribbean as an isolated region, genotypically impoverished, which could generate potentially a lower response of the species to local and global environmental stressors. Palumbi (2003) proposed that self-seeding and isolated populations, implies higher vulnerability to disturbance events, as recovery is reliant on local survivors. Therefore, to protect this species more effectively, each population should be managed independently regardless of geographic proximity.

In both species, the genetic diversity estimated was globally much lower (mean H_E_ per site = 0.321 ± 0.012 in *A. palmata* and 0.369 ± 0.012 in *A. cervicornis*) than previous studies. Specifically for *A. palmata,* Baums, Miller & Hellberg (2005) estimated values of H_E_ between 0.58–0.85, Mège et al. (2014) obtained 0.761, Porto-Hannes et al. (2015) between 0.797 and 0.900 and (Japaud et al., 2019) reported 0.79. These results together with the genetically isolated Colombian acroporid populations should be a concern because genetic diversity is necessary to species adaptation success facing changes in environmental conditions (Miller & Ayre , 2004; Japaud et al., 2019). The low gene flow across the Colombian Caribbean is a possible indication that the genetic diversity present in this population is not sufficient to allow sexual reproduction via outcrossing. Hemond & Vollmer (2010) mentioned that sufficient genetic diversity and larval recruitment are essential for recovery of at risk populations of corals, but our results indicate that genetic diversity (H_E_ = 0.389) is less in the rest of Caribbean (H_E_ = 0.701 ± 0.043); a result supporting the assertion by Baums, Miller & Hellberg (2006), that the Western Caribbean populations are genotypically depauperate.

The joint study of regional circulation patterns related to the genetic connectivity of populations has shown similar patterns of clusters throughout the Caribbean. Foster et al. (2012) showed three populations clusters in the scleractinian coral *Orbicella annularis*: an Eastern cluster (Lesser Antilles, Venezuela and Curaçao), a Western cluster (The Bahamas Archipelago, Cuba, Belize and Cayman Islands) and a central cluster (Jamaica, Honduras, Nicaragua, Colombia, Puerto Rico, BVI and Dominican Republic). Further, they mentioned that the southern extent of the East–West barrier to gene flow across the Caribbean appears to lie between the Venezuelan corridor to the east and the Colombia-Panama gyre to the west and the barrier may be the plume of low salinity runoff from the Magdalena River that lies just north of Cartagena and discharges up to 228 km^3^ of sediment-laden water into the Caribbean Sea annually, according with Restrepo & Kjerfve (2000). Our results suggest that sediment discharge from the Magdalena River does not act as a barrier to larval dispersal, not only because we showed a different population structure in the Colombian Caribbean, but also because both species have a similar link between the San Bernardo Islands and the TNNP (*F*_ST_ = 0.002, *P* > 0.130 in *A. cervicornis*, and *F*_ST_ = 0.001, *P* > 0.070 in *A. palmata*), precisely these localities are separated by the mouth of the Magdalena river (11°06′21.81N, 75°50′07.31W); therefore there has been connectivity between these populations.

*Acropora cervicornis* are regionally connected, and readily share genetic information between Curaçao, San Andrés and in general in the Western Caribbean. According to Tang et al. (2006), the near-surface (5 m) circulation in the Western Caribbean Sea is characterized by a persistent throughflow of the Caribbean Current, which is relatively broad and roughly westward in the central and Eastern Colombian Basin. This current bifurcates before reaching the Nicaragua Rise, with a weak branch veering southwestward to form the cyclonic, highly variable Panama-Colombia Gyre in the southwestern Caribbean Sea. Oceanographic models demonstrate that westward larval dispersal, throughout the Caribbean Current, enters the basin through the southern Lesser Antilles in the East and travels west-northwest toward the Yucatan Peninsula (Richardson, 2005). The swiftest portion of this current creates a nearly direct corridor along the Venezuelan coast that passes near Curaçao en route to Mexico (Richardson, 2005). This pattern of connectivity in the western populations has been previously described for *Orbicella faveolata* between Curaçao and Mexico by Rippe et al. (2017), which may indicate a non-restricted larval dispersal (<500 km) as was observed in *A. cervicornis* by Vollmer & Palumbi (2007). Another particular aspect for this species is the evidence of gene flow between Curaçao and Colombia, this fact may be explained by the existence of an eastward flow from Panama to the Antilles, counter to the Caribbean Current (Andrade, Barton & Mooers, 2003). Finally, the evidence of connectivity between San Andrés and Urabá Gulf may be the result of the influence of the Colombia-Panama Gyre because most of its transport is recirculated in the Southwest Caribbean (Andrade, Barton & Mooers, 2003; Cowen, Paris & Srinivasan, 2006; Hemond & Vollmer, 2010; Foster et al., 2012; Porto-Hannes et al., 2015). Similar results of genetic connectivity of damselfish *Stegastes partitus* revealed evidence of gene flow among populations from the South Caribbean, including San Andrés, Urabá Gulf (Capurganá), Rosario Islands and Santa Marta (Ospina-Guerrero et al., 2008).

For *A. palmata* with only one population to encompass all Colombia collection sites indicates that regional and local circulation models can explain the connectivity in the western Caribbean, a result that is consistent with the previous study by Cowen, Paris & Srinivasan (2006), who mentioned that the reefs along the Panama-Colombia gyre are isolated from the rest of the Caribbean. This result also indicates that this coral species may be of particular conservation concern due to its relative isolation, and limited dispersal in the Caribbean (Porto-Hannes et al., 2015).

In this context the hypothesis of genetic adaptation of *A. palmata* colonies to local and specific environmental conditions (Baums, 2008; Devlin-Durante & Baums, 2017) is important for the TNNP. Although this locality contains the largest and most important Colombian population of this species (García-Urueña & Garzón-Machado, 2020; García-Urueña, Garzón-Machado & Sierra-Escrigas, 2020), it is the only one exposed to a seasonal upwelling (December–April) with temperature changes between 23 °C and 30 °C (Bayraktarov, Pizarro & Wild, 2014), indicating their potential adaptation to these seasonally colder water conditions. The approach to consider populations at smaller geographic scales highlights the importance for local management and restoration strategies. In this particular case, TNNP may not be a good source for transplanting colonies to other locations, despite harboring an important population for *A. palmata*. If site-specific adaptations have risen in the *A. palmata* population, their fitness may be suboptimal in locations with different environmental conditions. Further studies are needed in the Colombian Caribbean and adjacent regions because patterns of population structure in corals are complex and influenced by numerous factors (Severance & Karl, 2006).

An AMOVA detected significant differentiation between regions explaining 16.66% in *A. cervicornis* and 15.96% in *A. palmata* of the total variance in allelic frequencies (*p* < 0.001; Table 2). Likewise, genetic structure was found locally and pairwise comparisons revealed significant *F*_ST_ values for the majority of comparisons, indicating low to moderate differentiation among populations. However, interpreting the biological relevance of low, but statistically significant *F*_ST_ values is a challenge, especially in marine populations (Porto-Hannes et al., 2015). Although in both species the most divergent locality was San Andrés (*F*_ST_: 0.131 in *A. cervicornis* and 0.050 *in A. palmata*) an indication of the diversity within populations, the He values were very low in both species, which it also indicates a low level of differentiation throughout the Colombian Caribbean. Therefore, these data suggest that populations of *A. palmata* and *A. cervicornis* throughout the Colombian Caribbean share little genetic information.

Over 35 years have passed since the disease-induced mass mortalities in acroporids and population recovery is slow, moderate and local (*e.g.*, Muller, Rogers & van Woesik, 2014; Lucas & Weil, 2016; Busch et al., 2016; D’Antonio, Gilliam & Walker, 2016). In Colombia, there are significant *Acropora palmata* stands whereas *A. cervicornis* stands are uncommon and even absent. We are reporting that the Colombian acroporids harbor significantly lower genetic diversity than in other areas and that the sampled populations are relatively isolated leading us to raise concerns about their conservation status. Significant efforts are being made to understand the early stages of development of *Acropora* species and the induction of coral settlement in order to improve post-settlement survival (Gómez-Lemos & García-Urueña, 2022; Rada-Osorio, Gómez-Lemos & García-Urueña, 2022), in addition to important restoration efforts based on propagation by fragmentation with both species in the Rosario Islands, the San Andrés Archipelago, Old Providence and Santa Catalina, the McBean National Natural Park and in the TNNP with demonstrated success. However, this short-term solution must also be accompanied by efforts in restoration through sexual reproduction, identification of genetically resistant individuals to stressors, and implementation of specific management programs for each species given the idiosyncrasies of each place. Scientifically informed restoration efforts (Baums et al., 2019; Parkinson et al., 2020; Shaver et al., 2021), coupled with sensible management plans (Shaver et al., 2021) may offer the best chance for the long-term survival of Colombian acroporids.

##  Supplemental Information

10.7717/peerj.13854/supp-1 Supplemental Information 1 Nucleotide diversity for *Acropora cervicornis* and *A. palmata* Lowercase letters that are not shared indicated significant differences between groups using a 1-way ANOVA with post hoc Tukey tes.

10.7717/peerj.13854/supp-2 Supplemental Information 2 Optimal number of groups detected by DAPC (A) and ADMIXTURE (B) for *A. cervicornis*

10.7717/peerj.13854/supp-3 Supplemental Information 3 PCA (A) and DAPC (B) plots for *A. cervicornis*

10.7717/peerj.13854/supp-4 Supplemental Information 4 DAPC clustering for inferred *A. cervicornis* populations from two to six

10.7717/peerj.13854/supp-5 Supplemental Information 5 ADMIXTURE clustering for inferred *A. cervicornis* populations from two to six

10.7717/peerj.13854/supp-6 Supplemental Information 6 Optimal number of groups detected by DAPC (A) and ADMIXTURE (B) for *A. palmata*

10.7717/peerj.13854/supp-7 Supplemental Information 7 PCA (A) and DAPC (B) plots for *A. palmata*

10.7717/peerj.13854/supp-8 Supplemental Information 8 DAPC clustering for inferred *A. palmata* populations from two to six

10.7717/peerj.13854/supp-9 Supplemental Information 9 ADMIXTURE clustering for inferred *A. palmata* populations from two to five

10.7717/peerj.13854/supp-10 Supplemental Information 10 Database samples Colombia vs Caribbean

10.7717/peerj.13854/supp-11 Supplemental Information 11 Acropora cerviconis outliers PC1

10.7717/peerj.13854/supp-12 Supplemental Information 12 Acropora palmata outliers PC1

## Additional Information and Declarations

### Field Study Permissions

The following information was supplied relating to field study approvals (i.e., approving body and any reference numbers): National Natural Parks Unit of Colombia approved this study (permit 001, March 6, 2017).

### Data Availability

The following information was supplied regarding data availability: Data is available at GitHub: https://github.com/skitchen19/Colombian_acroporids
