# Supplementary figures and images for: Fine scale population structure of Acropora palmata and Acropora cervicornis in the Colombian Caribbean
Source: PeerJ. 2022 Aug 30;10:e13854. doi: 10.7717/peerj.13854 (PMC9438773; doi:10.7717/peerj.13854)

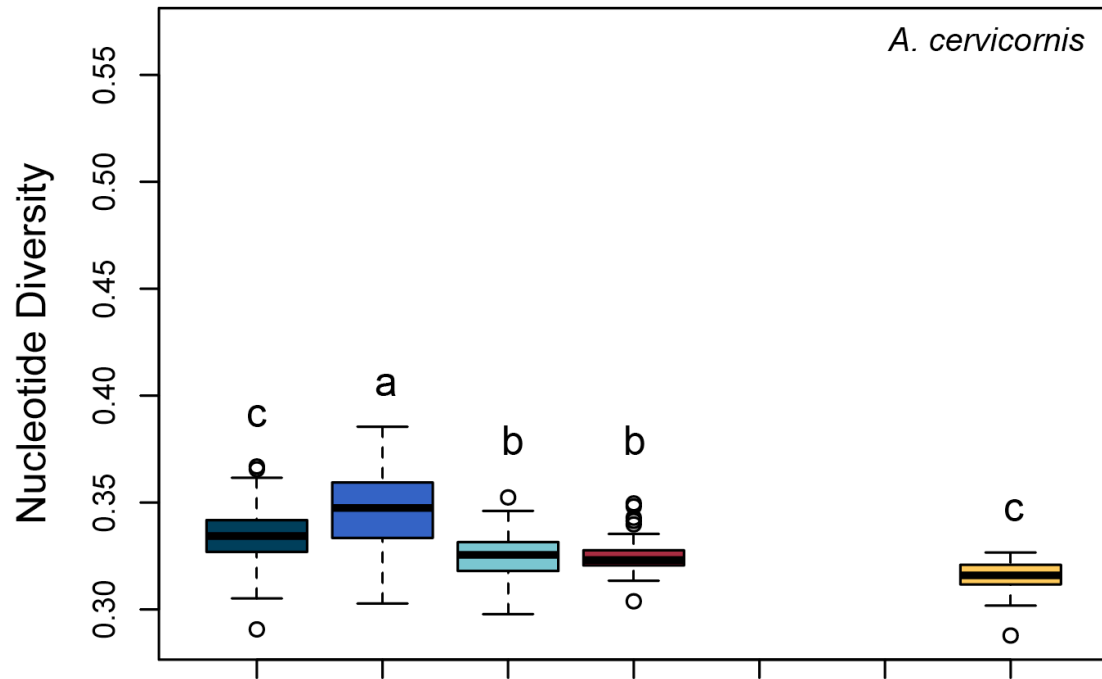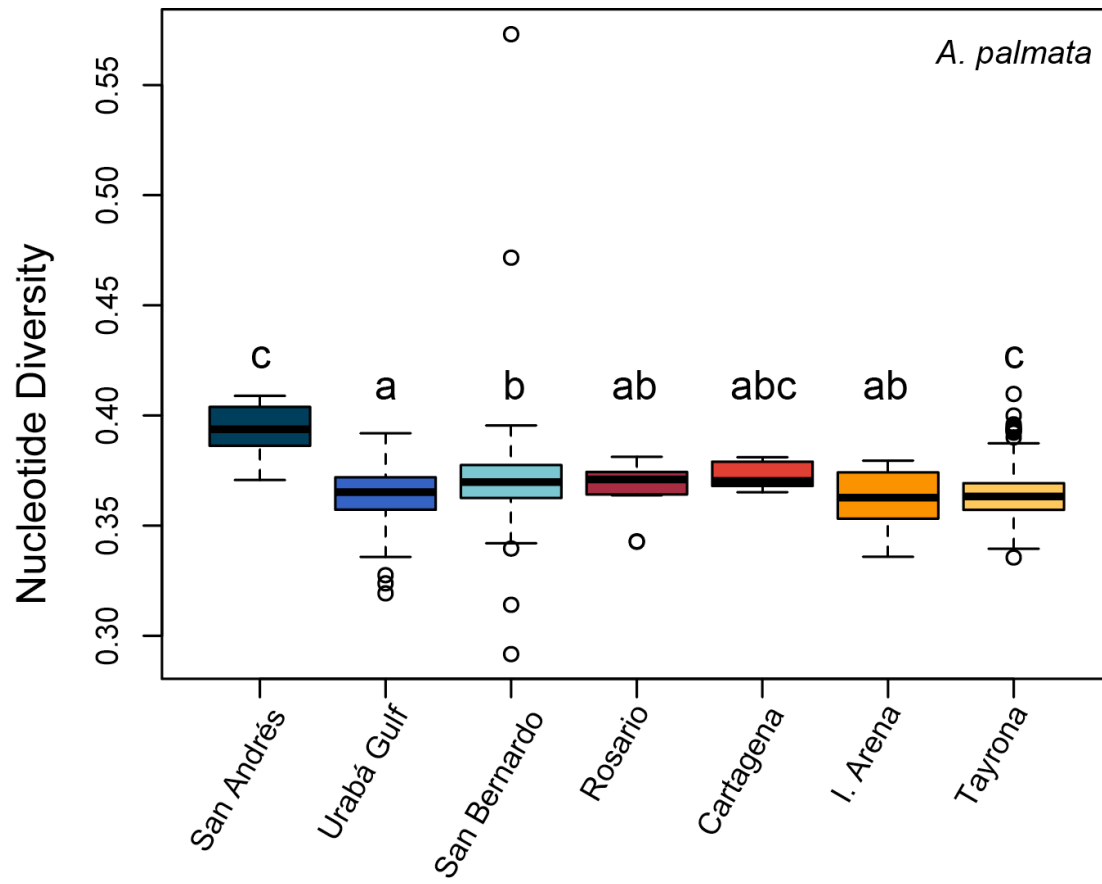

Supplement: Supplemental Information 1 — Lowercase letters that are not shared indicated significant differences between groups using a 1-way ANOVA with post hoc Tukey tes. [file peerj-10-13854-s001.pdf]

**A**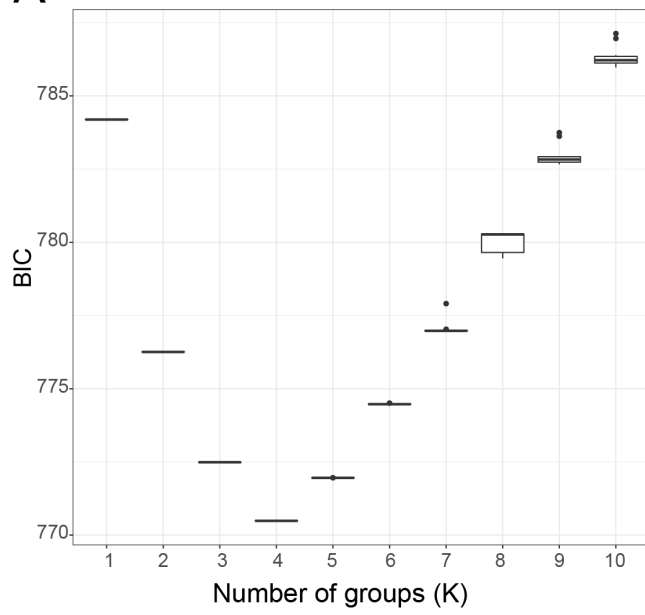**B**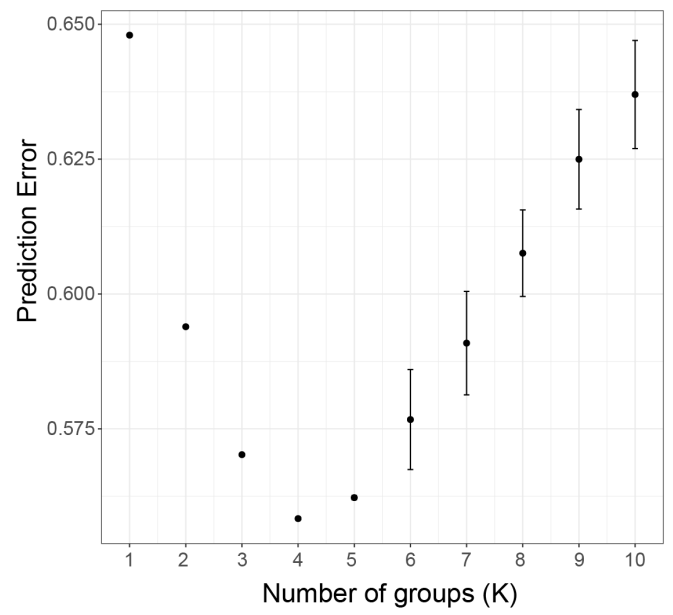

Supplement: Supplemental Information 2 [file peerj-10-13854-s002.pdf]

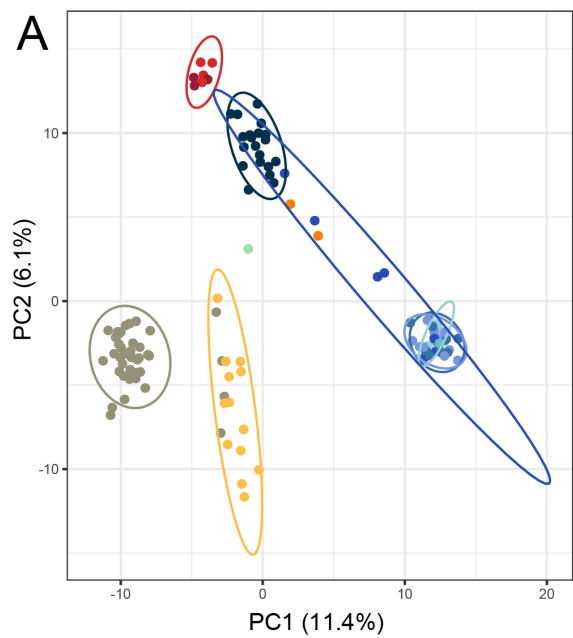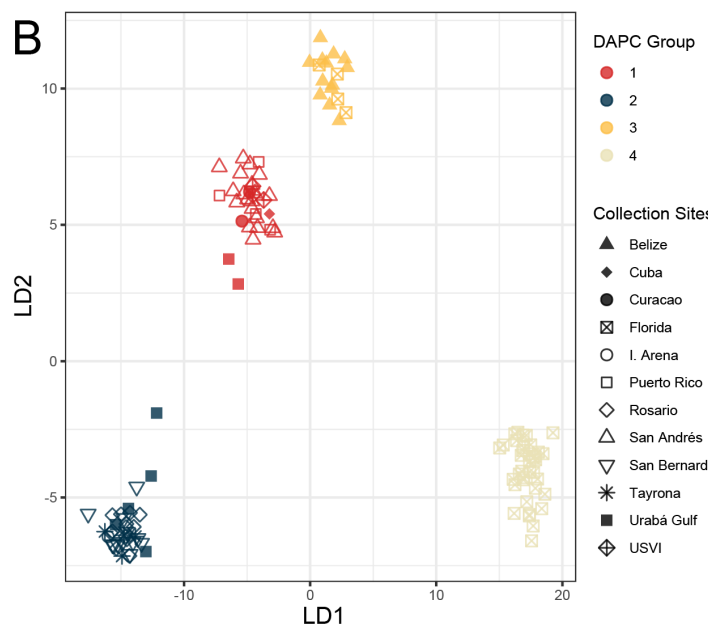

Supplement: Supplemental Information 3 [file peerj-10-13854-s003.pdf]

Posterior probability of group membership

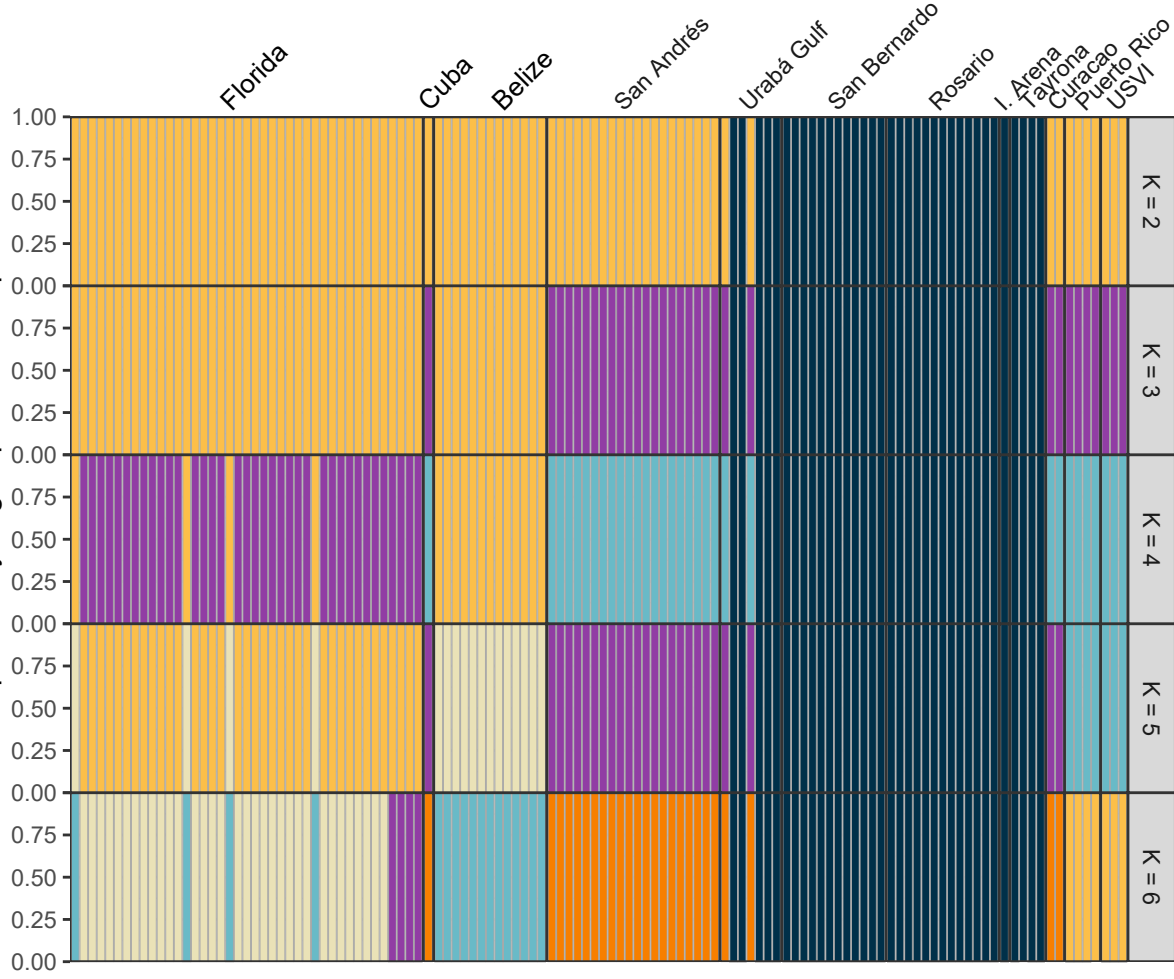

Supplement: Supplemental Information 4 [file peerj-10-13854-s004.pdf]

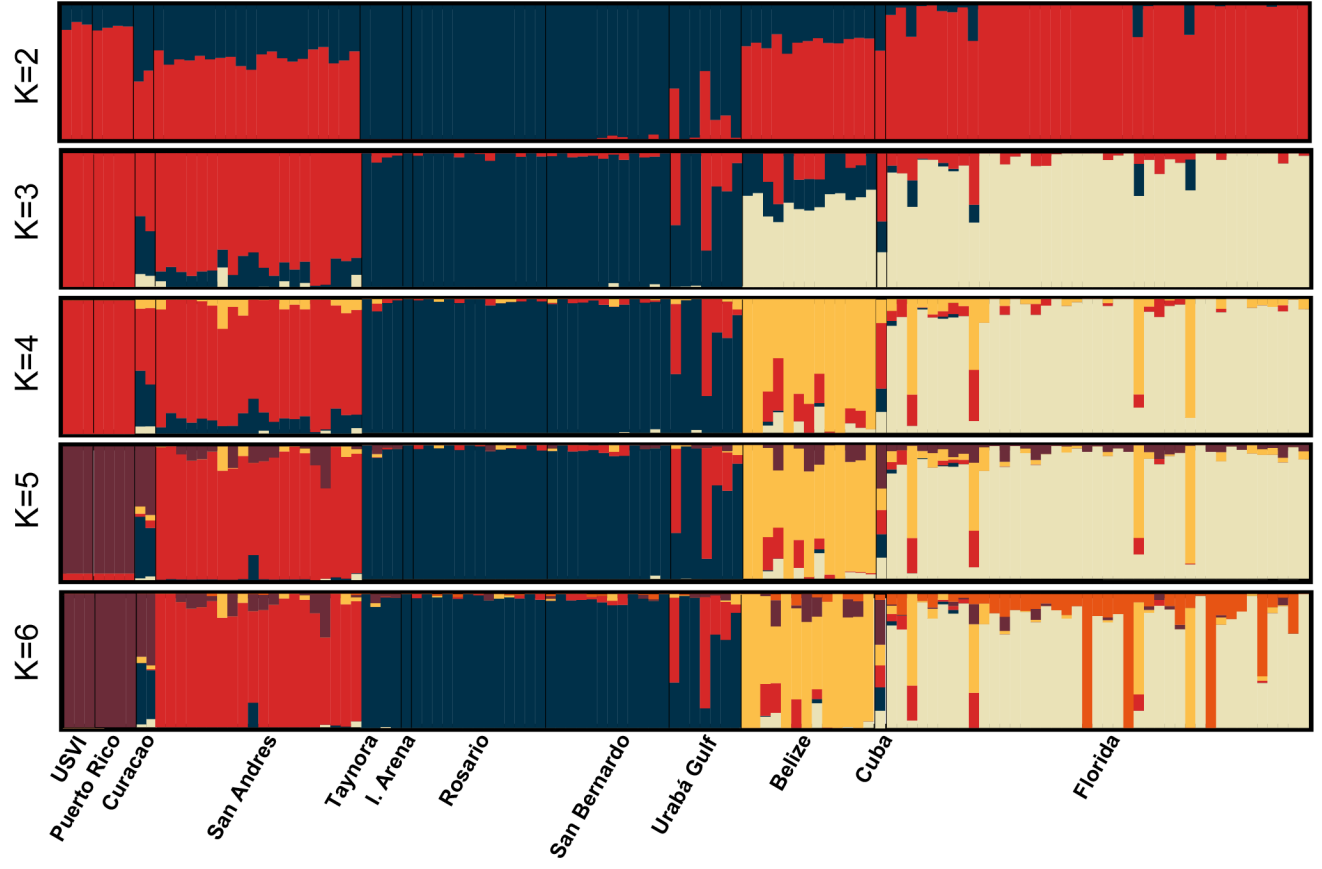

Supplement: Supplemental Information 5 [file peerj-10-13854-s005.pdf]

**A**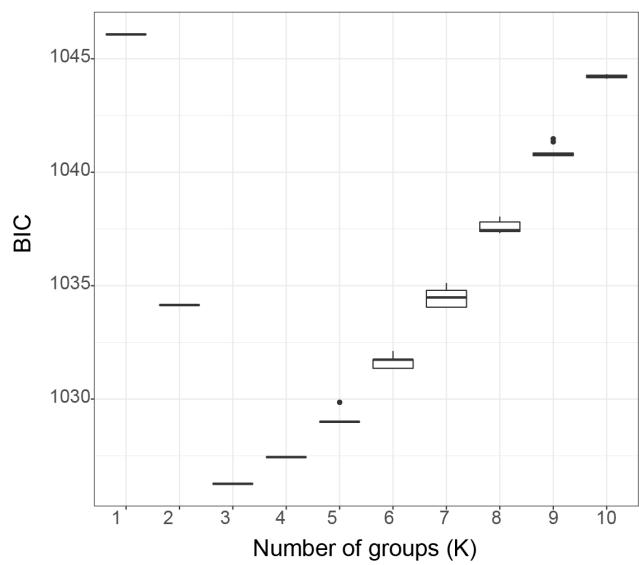**B**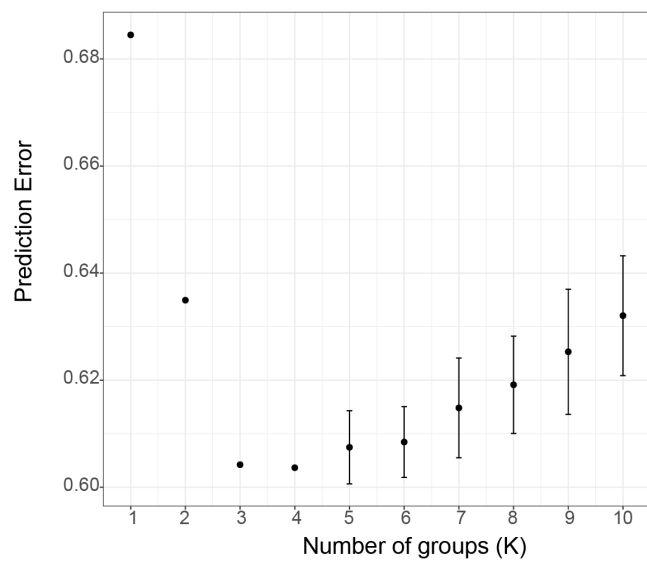

Supplement: Supplemental Information 6 [file peerj-10-13854-s006.pdf]

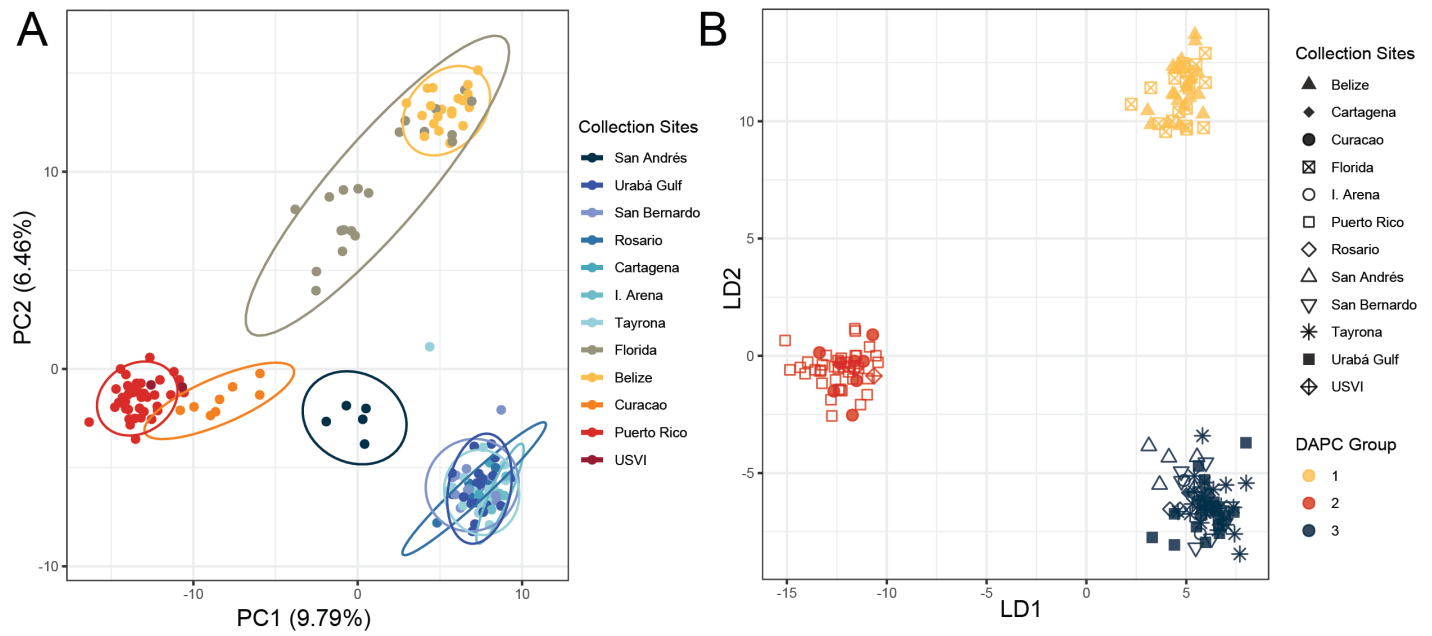

Supplement: Supplemental Information 7 [file peerj-10-13854-s007.pdf]

Posterior probability of group membership

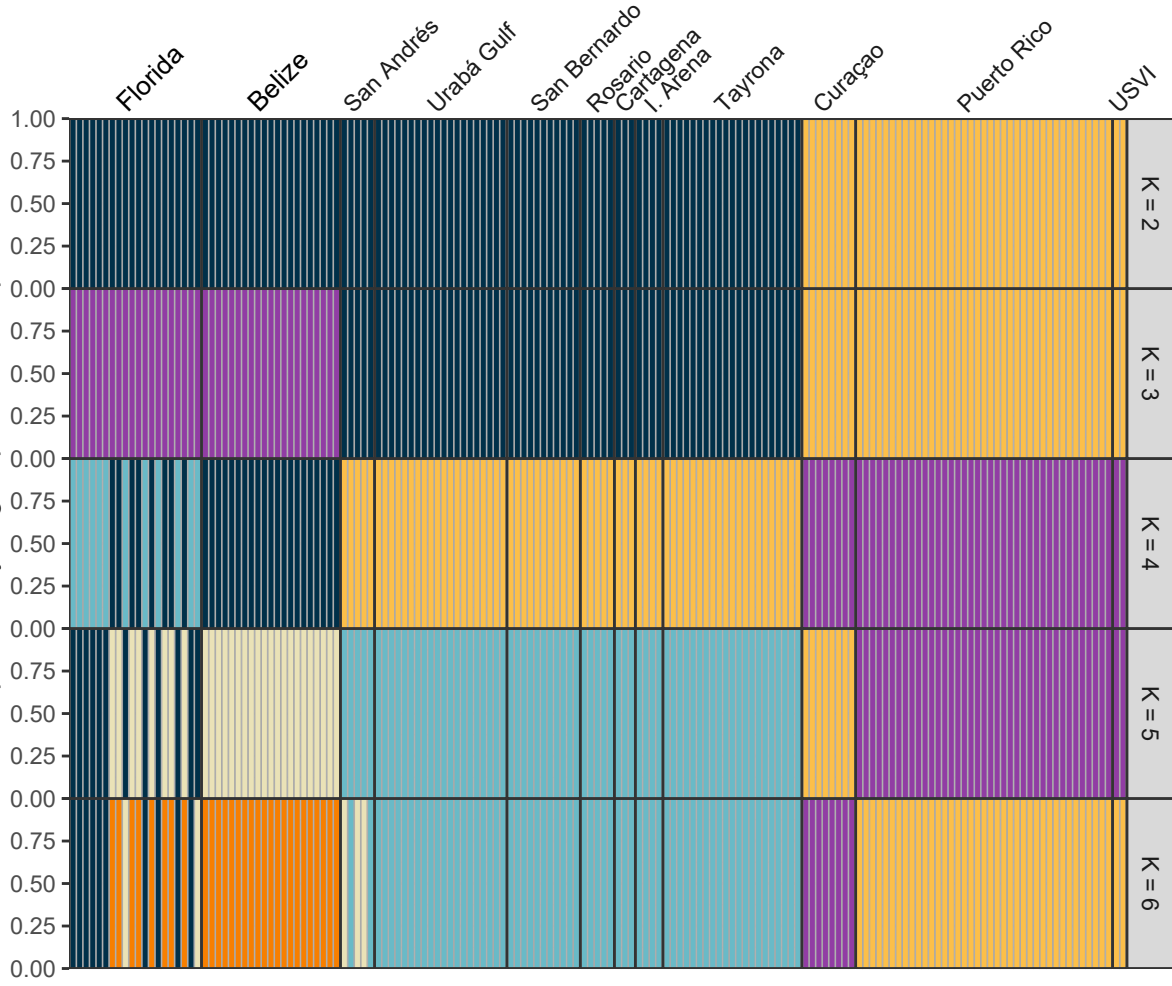

Supplement: Supplemental Information 8 [file peerj-10-13854-s008.pdf]

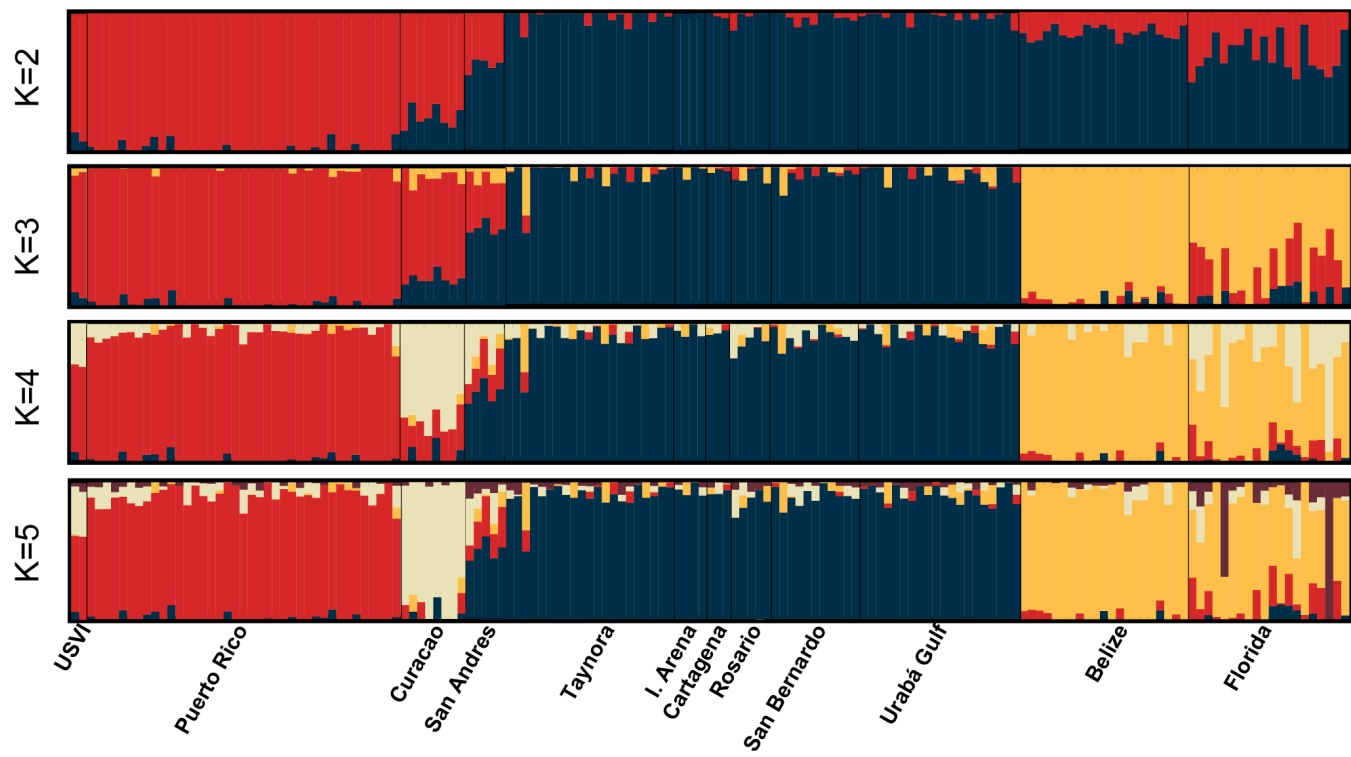

Supplement: Supplemental Information 9 [file peerj-10-13854-s009.pdf]
