# Supplementary material for: Fine scale population structure of Acropora palmata and Acropora cervicornis in the Colombian Caribbean
Source: PeerJ. 2022 Aug 30;10:e13854. doi: 10.7717/peerj.13854 (PMC9438773; doi:10.7717/peerj.13854)
Supplement: Supplemental Information 10 [file peerj-10-13854-s010.pdf]

Affy ID	Sample ID	Genetic Species	Region	Latitude	Longitude	Reef	Multi-Locus (	This Study
a550962-4383627-121220-367_K21.CEL	RG Ac43	A. cervicornis	San Bernardo	9,80244	-75,81946	La Pared	HG0173	VERDADERO
a550962-4383627-121220-367_M11.CEL	RG Ac57	A. prolifera	Capurganá	8,67194	-77,35722	Cabo Tiburon	HG0176	VERDADERO
a550962-4383627-121220-367_A01.CEL	RG Ap1	A. palmata	Tayrona	11,32222	-74,16666	Concha	HG0168	VERDADERO
a550962-4368120-060520-251_A07.CEL	RG Ap10	A. palmata	Tayrona	11,32	-74,20083	I. Aguja	HG0238	VERDADERO
a550962-4383627-121220-367_A15.CEL	RG Ap14	A. palmata	Tayrona	11,31749	-74,13393	Chengue	HG0239	VERDADERO
a550962-4383627-121220-367_A17.CEL	RG Ap15	A. palmata	Tayrona	11,31749	-74,13393	Chengue	HG0138	VERDADERO
a550962-4368120-060520-251_A13.CEL	RG Ap17	A. palmata	Tayrona	11,31749	-74,13393	Chengue	HG0234	VERDADERO
a550962-4368120-060520-251_A15.CEL	RG Ap18	A. palmata	Tayrona	11,31749	-74,13393	Chengue	HG0234	VERDADERO
a550962-4368120-060520-251_A17.CEL	RG Ap19	A. palmata	Tayrona	11,32319	-74,11334	Gayraca	HG0161	VERDADERO
a550962-4383627-121220-367_A21.CEL	RG Ap20	A. palmata	Tayrona	11,32319	-74,11334	Gayraca	HG0147	VERDADERO
a550962-4368120-060520-251_A19.CEL	RG Ap21	A. palmata	Tayrona	11,32319	-74,11334	Gayraca	HG0220	VERDADERO
a550962-4383627-121220-367_A23.CEL	RG Ap22	A. palmata	Tayrona	11,32319	-74,11334	Gayraca	HG0162	VERDADERO
a550962-4368120-060520-251_A21.CEL	RG Ap25	A. palmata	Tayrona	11,33172	-74,05947	Cinto	HG0152	VERDADERO
a550962-4383627-121220-367_C07.CEL	RG Ap28	A. palmata	Tayrona	11,3108	-74,19032	Aguja	HG0246	VERDADERO
a550962-4383627-121220-367_C09.CEL	RG Ap29	A. palmata	Tayrona	11,3108	-74,19032	Aguja	HG0171	VERDADERO
a550962-4383627-121220-367_A03.CEL	RG Ap3	A. palmata	Tayrona	11,32222	-74,16666	Concha	HG0242	VERDADERO
a550962-4383627-121220-367_C11.CEL	RG Ap30	A. palmata	Tayrona	11,3108	-74,19032	Aguja	HG0261	VERDADERO
a550962-4368120-060520-251_C01.CEL	RG Ap31	A. palmata	Tayrona	11,3108	-74,19032	Aguja	HG0248	VERDADERO
a550962-4368120-060520-251_C03.CEL	RG Ap32	A. palmata	Tayrona	11,3108	-74,19032	Aguja	HG0151	VERDADERO
a550962-4383627-121220-367_C13.CEL	RG Ap33	A. palmata	Cartagena	10,185	-75,74556	Punta Brava	HG0237	VERDADERO
a550962-4383627-121220-367_C15.CEL	RG Ap34	A. palmata	Cartagena	10,185	-75,74556	Punta Brava	HG0235	VERDADERO
a550962-4368120-060520-251_C05.CEL	RG Ap35	A. palmata	Cartagena	10,185	-75,74556	Punta Brava	HG0172	VERDADERO
a550962-4383627-121220-367_C17.CEL	RG Ap36	A. palmata	Rosario	10,18553	-75,7279	I. Fiesta	HG0219	VERDADERO
a550962-4368120-060520-251_C07.CEL	RG Ap37	A. palmata	Rosario	10,18553	-75,7279	I. Fiesta	HG0153	VERDADERO
a550962-4383627-121220-367_C19.CEL	RG Ap38	A. palmata	Rosario	10,18553	-75,7279	I. Fiesta	HG0197	VERDADERO
a550962-4368120-060520-251_C09.CEL	RG Ap39	A. palmata	Rosario	10,18553	-75,7279	I. Fiesta	HG0153	VERDADERO
a550962-4383627-121220-367_A05.CEL	RG Ap4	A. palmata	Tayrona	11,32222	-74,16666	Concha	HG0221	VERDADERO
a550962-4383627-121220-367_C21.CEL	RG Ap40	A. palmata	Rosario	10,16262	-75,79881	Rosario	HG0254	VERDADERO
a550962-4368120-060520-251_C11.CEL	RG Ap41	A. palmata	Rosario	10,16262	-75,79881	Rosario	HG0137	VERDADERO
a550962-4383627-121220-367_C23.CEL	RG Ap42	A. palmata	San Bernardo	9,7613	-75,87303	Maravilla	HG0192	VERDADERO
a550962-4368120-060520-251_C13.CEL	RG Ap43	A. palmata	San Bernardo	9,7613	-75,87303	Maravilla	HG0214	VERDADERO
a550962-4368120-060520-251_C15.CEL	RG Ap44	A. palmata	San Bernardo	9,7613	-75,87303	Maravilla	HG0232	VERDADERO
a550962-4383627-121220-367_E03.CEL	RG Ap46	A. palmata	San Bernardo	9,81472	-75,85583	Bajo Hojuela	HG0211	VERDADERO
a550962-4368120-060520-251_C17.CEL	RG Ap47	A. palmata	San Bernardo	9,81472	-75,85583	Bajo Hojuela	HG0263	VERDADERO
a550962-4368120-060520-251_C19.CEL	RG Ap48	A. palmata	San Bernardo	9,81472	-75,85583	Bajo Hojuela	HG0211	VERDADERO

a550962-4383627-121220-367_E05.CEL	RG Ap49	A. palmata	San Bernardo	9,81472	-75,85583	Bajo Hojuela	HG0154	VERDADERO
a550962-4368120-060520-251_A03.CEL	RG Ap5	A. palmata	Tayrona	11,32222	-74,16666	Concha	HG0241	VERDADERO
a550962-4383627-121220-367_E07.CEL	RG Ap50	A. palmata	San Bernardo	9,81472	-75,85583	Bajo Hojuela	HG0211	VERDADERO
a550962-4383627-121220-367_E09.CEL	RG Ap51	A. palmata	San Bernardo	9,81472	-75,85583	Bajo Hojuela	HG0211	VERDADERO
a550962-4383627-121220-367_E11.CEL	RG Ap52	A. palmata	San Bernardo	9,81472	-75,85583	Bajo Hojuela	HG0154	VERDADERO
a550962-4368120-060520-251_C21.CEL	RG Ap53	A. palmata	San Bernardo	9,81472	-75,85583	Bajo Hojuela	HG0154	VERDADERO
a550962-4368120-060520-251_C23.CEL	RG Ap54	A. palmata	San Bernardo	9,81472	-75,85583	Bajo Hojuela	HG0263	VERDADERO
a550962-4383627-121220-367_E13.CEL	RG Ap55	A. palmata	San Bernardo	9,81472	-75,85583	Bajo Hojuela	HG0211	VERDADERO
a550962-4368120-060520-251_E01.CEL	RG Ap57	A. palmata	San Bernardo	9,76111	-75,87277	Maravilla	HG0167	VERDADERO
a550962-4368120-060520-251_E03.CEL	RG Ap58	A. palmata	San Bernardo	9,76111	-75,87277	Maravilla	HG0167	VERDADERO
a550962-4383627-121220-367_E17.CEL	RG Ap59	A. palmata	San Bernardo	9,76111	-75,87277	Maravilla	HG0262	VERDADERO
a550962-4368120-060520-251_A05.CEL	RG Ap6	A. palmata	Tayrona	11,32	-74,20083	I. Aguja	HG0231	VERDADERO
a550962-4383627-121220-367_E19.CEL	RG Ap60	A. palmata	San Bernardo	9,70666	-75,85111	Ceicen	HG0262	VERDADERO
a550962-4383627-121220-367_E21.CEL	RG Ap61	A. palmata	San Bernardo	9,70666	-75,85111	Ceicen	HG0212	VERDADERO
a550962-4368120-060520-251_E05.CEL	RG Ap62	A. palmata	San Bernardo	9,70666	-75,85111	Ceicen	HG0212	VERDADERO
a550962-4383627-121220-367_E23.CEL	RG Ap63	A. palmata	San Bernardo	9,70666	-75,85111	Ceicen	HG0212	VERDADERO
a550962-4368120-060520-251_E07.CEL	RG Ap64	A. palmata	San Bernardo	9,70666	-75,85111	Ceicen	HG0167	VERDADERO
a550962-4368120-060520-251_E09.CEL	RG Ap65	A. palmata	San Bernardo	9,80444	-75,82111	Batea	HG0140	VERDADERO
a550962-4368120-060520-251_E11.CEL	RG Ap66	A. palmata	San Bernardo	9,80808	-75,83299	Llantas	HG0226	VERDADERO
a550962-4383627-121220-367_G01.CEL	RG Ap67	A. palmata	Capurganá	8,64472	-77,33972	Bajo Naui	HG0196	VERDADERO
a550962-4383627-121220-367_G03.CEL	RG Ap68	A. palmata	Capurganá	8,64472	-77,33972	Bajo Naui	HG0233	VERDADERO
a550962-4368120-060520-251_E13.CEL	RG Ap69	A. palmata	Capurganá	8,64472	-77,33972	Bajo Naui	HG0225	VERDADERO
a550962-4383627-121220-367_A07.CEL	RG Ap7	A. palmata	Tayrona	11,32	-74,20083	I. Aguja	HG0166	VERDADERO
a550962-4383627-121220-367_G05.CEL	RG Ap70	A. palmata	Capurganá	8,64472	-77,33972	Bajo Naui	HG0217	VERDADERO
a550962-4368120-060520-251_E15.CEL	RG Ap71	A. palmata	Capurganá	8,64472	-77,33972	Bajo Naui	HG0150	VERDADERO
a550962-4383627-121220-367_G07.CEL	RG Ap72	A. palmata	Capurganá	8,67111	-77,35805	Cabo Tiburon	HG0213	VERDADERO
a550962-4368120-060520-251_E17.CEL	RG Ap73	A. palmata	Capurganá	8,67111	-77,35805	Cabo Tiburon	HG0230	VERDADERO
a550962-4383627-121220-367_G09.CEL	RG Ap74	A. palmata	Capurganá	8,67111	-77,35805	Cabo Tiburon	HG0224	VERDADERO
a550962-4368120-060520-251_E19.CEL	RG Ap75	A. palmata	Capurganá	8,67111	-77,35805	Cabo Tiburon	HG0170	VERDADERO
a550962-4368120-060520-251_E21.CEL	RG Ap76	A. palmata	Capurganá	8,65278	-77,34556	Coquera	HG0218	VERDADERO
a550962-4383627-121220-367_G11.CEL	RG Ap77	A. palmata	Capurganá	8,65278	-77,34556	Coquera	HG0210	VERDADERO
a550962-4368120-060520-251_E23.CEL	RG Ap78	A. palmata	Capurganá	8,65278	-77,34556	Coquera	HG0251	VERDADERO
a550962-4368120-060520-251_G01.CEL	RG Ap79	A. palmata	Capurganá	8,64472	-77,33972	Bajo Naui	HG0250	VERDADERO
a550962-4383627-121220-367_A09.CEL	RG Ap8	A. palmata	Tayrona	11,32	-74,20083	I. Aguja	HG0156	VERDADERO
a550962-4383627-121220-367_G13.CEL	RG Ap80	A. palmata	Capurganá	8,64472	-77,33972	Bajo Naui	HG0163	VERDADERO
a550962-4368120-060520-251_G03.CEL	RG Ap81	A. palmata	Capurganá	8,64472	-77,33972	Bajo Naui	HG0247	VERDADERO

a550962-4383627-121220-367_G15.CEL	RG Ap82	A. palmata	Capurganá	8,64472	-77,33972	Bajo Naui	HG0236	VERDADERO
a550962-4383627-121220-367_G17.CEL	RG Ap83	A. palmata	Capurganá	8,64472	-77,33972	Bajo Naui	HG0165	VERDADERO
a550962-4383627-121220-367_G19.CEL	RG Ap84	A. palmata	Capurganá	8,64472	-77,33972	Bajo Naui	HG0148	VERDADERO
a550962-4368120-060520-251_G05.CEL	RG Ap85	A. palmata	Capurganá	8,64472	-77,33972	Bajo Naui	HG0194	VERDADERO
a550962-4368120-060520-251_G07.CEL	RG Ap86	A. palmata	Capurganá	8,64472	-77,33972	Bajo Naui	HG0252	VERDADERO
a550962-4383627-121220-367_G21.CEL	RG Ap87	A. palmata	San Andrés	14,3625	-80,16138	Serrana	HG0139	VERDADERO
a550962-4383627-121220-367_G23.CEL	RG Ap89	A. palmata	San Andrés	13,565	-80,04055	Roncador	HG0195	VERDADERO
a550962-4383627-121220-367_A11.CEL	RG Ap9	A. palmata	Tayrona	11,32	-74,20083	I. Aguja	HG0171	VERDADERO
a550962-4383627-121220-367_I01.CEL	RG Ap90	A. palmata	San Andrés	14,3625	-80,16138	Serrana	HG0164	VERDADERO
a550962-4368120-060520-251_G11.CEL	RG Ap91	A. palmata	San Andrés	14,3625	-80,16138	Serrana	HG0216	VERDADERO
a550962-4383627-121220-367_I03.CEL	RG Ap92	A. palmata	San Andrés	13,565	-80,04055	Roncador	HG0193	VERDADERO
a550962-4383627-121220-367_I05.CEL	RG Ap94	A. palmata	I. Arena	11,23472	-75,60111	I. Arena	HG0169	VERDADERO
a550962-4368120-060520-251_G15.CEL	RG Ap95	A. palmata	I. Arena	11,23472	-75,60111	I. Arena	HG0160	VERDADERO
a550962-4368120-060520-251_G17.CEL	RG Ap96	A. palmata	I. Arena	11,23472	-75,60111	I. Arena	HG0149	VERDADERO
a550962-4368120-060520-251_G19.CEL	RG Ap97	A. palmata	I. Arena	11,23472	-75,60111	I. Arena	HG0215	VERDADERO
a550962-4368120-060520-251_I07.CEL	RG Ac10	A. cervicornis	Tayrona	11,33625	-74,05276	Cinto	HG0111	VERDADERO
a550962-4383627-121220-367_I13.CEL	RG Ac11	A. cervicornis	Rosario	10,18555	-75,73805	I. Fiesta	HG0044	VERDADERO
a550962-4383627-121220-367_I15.CEL	RG Ac12	A. cervicornis	Rosario	10,18444	-75,73194	I. Grande	HG0047	VERDADERO
a550962-4368120-060520-251_I09.CEL	RG Ac13	A. cervicornis	Rosario	10,18444	-75,73194	I. Grande	HG0069	VERDADERO
a550962-4383627-121220-367_I17.CEL	RG Ac14	A. cervicornis	Rosario	10,17194	-75,75527	Caribaru	HG0073	VERDADERO
a550962-4368120-060520-251_I11.CEL	RG Ac16	A. cervicornis	Rosario	10,17194	-75,75527	Caribaru	HG0068	VERDADERO
a550962-4368120-060520-251_I13.CEL	RG Ac17	A. cervicornis	Rosario	10,17194	-75,75527	Caribaru	HG0023	VERDADERO
a550962-4383627-121220-367_I21.CEL	RG Ac18	A. cervicornis	Rosario	10,16962	-75,75046	Luis Guerra	HG0097	VERDADERO
a550962-4368120-060520-251_I15.CEL	RG Ac19	A. cervicornis	Rosario	10,16962	-75,75046	Luis Guerra	HG0116	VERDADERO
a550962-4383627-121220-367_I23.CEL	RG Ac20	A. cervicornis	Rosario	10,16962	-75,75046	Luis Guerra	HG0127	VERDADERO
a550962-4368120-060520-251_I17.CEL	RG Ac21	A. cervicornis	Rosario	10,16962	-75,75046	Luis Guerra	HG0017	VERDADERO
a550962-4383627-121220-367_K01.CEL	RG Ac22	A. cervicornis	Rosario	10,16962	-75,75046	Luis Guerra	HG0064	VERDADERO
a550962-4368120-060520-251_I21.CEL	RG Ac24	A. cervicornis	Rosario	10,17306	-75,76778	Pavitos	HG0114	VERDADERO
a550962-4383627-121220-367_K03.CEL	RG Ac25	A. cervicornis	Rosario	10,17306	-75,76778	Pavitos	HG0114	VERDADERO
a550962-4368120-060520-251_I23.CEL	RG Ac26	A. cervicornis	Rosario	10,17306	-75,76778	Pavitos	HG0018	VERDADERO
a550962-4383627-121220-367_K07.CEL	RG Ac28	A. cervicornis	San Bernardo	9,70666	-75,85111	Ceicen	HG0046	VERDADERO
a550962-4368120-060520-251_K01.CEL	RG Ac29	A. cervicornis	San Bernardo	9,70666	-75,85111	Ceicen	HG0046	VERDADERO
a550962-4368120-060520-251_K03.CEL	RG Ac30	A. cervicornis	San Bernardo	9,70666	-75,85111	Ceicen	HG0046	VERDADERO
a550962-4368120-060520-251_K05.CEL	RG Ac31	A. cervicornis	San Bernardo	9,80222	-75,8194	Batea	HG0062	VERDADERO
a550962-4383627-121220-367_K09.CEL	RG Ac32	A. cervicornis	San Bernardo	9,80222	-75,8194	Batea	HG0039	VERDADERO
a550962-4368120-060520-251_K07.CEL	RG Ac33	A. cervicornis	San Bernardo	9,81472	-75,85583	Bajo Hojuela	HG0074	VERDADERO

a550962-4383627-121220-367_K11.CEL	RG Ac34	A. cervicornis	San Bernardo	9,81472	-75,85583	Bajo Hojuela	HG0097	VERDADERO
a550962-4368120-060520-251_K09.CEL	RG Ac35	A. cervicornis	San Bernardo	9,81472	-75,85583	Bajo Hojuela	HG0010	VERDADERO
a550962-4383627-121220-367_K13.CEL	RG Ac36	A. cervicornis	San Bernardo	9,81472	-75,85583	Bajo Hojuela	HG0097	VERDADERO
a550962-4383627-121220-367_K15.CEL	RG Ac37	A. cervicornis	San Bernardo	9,75014	-75,86858	De los Santos	HG0054	VERDADERO
a550962-4368120-060520-251_K11.CEL	RG Ac38	A. cervicornis	San Bernardo	9,75014	-75,86858	De los Santos	HG0028	VERDADERO
a550962-4383627-121220-367_K17.CEL	RG Ac39	A. cervicornis	San Bernardo	9,75014	-75,86858	De los Santos	HG0028	VERDADERO
a550962-4368120-060520-251_I01.CEL	RG Ac4	A. cervicornis	Tayrona	11,3213	-74,12826	Chengue	HG0117	VERDADERO
a550962-4383627-121220-367_K19.CEL	RG Ac40	A. cervicornis	San Bernardo	9,75014	-75,86858	De los Santos	HG0054	VERDADERO
a550962-4368120-060520-251_K13.CEL	RG Ac41	A. cervicornis	San Bernardo	9,75014	-75,86858	De los Santos	HG0013	VERDADERO
a550962-4368120-060520-251_K15.CEL	RG Ac42	A. cervicornis	San Bernardo	9,80244	-75,81946	La Pared	HG0040	VERDADERO
a550962-4383627-121220-367_K23.CEL	RG Ac44	A. cervicornis	San Bernardo	9,80244	-75,81946	La Pared	HG0042	VERDADERO
a550962-4383627-121220-367_M01.CEL	RG Ac45	A. cervicornis	San Bernardo	9,80244	-75,81946	La Pared	HG0039	VERDADERO
a550962-4368120-060520-251_K17.CEL	RG Ac46	A. cervicornis	San Bernardo	9,80244	-75,81946	La Pared	HG0041	VERDADERO
a550962-4368120-060520-251_K19.CEL	RG Ac47	A. cervicornis	Capurganá	8,64472	-77,33972	Bajo Naui	HG0012	VERDADERO
a550962-4368120-060520-251_K21.CEL	RG Ac49	A. cervicornis	Capurganá	8,64472	-77,33972	Bajo Naui	HG0019	VERDADERO
a550962-4368120-060520-251_K23.CEL	RG Ac50	A. cervicornis	Capurganá	8,64472	-77,33972	Bajo Naui	HG0072	VERDADERO
a550962-4368120-060520-251_M01.CEL	RG Ac51	A. cervicornis	Capurganá	8,64017	-77,32317	Golfo de Uraba	HG0025	VERDADERO
a550962-4383627-121220-367_M05.CEL	RG Ac52	A. cervicornis	Capurganá	8,64017	-77,32317	Golfo de Uraba	HG0025	VERDADERO
a550962-4383627-121220-367_M07.CEL	RG Ac53	A. cervicornis	Capurganá	8,64017	-77,32317	Golfo de Uraba	HG0025	VERDADERO
a550962-4383627-121220-367_M09.CEL	RG Ac54	A. cervicornis	Capurganá	8,67194	-77,35722	Cabo Tiburon	HG0027	VERDADERO
a550962-4368120-060520-251_M03.CEL	RG Ac55	A. cervicornis	Capurganá	8,67194	-77,35722	Cabo Tiburon	HG0016	VERDADERO
a550962-4368120-060520-251_M05.CEL	RG Ac56	A. cervicornis	Capurganá	8,67194	-77,35722	Cabo Tiburon	HG0029	VERDADERO
a550962-4383627-121220-367_M13.CEL	RG Ac58	A. cervicornis	San Andrés	13,50027	-80,03194	Roncador	HG0075	VERDADERO
a550962-4368120-060520-251_M07.CEL	RG Ac59	A. prolifera	San Andrés	13,50027	-80,03194	Roncador	HG0118	VERDADERO
a550962-4383627-121220-367_I09.CEL	RG Ac6	A. cervicornis	Tayrona	11,3213	-74,12826	Chengue	HG0045	VERDADERO
a550962-4368120-060520-251_M09.CEL	RG Ac60	A. cervicornis	San Andrés	13,50027	-80,03194	Roncador	HG0024	VERDADERO
a550962-4383627-121220-367_M15.CEL	RG Ac61	A. cervicornis	San Andrés	14,24861	-81,23861	Quita Sueño	HG0022	VERDADERO
a550962-4368120-060520-251_M11.CEL	RG Ac62	A. cervicornis	San Andrés	14,24861	-81,23861	Quita Sueño	HG0061	VERDADERO
a550962-4383627-121220-367_M17.CEL	RG Ac63	A. cervicornis	San Andrés	14,24861	-81,23861	Quita Sueño	HG0096	VERDADERO
a550962-4368120-060520-251_M13.CEL	RG Ac64	A. cervicornis	San Andrés	14,24861	-81,23861	Quita Sueño	HG0014	VERDADERO
a550962-4368120-060520-251_M15.CEL	RG Ac65	A. cervicornis	San Andrés	13,37777	-81,38666	Providencia	HG0015	VERDADERO
a550962-4368120-060520-251_M17.CEL	RG Ac66	A. cervicornis	San Andrés	13,37777	-81,38666	Providencia	HG0021	VERDADERO
a550962-4383627-121220-367_M19.CEL	RG Ac67	A. cervicornis	San Andrés	13,37777	-81,38666	Providencia	HG0082	VERDADERO
a550962-4368120-060520-251_M19.CEL	RG Ac68	A. cervicornis	San Andrés	12,59528	-81,71111	West Point	HG0112	VERDADERO
a550962-4383627-121220-367_M21.CEL	RG Ac69	A. cervicornis	San Andrés	12,59528	-81,71111	West Point	HG0113	VERDADERO
a550962-4368120-060520-251_I03.CEL	RG Ac7	A. cervicornis	Tayrona	11,3213	-74,12826	Chengue	HG0110	VERDADERO

a550962-4383627-121220-367_M23.CEL	RG Ac70	A. cervicornis	San Andrés	12,59528	-81,71111	West Point	HG0071	VERDADERO
a550962-4368120-060520-251_M21.CEL	RG Ac71	A. cervicornis	San Andrés	12,59528	-81,71111	Cayo Bolivar	HG0020	VERDADERO
a550962-4383627-121220-367_O01.CEL	RG Ac73	A. cervicornis	San Andrés	12,59528	-81,71111	Plaza de Toros	HG0083	VERDADERO
a550962-4383627-121220-367_O03.CEL	RG Ac74	A. cervicornis	San Andrés	12,59528	-81,71111	Plaza de Toros	HG0084	VERDADERO
a550962-4368120-060520-251_O01.CEL	RG Ac75	A. cervicornis	San Andrés	12,59528	-81,71111	Plaza de Toros	HG0092	VERDADERO
a550962-4368120-060520-251_O03.CEL	RG Ac76	A. cervicornis	San Andrés	14,3625	-80,16138	Serrana	HG0070	VERDADERO
a550962-4383627-121220-367_O05.CEL	RG Ac77	A. cervicornis	San Andrés	14,3625	-80,16138	Serrana	HG0076	VERDADERO
a550962-4368120-060520-251_O05.CEL	RG Ac78	A. cervicornis	San Andrés	14,3625	-80,16138	Serrana	HG0095	VERDADERO
a550962-4383627-121220-367_O07.CEL	RG Ac79	A. cervicornis	San Andrés	14,3625	-80,16138	Serrana	HG0048	VERDADERO
a550962-4368120-060520-251_I05.CEL	RG Ac8	A. cervicornis	Tayrona	11,33625	-74,05276	Cinto	HG0111	VERDADERO
a550962-4383627-121220-367_O09.CEL	RG Ac82	A. cervicornis	I. Arena	10,73864	-75,3498	I. Arena	HG0038	VERDADERO
a550962-4368120-060520-251_O11.CEL	RG Ac83	A. cervicornis	I. Arena	10,73864	-75,3498	I. Arena	HG0038	VERDADERO
a550962-4368120-060520-251_O13.CEL	RG Ac84	A. cervicornis	I. Arena	10,73864	-75,3498	I. Arena	HG0038	VERDADERO
a550962-4368120-060520-251_O19.CEL	RG Ac87	A. prolifera	I. Arena	10,73706	-75,35616	Hibrido 3	HG0119	VERDADERO
NA	RG Ac1	A. cervicornis	Tayrona	11,326494	-74,07825	Neguanje	failed	VERDADERO
NA	RG Ac15	A. cervicornis	Rosario	10,17194	-75,75527	Caribaru	failed	VERDADERO
NA	RG Ac2	A. cervicornis	Tayrona	11,32649	-74,07825	Neguanje	failed	VERDADERO
NA	RG Ac23	A. cervicornis	Cartagena	10,24831	-75,62425	Cartagena	failed	VERDADERO
NA	RG Ac27	A. cervicornis	San Bernardo	9,70666	-75,85111	Ceicen	failed	VERDADERO
NA	RG Ac3	A. cervicornis	Tayrona	11,326494	-74,07825	Neguanje	failed	VERDADERO
NA	RG Ac48	A. cervicornis	Capurganá	8,64472	-77,33972	Bajo Naui	failed	VERDADERO
NA	RG Ac5	A. cervicornis	Tayrona	11,3213	-74,12826	Chengue	failed	VERDADERO
NA	RG Ac72	A. cervicornis	San Andrés	12,59528	-81,71111	Plaza de Toros	failed	VERDADERO
NA	RG Ac80	A. cervicornis	I. Fuerte	9,36818	-76,20421	I. Fuerte	failed	VERDADERO
NA	RG Ac81	A. cervicornis	I. Fuerte	9,36818	-76,20421	I. Fuerte	failed	VERDADERO
NA	RG Ac85	A. cervicornis	I. Arena	10,73706	-75,35616	Hibrido 1	failed	VERDADERO
NA	RG Ac86	A. cervicornis	I. Arena	10,73706	-75,35616	Hibrido 2	failed	VERDADERO
NA	RG Ac88	A. cervicornis	I. Arena	10,73706	-75,35616	Hibrido 4	failed	VERDADERO
NA	RG Ac89	A. cervicornis	I. Arena	10,73706	-75,35616	Hibrido 5	failed	VERDADERO
NA	RG Ac9	A. cervicornis	Tayrona	11,33625	-74,05276	Cinto	failed	VERDADERO
NA	RG Ap11	A. palmata	Tayrona	11,32097	-74,07801	Neguanje	failed	VERDADERO
NA	RG Ap12	A. palmata	Tayrona	11,32097	-74,07801	Neguanje	failed	VERDADERO
NA	RG Ap13	A. palmata	Tayrona	11,32097	-74,07801	Neguanje	failed	VERDADERO
NA	RG Ap16	A. palmata	Tayrona	11,31749	-74,13393	Chengue	failed	VERDADERO
NA	RG Ap23	A. palmata	Tayrona	11,33172	-74,05947	Cinto	failed	VERDADERO
NA	RG Ap24	A. palmata	Tayrona	11,33172	-74,05947	Cinto	failed	VERDADERO

NA	RG Ap26	A. palmata	Tayrona	11,33172	-74,05947	Cinto	failed	VERDADERO
NA	RG Ap27	A. palmata	Tayrona	11,33172	-74,05947	Cinto	failed	VERDADERO
NA	RG Ap45	A. palmata	San Bernardo	9,7613	-75,87303	Maravilla	failed	VERDADERO
NA	RG Ap56	A. palmata	San Bernardo	9,76111	-75,87277	Maravilla	failed	VERDADERO
NA	RG Ap88	A. palmata	San Andrés	14,3625	-80,16138	Serrana	failed	VERDADERO
NA	RG Ap93	A. palmata	San Andrés	13,565	-80,04055	Roncador	failed	VERDADERO
a550962-4368120-060520-253_G01.CEL	15918	A. cervicornis	Puerto Rico	18,004669	-67,33082	Margarita West Nursery	HG0210	FALSO
a550962-4368120-060520-253_G21.CEL	P199xC182_4	A. cervicornis	Belize	16,436717	-88,199017	LaughingBirdCaye	HG0207	FALSO
a550962-4368120-060520-253_I23.CEL	6298	A. cervicornis	Florida	24,517654	-81,62134	LowerKeys	HG0208	FALSO
a550962-4368120-060520-253_K23.CEL	6299	A. cervicornis	Florida	24,517654	-81,62134	LowerKeys	HG0206	FALSO
a550962-4368120-060520-253_O23.CEL	6303	A. cervicornis	Florida	24,517654	-81,62134	LowerKeys	HG0209	FALSO
a550962-4368120-060520-252_C07.CEL	4917	A. cervicornis	Florida	24,946333	-80,470166	Little Conch	HG0138	FALSO
a550962-4368120-060520-252_C09.CEL	4918	A. cervicornis	Florida	24,9509	-80,493617	Plantation Key	HG0149	FALSO
a550962-4368120-060520-252_E01.CEL	9395	A. cervicornis	Florida	24,517654	-81,62134	LowerKeys	HG0152	FALSO
a550962-4368120-060520-252_E05.CEL	9592	A. cervicornis	Florida	25,37236	-80,17155	Biscayne National Park	HG0146	FALSO
a550962-4368120-060520-252_E07.CEL	9609	A. cervicornis	Florida	25,42616	-80,14175	Biscayne National Park	HG0148	FALSO
a550962-4368120-060520-252_E09.CEL	9749	A. cervicornis	Puerto Rico	17,949552	-66,727433	Margara	HG0151	FALSO
a550962-4368120-060520-252_E11.CEL	9759	A. cervicornis	Puerto Rico	17,949552	-66,727433	Margara	HG0150	FALSO
a550962-4368120-060520-252_G11.CEL	13756	A. cervicornis	USVI	18,316367	-64,98855	FlatKey	HG0144	FALSO
a550962-4368120-060520-252_I05.CEL	13923	A. cervicornis	Curacao	12,043117	-68,762433	East Point	HG0137	FALSO
a550962-4368120-060520-252_I13.CEL	13935	A. cervicornis	Curacao	12,109283	-68,954933	WaterFactory	HG0136	FALSO
a550962-4368120-060520-252_I19.CEL	15364	A. cervicornis	Belize	16,182848	-88,65966	site 12	HG0154	FALSO
a550962-4368120-060520-252_I21.CEL	15371	A. cervicornis	Belize	16,182848	-88,65966	Carne Nursery	HG0142	FALSO
a550962-4368120-060520-252_I23.CEL	15372	A. cervicornis	Belize	16,182848	-88,65966	dory channel	HG0143	FALSO
a550962-4368120-060520-252_K03.CEL	15375	A. cervicornis	Belize	16,182848	-88,65966	False Caye	HG0139	FALSO
a550962-4368120-060520-252_K05.CEL	15377	A. cervicornis	Belize	16,182848	-88,65966	by_RHT1	HG0145	FALSO
a550962-4368120-060520-252_K07.CEL	15378	A. cervicornis	Belize	16,182848	-88,65966	BBC_patch	HG0147	FALSO
a550962-4368120-060520-252_K13.CEL	15626	A. cervicornis	Belize	16,790939	-88,083006	Curlew	HG0135	FALSO
a550962-4368120-060520-252_M09.CEL	15616	A. cervicornis	Belize	16,182848	-88,65966	Glovers-118	HG0153	FALSO
a550962-4368120-060520-252_O01.CEL	15719	A. cervicornis	Belize	16,182848	-88,65966	Belize	HG0141	FALSO
a550962-4368120-060520-252_O21.CEL	15729	A. cervicornis	Cuba	23,220565	-82,346683	JudyLang	HG0140	FALSO
a550962-4368120-060520-256_A09.CEL	4936	A. cervicornis	Florida	24,812933	-80,669567	Lower Matecumbe	HG0026	FALSO
a550962-4368120-060520-256_A11.CEL	4955	A. cervicornis	Florida	25,13985	-80,294583	CRF	HG0042	FALSO
a550962-4368120-060520-256_A13.CEL	6649	A. cervicornis	Florida	24,692317	-80,97575	CRF	HG0002	FALSO
a550962-4368120-060520-256_A15.CEL	9719	A. cervicornis	Puerto Rico	17,949552	-66,727433	Margara	HG0030	FALSO
a550962-4368120-060520-256_A19.CEL	13716	A. cervicornis	USVI	18,405727	-64,90444	HansLollik	HG0006	FALSO

a550962-4368120-060520-256_A23.CEL	4915	A. cervicornis	Florida	24,9519	-80,451133	Conch Reef	HG0057		FALSO
a550962-4368120-060520-256_C07.CEL	4909	A. cervicornis	Florida	25,003583	-80,450917	Patch Reef	HG0037		FALSO
a550962-4368120-060520-256_C09.CEL	4943	A. cervicornis	Florida	24,80205	-80,667367	Lower Matecumbe	HG0047		FALSO
a550962-4368120-060520-256_C11.CEL	4956	A. cervicornis	Florida	25,0208	-80,40085	CRF	HG0054		FALSO
a550962-4368120-060520-256_C13.CEL	6653	A. cervicornis	Florida	24,692317	-80,97575	CRF	HG0028		FALSO
a550962-4368120-060520-256_E07.CEL	4911	A. cervicornis	Florida	25,00945	-80,4514	Patch Reef	HG0050		FALSO
a550962-4368120-060520-256_E09.CEL	4944	A. cervicornis	Florida	24,817933	-80,651767	CRF	HG0041		FALSO
a550962-4368120-060520-256_E11.CEL	4958	A. cervicornis	Florida	24,72015	-80,92875	Grassy Key	HG0043		FALSO
a550962-4368120-060520-256_E23.CEL	SIBM13_3	A. cervicornis	Florida	25,0179	-80,368617	Sandisland Blue x M13	HG0027		FALSO
a550962-4368120-060520-256_G05.CEL	3844	A. cervicornis	USVI	18,31822	-64,99104	Flat Cay	HG0003		FALSO
a550962-4368120-060520-256_G07.CEL	4913	A. cervicornis	Florida	24,9886	-80,402567	Pickles Reef	HG0044		FALSO
a550962-4368120-060520-256_G09.CEL	4945	A. cervicornis	Florida	24,80255	-80,6524	Lower Matecumbe	HG0014		FALSO
a550962-4368120-060520-256_G11.CEL	4963	A. cervicornis	Florida	24,692317	-80,97575	Grassy Key	HG0023		FALSO
a550962-4368120-060520-256_G23.CEL	SIBM13_4	A. cervicornis	Florida	25,0179	-80,368617	Sandisland Blue x M13	HG0051		FALSO
a550962-4368120-060520-256_I03.CEL	1372_Symbio	A. cervicornis	Florida	25,373333	-80,160217	Marker 3	HG0024		FALSO
a550962-4368120-060520-256_I07.CEL	4914	A. cervicornis	Florida	24,986167	-80,4171	Pickles Reef	HG0040		FALSO
a550962-4368120-060520-256_I09.CEL	4949	A. cervicornis	Florida	25,014683	-80,416917	CRF	HG0019		FALSO
a550962-4368120-060520-256_I11.CEL	4964	A. cervicornis	Florida	24,692317	-80,97575	Grassy Key	HG0016		FALSO
a550962-4368120-060520-256_I15.CEL	11409	A. cervicornis	Belize	17,833433	-87,992347	Coral Gardens	HG0039		FALSO
a550962-4368120-060520-256_I19.CEL	13829	A. cervicornis	Belize	16,88806	-87,75973	GloversAtoll	HG0029		FALSO
a550962-4368120-060520-256_K07.CEL	4919	A. cervicornis	Florida	24,951883	-80,491617	Patch Reef	HG0060		FALSO
a550962-4368120-060520-256_K09.CEL	4951	A. cervicornis	Florida	24,813617	-80,666967	Lower Matecumbe	HG0052		FALSO
a550962-4368120-060520-256_K13.CEL	9577	A. cervicornis	Florida	25,39894	-80,16032	Biscayne National Park	HG0015		FALSO
a550962-4368120-060520-256_K19.CEL	13839	A. cervicornis	Belize	16,88806	-87,75973	GloversAtoll	HG0059		FALSO
a550962-4368120-060520-256_K23.CEL	SIBM13_7	A. cervicornis	Florida	25,0179	-80,368617	Sandisland Blue x M13	HG0056		FALSO
a550962-4368120-060520-256_M05.CEL	4878	A. cervicornis	Florida	24,947083	-80,4659	Conch Reef	HG0007		FALSO
a550962-4368120-060520-256_M07.CEL	4922	A. cervicornis	Florida	25,013717	-80,415	Patch Reef	HG0038		FALSO
a550962-4368120-060520-256_M09.CEL	4952	A. cervicornis	Florida	24,821633	-80,65575	Lower Matecumbe	HG0058		FALSO
a550962-4368120-060520-256_M13.CEL	9607	A. cervicornis	Florida	25,42616	-80,14175	Biscayne National Park	HG0045		FALSO
a550962-4368120-060520-256_M23.CEL	SIBM13_8	A. cervicornis	Florida	25,0179	-80,368617	Sandisland Blue x M13	HG0031		FALSO
a550962-4368120-060520-256_O05.CEL	4897	A. cervicornis	Florida	25,00275	-80,422917	Molasses Reef	HG0018		FALSO
a550962-4368120-060520-256_O07.CEL	4932	A. cervicornis	Florida	24,832183	-80,640767	Lower Matecumbe	HG0017		FALSO
a550962-4368120-060520-256_O09.CEL	4953	A. cervicornis	Florida	24,9524	-80,451133	Conch Reef	HG0053		FALSO
a550962-4368120-060520-256_O13.CEL	9610	A. cervicornis	Florida	25,42616	-80,14175	Biscayne National Park	HG0013		FALSO
a550962-4393310-052921-062_A01.CEL	16065(441)	A. palmata	Curacao	12,064193	-68,853068	Spanish Waters	HG0432		FALSO
a550962-4368120-060520-253_A01.CEL	15915	A. palmata	Puerto Rico	18,004669	-67,33082	Margarita West Nursery	HG0262		FALSO

a550962-4368120-060520-253_A03.CEL	15629	A. palmata	Belize	16,80145	-88,0827	SouthCarrieBowCaye	HG0250	FALSO
a550962-4368120-060520-253_A05.CEL	15931	A. palmata	Puerto Rico	18,352618	-65,573963	Palomino Nursery	HG0221	FALSO
a550962-4368120-060520-253_A07.CEL	15939	A. palmata	Puerto Rico	17,9411	-66,8686	San Cristobal Nursery	HG0226	FALSO
a550962-4368120-060520-253_A11.CEL	15857	A. palmata	Puerto Rico	17,938	-66,8713	Cayo Coral East Nursery	HG0220	FALSO
a550962-4368120-060520-253_A15.CEL	15873	A. palmata	Puerto Rico	17,939048	-66,89222	Cayo Coral West Nursery	HG0223	FALSO
a550962-4368120-060520-253_A17.CEL	15884	A. palmata	Puerto Rico	17,939048	-66,89222	Cayo Coral West Nursery	HG0232	FALSO
a550962-4368120-060520-253_A19.CEL	15895	A. palmata	Puerto Rico	17,9243	-67,0999	Margarita East Nursery	HG0235	FALSO
a550962-4368120-060520-253_C07.CEL	15940	A. palmata	Puerto Rico	17,9411	-66,8686	San Cristobal Nursery	HG0225	FALSO
a550962-4368120-060520-253_C09.CEL	15948	A. palmata	Puerto Rico	17,94407	-67,07786	San Cristobal Nursery	HG0227	FALSO
a550962-4368120-060520-253_C11.CEL	15858	A. palmata	Puerto Rico	17,938	-66,8713	Cayo Coral East Nursery	HG0242	FALSO
a550962-4368120-060520-253_C15.CEL	15874	A. palmata	Puerto Rico	18,004669	-67,33082	Cayo Coral West Nursery	HG0260	FALSO
a550962-4368120-060520-253_C17.CEL	15885	A. palmata	Puerto Rico	17,939048	-66,89222	Cayo Coral West Nursery	HG0241	FALSO
a550962-4368120-060520-253_C19.CEL	15900	A. palmata	Puerto Rico	17,9243	-67,0999	Margarita East Nursery	HG0261	FALSO
a550962-4368120-060520-253_E01.CEL	15917	A. palmata	Puerto Rico	18,004669	-67,33082	Margarita West Nursery	HG0237	FALSO
a550962-4368120-060520-253_E07.CEL	15630	A. palmata	Belize	16,80145	-88,0827	SouthCarrieBowCaye	HG0222	FALSO
a550962-4368120-060520-253_E09.CEL	15949	A. palmata	Puerto Rico	17,9411	-66,8686	San Cristobal Nursery	HG0259	FALSO
a550962-4368120-060520-253_E13.CEL	15867	A. palmata	Puerto Rico	17,9411	-66,8686	Cayo Coral West Nursery	HG0228	FALSO
a550962-4368120-060520-253_E17.CEL	15887	A. palmata	Puerto Rico	17,9411	-66,8686	Cayo Coral West Nursery	HG0252	FALSO
a550962-4368120-060520-253_G09.CEL	15950	A. palmata	Puerto Rico	17,94407	-67,07786	San Cristobal Nursery	HG0249	FALSO
a550962-4368120-060520-253_G13.CEL	15868	A. palmata	Puerto Rico	18,004669	-67,33082	Cayo Coral West Nursery	HG0246	FALSO
a550962-4368120-060520-253_G17.CEL	15888	A. palmata	Puerto Rico	17,9411	-66,8686	Cayo Coral West Nursery	HG0239	FALSO
a550962-4368120-060520-253_G19.CEL	15905	A. palmata	Puerto Rico	17,9243	-67,0999	Margarita East Nursery	HG0258	FALSO
a550962-4368120-060520-253_I01.CEL	15627	A. palmata	Belize	16,80145	-88,0827	SouthCarrieBowCaye	HG0244	FALSO
a550962-4368120-060520-253_I05.CEL	15935	A. palmata	Puerto Rico	17,9411	-66,8686	San Cristobal Nursery	HG0256	FALSO
a550962-4368120-060520-253_I13.CEL	15869	A. palmata	Puerto Rico	17,9411	-66,8686	Cayo Coral West Nursery	HG0254	FALSO
a550962-4368120-060520-253_I15.CEL	15877	A. palmata	Puerto Rico	17,9411	-66,8686	Cayo Coral West Nursery	HG0253	FALSO
a550962-4368120-060520-253_I17.CEL	15891	A. palmata	Puerto Rico	17,9411	-66,8686	Cayo Coral West Nursery	HG0255	FALSO
a550962-4368120-060520-253_K05.CEL	15936	A. palmata	Puerto Rico	17,94407	-67,07786	San Cristobal Nursery	HG0230	FALSO
a550962-4368120-060520-253_K07.CEL	15944	A. palmata	Puerto Rico	17,9411	-66,8686	San Cristobal Nursery	HG0238	FALSO
a550962-4368120-060520-253_K13.CEL	15870	A. palmata	Puerto Rico	17,939048	-66,89222	Cayo Coral West Nursery	HG0240	FALSO
a550962-4368120-060520-253_M05.CEL	15937	A. palmata	Puerto Rico	17,9411	-66,8686	San Cristobal Nursery	HG0245	FALSO
a550962-4368120-060520-253_M07.CEL	15945	A. palmata	Puerto Rico	17,9411	-66,8686	San Cristobal Nursery	HG0248	FALSO
a550962-4368120-060520-253_M09.CEL	15953	A. palmata	Puerto Rico	17,9411	-66,8686	Gilligans Reef	HG0236	FALSO
a550962-4368120-060520-253_M13.CEL	15871	A. palmata	Puerto Rico	17,939048	-66,89222	Cayo Coral West Nursery	HG0251	FALSO
a550962-4368120-060520-253_M17.CEL	15893	A. palmata	Puerto Rico	17,9411	-66,8686	Cayo Coral West Nursery	HG0224	FALSO
a550962-4368120-060520-253_O05.CEL	15938	A. palmata	Puerto Rico	17,9411	-66,8686	San Cristobal Nursery	HG0257	FALSO

a550962-4368120-060520-253_O07.CEL	15946	A. palmata	Puerto Rico	17,9411	-66,8686	San Cristobal Nursery	HG0243	FALSO
a550962-4368120-060520-253_O09.CEL	15954	A. palmata	Puerto Rico	17,9411	-66,8686	Gilligans Reef	HG0247	FALSO
a550962-4368120-060520-253_O13.CEL	15872	A. palmata	Puerto Rico	17,9411	-66,8686	Cayo Coral West Nursery	HG0231	FALSO
a550962-4368120-060520-253_O17.CEL	15894	A. palmata	Puerto Rico	17,9411	-66,8686	Cayo Coral West Nursery	HG0229	FALSO
a550962-4368120-060520-252_A17.CEL	1151	A. palmata	Florida	24,456017	-81,859633	RockKey	HG0171	FALSO
a550962-4368120-060520-252_A23.CEL	2905	A. palmata	Florida	25,022308	-80,402375	KL4NOAA	HG0166	FALSO
a550962-4368120-060520-252_C11.CEL	5130	A. palmata	Puerto Rico	17,56493	-67,04515	San Cristobal	HG0177	FALSO
a550962-4368120-060520-252_C13.CEL	6898	A. palmata	Florida	24,54585	-81,405067	Looe Key	HG0186	FALSO
a550962-4368120-060520-252_E13.CEL	11954	A. palmata	Florida	24,710868	-80,947981	CRF	HG0201	FALSO
a550962-4368120-060520-252_E15.CEL	11956	A. palmata	Florida	24,710868	-80,947981	CRF	HG0197	FALSO
a550962-4368120-060520-252_E17.CEL	11955	A. palmata	Florida	24,710868	-80,947981	CRF	HG0204	FALSO
a550962-4368120-060520-252_E19.CEL	11957	A. palmata	Florida	24,710868	-80,947981	CRF	HG0174	FALSO
a550962-4368120-060520-252_E21.CEL	11958	A. palmata	Florida	24,710868	-80,947981	CRF	HG0205	FALSO
a550962-4368120-060520-252_E23.CEL	11962	A. palmata	Florida	24,710868	-80,947981	CRF	HG0185	FALSO
a550962-4368120-060520-252_G01.CEL	11963	A. palmata	Florida	24,710868	-80,947981	CRF	HG0202	FALSO
a550962-4368120-060520-252_G05.CEL	13694	A. palmata	USVI	18,357883	-65,032967	Botany2	HG0175	FALSO
a550962-4368120-060520-252_G07.CEL	13710	A. palmata	USVI	18,359033	-65,032833	Botany2	HG0165	FALSO
a550962-4368120-060520-252_G15.CEL	13805	A. palmata	Belize	16,772567	-88,1111	Sandbores2	HG0170	FALSO
a550962-4368120-060520-252_G17.CEL	13809	A. palmata	Belize	16,801322	-88,0825	SouthCarrieBowCay	HG0199	FALSO
a550962-4368120-060520-252_G19.CEL	13811	A. palmata	Belize	16,92005	-87,767217	GloversAtoll	HG0187	FALSO
a550962-4368120-060520-252_G21.CEL	13835	A. palmata	Belize	16,9084	-87,767217	GloversAtoll	HG0190	FALSO
a550962-4368120-060520-252_I01.CEL	13909	A. palmata	Curacao	12,083133	-68,895567	SeaAquarium2	HG0191	FALSO
a550962-4368120-060520-252_I03.CEL	13921	A. palmata	Curacao	12,0633	-68,859033	DirectorsBay	HG0173	FALSO
a550962-4368120-060520-252_I07.CEL	13927	A. palmata	Curacao	12,043367	-68,762517	EastPoint2	HG0193	FALSO
a550962-4368120-060520-252_I09.CEL	13929	A. palmata	Curacao	12,043467	-68,76315	EastPoint2	HG0176	FALSO
a550962-4368120-060520-252_I11.CEL	13931	A. palmata	Curacao	12,043317	-68,763533	EastPoint2	HG0194	FALSO
a550962-4368120-060520-252_I15.CEL	13937	A. palmata	Curacao	12,109517	-68,955367	WaterFactory2	HG0172	FALSO
a550962-4368120-060520-252_I17.CEL	13972	A. palmata	Curacao	12,109617	-68,954517	WaterFactory2	HG0168	FALSO
a550962-4368120-060520-252_K09.CEL	15391	A. palmata	Belize	16,182848	-88,65966	1D	HG0169	FALSO
a550962-4368120-060520-252_K11.CEL	15395	A. palmata	Belize	16,182848	-88,65966	CBC wypt 51	HG0192	FALSO
a550962-4368120-060520-252_K15.CEL	15397	A. palmata	Belize	16,182848	-88,65966	SWCB2	HG0178	FALSO
a550962-4368120-060520-252_K17.CEL	15398	A. palmata	Belize	16,182848	-88,65966	N of bath room	HG0198	FALSO
a550962-4368120-060520-252_K21.CEL	15610	A. palmata	Belize	16,801322	-88,0825	SouthCarrieBow	HG0189	FALSO
a550962-4368120-060520-252_K23.CEL	15611	A. palmata	Belize	16,801322	-88,0825	SouthCarrieBow	HG0196	FALSO
a550962-4368120-060520-252_M01.CEL	15612	A. palmata	Belize	16,801322	-88,0825	SouthCarrieBow	HG0195	FALSO
a550962-4368120-060520-252_M03.CEL	15613	A. palmata	Belize	16,80145	-88,0827	SouthCarrieBowCaye	HG0119	FALSO

a550962-4368120-060520-252_M15.CEL	15619	A. palmata	Belize	16,80145	-88,0827	SouthCarrieBowCaye	HG0188	FALSO
a550962-4368120-060520-252_M17.CEL	15620	A. palmata	Belize	16,80145	-88,0827	SouthCarrieBowCaye	HG0203	FALSO
a550962-4368120-060520-252_M21.CEL	15622	A. palmata	Belize	16,80145	-88,0827	SouthCarrieBowCaye	HG0200	FALSO
a550962-4368120-060520-252_O19.CEL	15720	A. palmata	Belize	16,182848	-88,65966	Belize	HG0167	FALSO
a550962-4368120-060520-256_A03.CEL	1127_Mixed	A. palmata	Florida	24,456017	-81,859633	RockKey	HG0079	FALSO
a550962-4368120-060520-256_A05.CEL	2724	A. palmata	Florida	25,01015	-80,37328	ML3	HG0123	FALSO
a550962-4368120-060520-256_A21.CEL	14391	A. palmata	Belize	17,268611	-87,816944	Carne Nursery	HG0127	FALSO
a550962-4368120-060520-256_C01.CEL	1087	A. palmata	Florida	24,479867	-81,718667	Western Sambo	HG0108	FALSO
a550962-4368120-060520-256_C03.CEL	1132_Symbio	A. palmata	Florida	24,456017	-81,859633	RockKey	HG0129	FALSO
a550962-4368120-060520-256_C05.CEL	2870	A. palmata	Florida	25,108917	-80,30525	GR1	HG0130	FALSO
a550962-4368120-060520-256_C15.CEL	11293	A. palmata	Belize	17,833433	-87,992347	Coral Gardens	HG0109	FALSO
a550962-4368120-060520-256_G03.CEL	1301	A. palmata	Florida	24,6209	-82,8675	DryTortugas2	HG0107	FALSO
a550962-4368120-060520-256_I01.CEL	1104	A. palmata	Florida	24,479867	-81,718667	Western Sambo	HG0120	FALSO
a550962-4368120-060520-256_I23.CEL	SIBM13_5	A. palmata	Florida	25,0179	-80,368617	Sandisland Blue x M13	HG0133	FALSO
a550962-4368120-060520-256_K01.CEL	1105	A. palmata	Florida	24,479867	-81,718667	Western Sambo	HG0124	FALSO
a550962-4368120-060520-256_K11.CEL	5735	A. palmata	Florida	25,00912	-80,37473	ML3	HG0080	FALSO
