# Supplementary material for: Fine scale population structure of Acropora palmata and Acropora cervicornis in the Colombian Caribbean
Source: PeerJ. 2022 Aug 30;10:e13854. doi: 10.7717/peerj.13854 (PMC9438773; doi:10.7717/peerj.13854)
Supplement: Supplemental Information 11 [file peerj-10-13854-s011.pdf]

ID	Affy probe id	STAGdb probe i	chrom	pos	Ordered Alle	pca_pvalue_p1	pca_qvalue	PCAdapt outlier	BAYES_fst	BAYES_qval	Bayescan outlier	CDS annotation
19104	AX-212310281	AP_var_5651	NW_015442071	25442	A // G	9,17E-06	0,000132416	outlier	0,028811	0,35001	n.s.	uncharacterized LOC107328026
19590	AX-212304636	Apop_10406	NW_015442651	15241	T // C	0,000160943	0,001633826	outlier	0,023925	0,52882	n.s.	uncharacterized LOC107330225%2C transcript variant X2
3382	AX-212301922	Apop_07709	NW_015441089	549133	C // G	6,00E-10	2,23E-08	outlier	0,066735	0,085132	n.s.	forkhead box protein D1-like
4108	AX-212315302	Apop_07801	NW_015441098	724132	T // C	2,99E-08	8,07E-07	outlier	0,058156	0,089135	n.s.	autophagy-related protein 13-like
4280	AX-212315353	Apop_07852	NW_015441104	88147	A // G	0,000147478	0,001555287	outlier	0,022563	0,6243	n.s.	DNA repair protein XRCC4-like
4316	AX-197963094	Apop_00503	NW_015441104	588950	C // G	1,66E-06	2,83E-05	outlier	0,025823	0,42056	n.s.	dnaJ homolog subfamily C member 22-like
5412	AX-212315462	Apop_07961	NW_015441117	837655	T // C	1,86E-07	3,92E-06	outlier	0,06413	0,060658	n.s.	uncharacterized LOC107334565
7698	AX-197981617	Apop_02675	NW_015441168	682199	A // C	0,000943292	0,006852142	outlier	0,022684	0,57447	n.s.	inactive tyrosine-protein kinase transmembrane receptor ROR1-like%2C transcript variant X1
11218	AX-197981819	Apop_02795	NW_015441279	25073	A // G	4,78E-09	1,49E-07	outlier	0,046834	0,13657	n.s.	uncharacterized LOC107344143
11736	AX-212303204	Apop_08974	NW_015441299	222088	A // G	0,000235594	0,002194387	outlier	0,023798	0,51716	n.s.	partitioning defective protein 3-like
10194	AX-212302940	Apop_08710	NW_015441239	300859	T // C	1,40E-06	2,48E-05	outlier	0,022997	0,54532	n.s.	alpha-methylacyl-CoA racemase-like
11796	AX-197981934	Apop_02864	NW_015441302	481804	T // C	0,000161754	0,001633826	outlier	0,024699	0,49033	n.s.	E3 ubiquitin-protein ligase TRIM71-like
14024	AX-212311399	Apop_03913	NW_015441389	418943	A // G	1,92E-09	6,45E-08	outlier	0,060843	0,086283	n.s.	adhesion G-protein coupled receptor D1-like%2C transcript variant X2
1900	AX-198038154	Afix_02306	NW_015441069	1289395	A // G	0,000313461	0,002771831	outlier	0,020644	0,88832	n.s.	neuronal acetylcholine receptor subunit alpha-10-like%2C transcript variant X1
15660	AX-212295654	AP_var_4310	NW_015441495	277602	T // C	0,001026071	0,007314654	outlier	0,022014	0,62856	n.s.	phosphatidylinositol 5-phosphate 4-kinase type-2 beta-like
15950	AX-197965548	Apop_01947	NW_015441509	318777	T // C	0,000226991	0,002129799	outlier	0,023179	0,57028	n.s.	tctex1 domain-containing protein 1-B-like
15970	AX-212317104	Apop_09586	NW_015441512	170081	A // C	0,000850421	0,006383432	outlier	0,021881	0,63803	n.s.	alpha-1%2C3-mannosyl-glycoprotein 4-beta-N-acetylglucosaminyltransferase B-like%2C transcript variant X1
18056	AX-212309845	AP_var_5215	NW_015441790	77818	T // G	0,001407201	0,009449458	outlier	0,021843	0,64871	n.s.	uncharacterized LOC107357143
2839	AX-197962772	Apop_00306	NW_015441079	308590	A // G	0,02114756	0,07045809	n.s.	0,11205	0,00074698	outlier	conserved oligomeric Golgi complex subunit 6-like
16871	AX-212317312	Apop_09794	NW_015441601	159196	T // G	0,05861034	0,1396204	n.s.	0,090585	0,0054459	outlier	leucine-rich repeat serine/threonine-protein kinase 2-like
9418	AX-212314058	Apop_06574	NW_015441211	553416	A // C	3,28E-10	1,37E-08	outlier	0,096728	0,0016572	outlier	DEP domain-containing mTOR-interacting protein-like%2C transcript variant X2
5414	AX-212302176	Apop_07963	NW_015441117	849244	C // G	6,38E-16	7,88E-14	outlier	0,090363	0,0044379	outlier	uncharacterized LOC107334569
6757	AX-197963538	Apop_00763	NW_015441148	825248	A // G	0,008348637	0,03678855	n.s.	0,072904	0,026237	outlier	uncharacterized GTP-binding protein C428.15-like
9893	AX-212293867	AP_var_2520	NW_015441225	4828	C // G	0,001354972	0,009229635	outlier	0,087624	0,0021668	outlier	helicase with zinc finger domain 2-like
6015	AX-197963396	Apop_00678	NW_015441134	167324	T // C	0,01257377	0,04997951	n.s.	0,072865	0,046726	outlier	ankyrin repeat domain-containing protein 11-like
12826	AX-197954642	Afix_10694	NW_015441352	30468	T // C	9,08E-12	5,35E-10	outlier	0,12237	0,00043244	outlier	prominin-1-A-like%2C transcript variant X4
7141	AX-212293135	AP_var_1785	NW_015441157	440741	A // C	0,000547005	0,004353482	outlier	0,11621	0	outlier	uncharacterized LOC107337495%2C transcript variant X1

14353	AX-198062528	Apop_01767	NW_015441414	116988	T // G	0,002283468	0,01398639	n.s.	0,082787	0,0086398	outlier	golgin subfamily B member 1-like
15211	AX-198062673	Apop_01855	NW_015441458	268008	A // T	0,00886776	0,03827198	n.s.	0,080422	0,013757	outlier	uncharacterized protein LOC107350536
9424	AX-212313978	Apop_06493	NW_015441211	573594	C // G	5,50E-11	2,63E-09	outlier	0,096424	0,0041239	outlier	uncharacterized LOC107340839
2834	AX-197942725	Afix_03183	NW_015441079	230465	C // G	1,63E-06	2,80E-05	outlier	0,10398	0,0063428	outlier	uncharacterized LOC107329078%2C transcript variant X1
3078	AX-212315134	Apop_07633	NW_015441082	399738	A // G	4,92E-07	9,66E-06	outlier	0,06103	0,10643	n.s.	uncharacterized LOC107331054
8974	AX-212299455	Apop_05256	NW_015441198	506150	T // C	0,000205326	0,001960158	outlier	0,024281	0,51225	n.s.	ficolin-2-like
11786	AX-198061918	Apop_01385	NW_015441302	474302	T // C	0,000161754	0,001633826	outlier	0,024961	0,45801	n.s.	E3 ubiquitin-protein ligase TRIM71-like
14356	AX-197965255	Apop_01769	NW_015441414	151827	T // C	0,001432063	0,009546159	outlier	0,020617	0,86789	n.s.	uncharacterized LOC107349285
9419	AX-197964053	Apop_01061	NW_015441211	559139	C // G	0,006712988	0,03130137	n.s.	0,096327	0,0019883	outlier	1-acyl-sn-glycerol-3-phosphate acyltransferase epsilon-like
5454	AX-198060637	Apop_00589	NW_015441119	309451	A // G	1,00E-06	1,83E-05	outlier	0,027295	0,44628	n.s.	transmembrane protein 145-like%2C transcript variant X3
11217	AX-197981817	Apop_02794	NW_015441279	24878	T // C	0,000917557	0,006729044	outlier	0,080077	0,0050953	outlier	uncharacterized LOC107344143
11216	AX-198078906	Apop_02792	NW_015441279	24492	T // C	3,18E-15	3,29E-13	outlier	0,076205	0,015204	outlier	uncharacterized protein LOC107344143
10244	AX-212307289	AP_var_2656	NW_015441241	463614	A // G	0,000921481	0,006744896	outlier	0,021056	0,88236	n.s.	uncharacterized LOC107342486
1646	AX-212301670	Apop_07457	NW_015441067	135850	A // G	0,000458997	0,003786891	outlier	0,020472	0,88927	n.s.	uncharacterized protein KIAA2026-like
12186	AX-212313246	Apop_05749	NW_015441314	365379	A // T	7,03E-06	0,000103178	outlier	0,068614	0,056356	n.s.	cell wall protein DAN4-like%2C transcript variant X2
13028	AX-197964984	Apop_01603	NW_015441361	133484	T // G	0,000486884	0,003982631	outlier	0,021459	0,80142	n.s.	cap-specific mRNA (nucleoside-2'-O-)-methyltransferase 1-like
1744	AX-198038047	Afix_02239	NW_015441068	851485	T // C	5,38E-05	0,000662296	outlier	0,033275	0,30913	n.s.	uncharacterized LOC107347779%2C transcript variant X4
13780	AX-197955661	Afix_11350	NW_015441378	132309	T // C	0,001040636	0,007363636	outlier	0,037634	0,2688	n.s.	uncharacterized LOC107348149
17204	AX-212317398	Apop_09880	NW_015441643	111668	T // C	0,000226991	0,002129799	outlier	0,022801	0,56625	n.s.	acid-sensing ion channel 2-like
17936	AX-197966028	Apop_02230	NW_015441751	139158	A // G	3,31E-08	8,85E-07	outlier	0,04883	0,12681	n.s.	fez family zinc finger protein 2-like
18663	AX-197966176	Apop_02310	NW_015441902	34007	T // C	0,001565966	0,01017789	n.s.	0,084142	0,023685	outlier	cytochrome P450 27C1-like
4922	AX-212315424	Apop_07923	NW_015441112	345084	A // G	1,13E-20	2,70E-18	outlier	0,11139	0,00030498	outlier	ATP-binding cassette sub-family B member 10%2C mitochondrial-like
11226	AX-198078935	Apop_02810	NW_015441279	32437	A // G	1,09E-16	1,61E-14	outlier	0,079967	0,0061609	outlier	uncharacterized LOC107344142
10099	AX-197964232	Apop_01167	NW_015441236	170492	T // C	0,02932777	0,0879871	n.s.	0,074257	0,032288	outlier	LOW QUALITY PROTEIN: E3 ubiquitin-protein ligase HERC2-like
10101	AX-197964235	Apop_01169	NW_015441236	176505	A // G	0,02932777	0,0879871	n.s.	0,075398	0,029096	outlier	LOW QUALITY PROTEIN: E3 ubiquitin-protein ligase HERC2-like
11471	AX-212303167	Apop_08937	NW_015441288	423802	T // C	0,0162789	0,05884639	n.s.	0,093918	0,0019883	outlier	uncharacterized protein LOC107344502 isoform X3
8902	AX-197981728	Apop_02739	NW_015441196	564510	A // G	8,45E-15	8,29E-13	outlier	0,11377	0,00013336	outlier	POU domain%2C class 4%2C transcription factor 3-like%2C transcript variant X1
12190	AX-212316552	Apop_09034	NW_015441314	370782	A // G	9,98E-07	1,83E-05	outlier	0,059971	0,080508	n.s.	#N/D
13302	AX-212294962	AP_var_3615	NW_015441365	413826	T // C	4,53E-08	1,16E-06	outlier	0,053072	0,12307	n.s.	#N/D

6770	AX-212302440	Apop_08210	NW_015441149	525123	C // G	6,92E-06	0,000101913	outlier	0,043946	0,1756	n.s.	#N/D
15859	AX-212298834	Apop_04634	NW_015441504	250034	A // G	0,01246089	0,04968995	n.s.	0,07992	0,02405	outlier	adhesion G-protein coupled receptor D1-like isoform X2
14564	AX-197965303	Apop_01799	NW_015441430	189825	A // G	2,00E-20	4,26E-18	outlier	0,10666	0,00013336	outlier	histamine H2 receptor-like
18130	AX-212317622	Apop_10104	NW_015441801	170807	A // G	5,07E-07	9,90E-06	outlier	0,056921	0,14489	n.s.	#N/D
3344	AX-212314397	Apop_06913	NW_015441088	1153437	C // G	5,56E-08	1,36E-06	outlier	0,037801	0,20387	n.s.	#N/D
13284	AX-212294957	AP_var_3610	NW_015441364	428986	A // G	1,50E-05	0,000207655	outlier	0,074267	0,055844	n.s.	#N/D
17756	AX-212314304	Apop_06820	NW_015441714	82865	A // T	2,23E-06	3,69E-05	outlier	0,037978	0,22732	n.s.	#N/D
10788	AX-198061726	Apop_01265	NW_015441263	438209	T // C	2,03E-06	3,36E-05	outlier	0,045396	0,17148	n.s.	#N/D
9447	AX-197964062	Apop_01067	NW_015441213	122378	A // C	0,009746701	0,04141175	n.s.	0,11263	0,00086359	outlier	rap guanine nucleotide exchange factor 1-like
15882	AX-212308991	AP_var_4361	NW_015441505	331751	A // G	1,67E-05	0,000229465	outlier	0,053273	0,11361	n.s.	#N/D
10094	AX-198078849	Apop_02751	NW_015441236	129054	A // T	2,29E-06	3,77E-05	outlier	0,03971	0,18226	n.s.	#N/D
18306	AX-212311673	Apop_04187	NW_015441838	49005	T // C	0,001373733	0,00932425	outlier	0,031694	0,29892	n.s.	#N/D
11086	AX-212303098	Apop_08868	NW_015441273	359766	T // C	4,78E-08	1,20E-06	outlier	0,028439	0,37036	n.s.	#N/D
13788	AX-197955671	Afix_11358	NW_015441378	157513	A // G	0,00149219	0,009798197	outlier	0,037958	0,26523	n.s.	#N/D
13790	AX-197955673	Afix_11360	NW_015441378	158804	A // G	0,001040636	0,007363636	outlier	0,038248	0,249	n.s.	#N/D
15798	AX-212303795	Apop_09565	NW_015441499	320129	A // G	0,000127542	0,001379246	outlier	0,022744	0,56928	n.s.	#N/D
10274	AX-212294026	AP_var_2679	NW_015441243	539471	A // T	0,000138181	0,0014859	outlier	0,030169	0,33427	n.s.	#N/D
5426	AX-212302181	Apop_07968	NW_015441118	253880	T // G	7,80E-05	0,000908529	outlier	0,024607	0,49894	n.s.	#N/D
7900	AX-212315940	Apop_08422	NW_015441171	143805	T // C	1,44E-05	0,000201684	outlier	0,024696	0,47944	n.s.	#N/D
12434	AX-198062093	Apop_01486	NW_015441326	202789	C // G	5,59E-05	0,00068387	outlier	0,026968	0,3778	n.s.	#N/D
10298	AX-212307336	AP_var_2703	NW_015441246	424203	T // C	0,001412257	0,009451669	outlier	0,033195	0,28856	n.s.	#N/D
8372	AX-212315988	Apop_08470	NW_015441183	327230	A // G	3,89E-05	0,000489999	outlier	0,028603	0,3965	n.s.	#N/D
11798	AX-198079040	Apop_02868	NW_015441302	486222	C // G	0,000161754	0,001633826	outlier	0,024833	0,47476	n.s.	#N/D
10190	AX-212316224	Apop_08706	NW_015441238	294911	A // C	7,27E-05	0,000852314	outlier	0,038055	0,21935	n.s.	#N/D
698	AX-212313209	Apop_05712	NW_015441058	660448	T // C	0,000179298	0,001787454	outlier	0,022182	0,60639	n.s.	#N/D
8972	AX-212306907	AP_var_2274	NW_015441198	497758	A // G	0,000208395	0,001982081	outlier	0,023089	0,58162	n.s.	#N/D
11754	AX-212307784	AP_var_3151	NW_015441300	397505	C // G	4,73E-05	0,00058768	outlier	0,022473	0,58371	n.s.	#N/D
16578	AX-212300879	Apop_06682	NW_015441569	103613	A // G	0,000187464	0,001854378	outlier	0,023272	0,5531	n.s.	#N/D
8958	AX-198061307	Apop_00993	NW_015441198	16327	T // C	5,77E-05	0,00069925	outlier	0,023211	0,53606	n.s.	#N/D
12452	AX-212294711	AP_var_3364	NW_015441326	300777	T // C	0,001435162	0,009546159	outlier	0,023463	0,53457	n.s.	#N/D
4942	AX-212305852	AP_var_1216	NW_015441112	787807	C // G	0,000999932	0,007195307	outlier	0,028334	0,3873	n.s.	#N/D
18574	AX-198058546	Afix_14783	NW_015441887	11273	A // T	0,001303175	0,00899809	outlier	0,026618	0,4176	n.s.	#N/D
19158	AX-212310311	AP_var_5681	NW_015442097	14158	T // C	0,001384948	0,009383736	outlier	0,021541	0,70006	n.s.	#N/D
4805	AX-198060551	Apop_00537	NW_015441108	892902	A // G	0,004734907	0,0240398	n.s.	0,095838	0,0022612	outlier	cartilage oligomeric matrix protein-like

14478	AX-212295321	AP_var_3974	NW_015441424	106457	C // G	5,15E-08	1,28E-06	outlier	0,10131	0,0010002	outlier	uncharacterized LOC107349606%2C transcript variant X2
152	AX-198078294	Apop_02393	NW_015441057	1002677	T // C	2,23E-31	1,42E-28	outlier	0,15054	0	outlier	#N/D
1052	AX-212304785	AP_var_0140	NW_015441060	1297056	T // G	6,63E-11	3,07E-09	outlier	0,087974	0,0035228	outlier	#N/D
5409	AX-197963223	Apop_00580	NW_015441117	808845	A // G	0,004741424	0,024041	n.s.	0,086707	0,0020764	outlier	F-box/LRR-repeat protein 2-like
9407	AX-198061406	Apop_01055	NW_015441211	476562	T // C	0,000705811	0,005393132	outlier	0,10796	0,0007104	outlier	#N/D
16944	AX-198063069	Apop_02104	NW_015441612	207259	T // G	3,97E-11	2,01E-09	outlier	0,07554	0,028742	outlier	#N/D
147	AX-197981178	Apop_02399	NW_015441057	975429	T // C	8,88E-05	0,001015233	outlier	0,13842	0	outlier	#N/D
8907	AX-197963923	Apop_00988	NW_015441196	583300	T // C	0,001926165	0,01218789	n.s.	0,11557	0,00021823	outlier	protein furry homolog-like
1416	AX-197981195	Apop_02408	NW_015441063	1004465	A // C	1,67E-06	2,84E-05	outlier	0,075891	0,033704	outlier	#N/D
5605	AX-198078663	Apop_02630	NW_015441124	619081	A // C	0,02157988	0,07164903	n.s.	0,077907	0,04252	outlier	basement membrane-specific heparan sulfate proteoglycan core protein-like
11264	AX-197981808	Apop_02789	NW_015441279	233625	A // T	8,25E-13	5,85E-11	outlier	0,1371	0	outlier	#N/D
15860	AX-212311192	Apop_03706	NW_015441504	253276	A // G	6,06E-10	2,23E-08	outlier	0,072271	0,04588	outlier	#N/D
10096	AX-212299476	Apop_05277	NW_015441236	129666	A // G	1,39E-07	3,05E-06	outlier	0,070168	0,041291	outlier	#N/D
10640	AX-212316306	Apop_08788	NW_015441259	11979	T // G	4,03E-09	1,29E-07	outlier	0,08999	0,0038095	outlier	#N/D
19650	AX-212310656	AP_var_6026	NW_015442939	2281	A // G	4,02E-07	8,02E-06	outlier	0,057817	0,082239	n.s.	#N/D
16706	AX-212297911	Apop_03711	NW_015441587	134642	C // G	1,08E-07	2,47E-06	outlier	0,053644	0,12157	n.s.	#N/D
11230	AX-197981861	Apop_02820	NW_015441279	43692	A // C	7,25E-09	2,12E-07	outlier	0,046262	0,14336	n.s.	#N/D
11746	AX-212316499	Apop_08981	NW_015441300	92786	A // G	2,83E-08	7,68E-07	outlier	0,040649	0,19478	n.s.	#N/D
4344	AX-212302073	Apop_07860	NW_015441104	702995	A // G	5,59E-06	8,63E-05	outlier	0,041364	0,19146	n.s.	#N/D
4340	AX-212302072	Apop_07859	NW_015441104	684750	A // C	6,28E-06	9,35E-05	outlier	0,040836	0,20633	n.s.	#N/D
17276	AX-212309597	AP_var_4967	NW_015441650	33162	T // C	4,02E-08	1,04E-06	outlier	0,030715	0,32124	n.s.	#N/D
15952	AX-212295725	AP_var_4381	NW_015441511	3564	T // C	9,42E-07	1,74E-05	outlier	0,037897	0,22574	n.s.	#N/D
10790	AX-212303033	Apop_08803	NW_015441263	447531	A // G	1,46E-05	0,000202851	outlier	0,035218	0,28366	n.s.	#N/D
7734	AX-212301194	Apop_06997	NW_015441169	185399	T // C	0,000886722	0,0065658	outlier	0,031319	0,3219	n.s.	#N/D
5422	AX-197963231	Apop_00584	NW_015441118	130092	A // C	8,38E-06	0,000121988	outlier	0,036303	0,2336	n.s.	#N/D
9628	AX-212298206	Apop_04006	NW_015441218	512200	C // G	2,91E-05	0,000376016	outlier	0,026202	0,4176	n.s.	#N/D
7732	AX-212314479	Apop_06995	NW_015441169	153101	A // G	0,000808009	0,006076999	outlier	0,02733	0,39863	n.s.	#N/D
10226	AX-197964275	Apop_01194	NW_015441240	466817	T // C	6,30E-06	9,35E-05	outlier	0,026412	0,42154	n.s.	#N/D
17444	AX-212311414	Apop_03928	NW_015441663	4328	A // G	0,000927362	0,006762087	outlier	0,027804	0,3965	n.s.	#N/D
954	AX-212312038	Apop_04552	NW_015441059	2074347	A // C	2,71E-08	7,41E-07	outlier	0,030949	0,3098	n.s.	#N/D
952	AX-212300237	Apop_06027	NW_015441059	2072842	T // G	2,91E-06	4,73E-05	outlier	0,026859	0,39968	n.s.	#N/D
16354	AX-212303896	Apop_09666	NW_015441544	289067	C // G	0,001405486	0,009449458	outlier	0,027239	0,39543	n.s.	#N/D
950	AX-212311325	Apop_03839	NW_015441059	2069866	A // G	1,20E-05	0,000171368	outlier	0,025478	0,44089	n.s.	#N/D
13298	AX-212303453	Apop_09223	NW_015441365	375272	A // C	0,00013342	0,001438746	outlier	0,024671	0,49106	n.s.	#N/D

13620	AX-212308301	AP_var_3668	NW_015441374	369986	A // C	9,48E-05	0,00107652	outlier	0,038746	0,21613	n.s.	#N/D
11464	AX-212294402	AP_var_3055	NW_015441287	186700	T // G	0,000300845	0,002684575	outlier	0,021886	0,64094	n.s.	#N/D
16044	AX-212317117	Apop_09599	NW_015441517	86395	T // C	3,47E-05	0,000438969	outlier	0,023809	0,54045	n.s.	#N/D
6700	AX-212300488	Apop_06278	NW_015441147	97513	C // G	0,000444211	0,00370482	outlier	0,021629	0,70006	n.s.	#N/D
10548	AX-197964352	Apop_01241	NW_015441254	370272	A // C	0,000637634	0,004921305	outlier	0,021869	0,64094	n.s.	#N/D
19084	AX-212317800	Apop_10283	NW_015442057	75620	A // C	0,000120859	0,001325694	outlier	0,021893	0,67702	n.s.	#N/D
16122	AX-212295784	AP_var_4440	NW_015441524	42830	A // G	0,001153996	0,008120752	outlier	0,024723	0,49575	n.s.	#N/D
12501	AX-197964840	Apop_01508	NW_015441330	427438	T // C	0,00269156	0,01582755	n.s.	0,10196	0,00043244	outlier	kinesin-like protein KIF16B
13224	AX-212303435	Apop_09205	NW_015441364	46908	T // C	1,10E-07	2,49E-06	outlier	0,068876	0,040476	outlier	#N/D
16946	AX-212304056	Apop_09826	NW_015441612	211737	A // C	3,97E-11	2,01E-09	outlier	0,079136	0,021471	outlier	#N/D
16043	AX-212295757	AP_var_4413	NW_015441517	79755	T // C	5,65E-06	8,66E-05	outlier	0,18878	0	outlier	#N/D
6814	AX-212293041	AP_var_1691	NW_015441151	333318	C // G	2,51E-10	1,07E-08	outlier	0,071377	0,02551	outlier	#N/D
19344	AX-212297133	AP_var_5789	NW_015442220	11900	A // G	1,92E-07	4,00E-06	outlier	0,14493	0,00013336	outlier	#N/D
19599	AX-212310621	AP_var_5991	NW_015442710	11711	T // G	0,000153155	0,001601923	outlier	0,11087	0	outlier	#N/D
6753	AX-198060921	Apop_00761	NW_015441148	809728	T // C	0,03887172	0,1073586	n.s.	0,074803	0,040072	outlier	LIM/homeobox protein Lhx1-like
17406	AX-197965879	Apop_02144	NW_015441658	15998	A // T	1,49E-06	2,59E-05	outlier	0,051172	0,13657	n.s.	#N/D
14344	AX-197965242	Apop_01762	NW_015441413	254569	T // C	2,01E-22	5,93E-20	outlier	0,10541	0,00043244	outlier	#N/D
15787	AX-212303786	Apop_09556	NW_015441499	72805	A // G	0,001999489	0,01246641	n.s.	0,090976	0,0012295	outlier	ankyrin repeat domain-containing protein 49-like
12139	AX-198062006	Apop_01432	NW_015441313	389636	T // C	0,05686621	0,1368328	n.s.	0,076705	0,031581	outlier	protein SDA1 homolog
7954	AX-212299947	Apop_05737	NW_015441175	389135	A // T	1,12E-07	2,52E-06	outlier	0,082272	0,0047596	outlier	#N/D
8903	AX-197981729	Apop_02740	NW_015441196	565251	T // C	0,001926165	0,01218789	n.s.	0,11656	0,00013336	outlier	POU domain%2C class 4%2C transcription factor 3-like%2C transcript variant X1
17986	AX-212304297	Apop_10067	NW_015441773	4960	T // C	5,34E-28	2,27E-25	outlier	0,13441	0	outlier	#N/D
17899	AX-212304263	Apop_10033	NW_015441740	125557	A // T	0,06560302	0,1504729	n.s.	0,07491	0,048087	outlier	exportin-6-like
4799	AX-212299849	Apop_05639	NW_015441108	866851	T // G	0,007414006	0,03354851	n.s.	0,08705	0,01404	outlier	uncharacterized LOC107333733
4797	AX-212312068	Apop_04582	NW_015441108	857916	A // T	0,007414006	0,03354851	n.s.	0,087904	0,011824	outlier	uncharacterized LOC107333733
18044	AX-212317597	Apop_10079	NW_015441785	98378	A // G	7,82E-19	1,50E-16	outlier	0,10899	0,00049869	outlier	#N/D
153	AX-212301503	Apop_07315	NW_015441057	1006913	T // C	5,68E-06	8,66E-05	outlier	0,14074	0	outlier	#N/D
4801	AX-212311825	Apop_04339	NW_015441108	870985	T // C	0,007414006	0,03354851	n.s.	0,085612	0,015505	outlier	uncharacterized LOC107333733
8051	AX-212302662	Apop_08432	NW_015441177	20093	T // G	0,006483528	0,03056743	n.s.	0,08123	0,016103	outlier	alanine aminotransferase 2-like
8057	AX-212306664	AP_var_2031	NW_015441177	445493	C // G	0,000570441	0,004521202	outlier	0,087013	0,0032475	outlier	ribosome-recycling factor%2C mitochondrial-like
15327	AX-212311184	Apop_03698	NW_015441470	202362	A // G	0,000642499	0,004948881	outlier	0,12226	3,16E-05	outlier	muscle%2C skeletal receptor tyrosine protein kinase-like%2C transcript variant X2
7272	AX-212315844	Apop_08326	NW_015441160	285745	A // G	1,60E-06	2,76E-05	outlier	0,081815	0,018987	outlier	astacin-like metalloprotease toxin 1
1424	AX-212300611	Apop_06401	NW_015441063	1216631	T // C	2,16E-11	1,16E-09	outlier	0,14753	0	outlier	kinesin-like protein KIF28P
11213	AX-197981794	Apop_02782	NW_015441279	18268	A // G	0,009104115	0,03902806	n.s.	0,070418	0,036644	outlier	RNA-binding protein 24-like%2C transcript variant X5

18836	AX-197961457	Afix_14975	NW_015441976	63005	A // G	3,46E-07	6,99E-06	outlier	0,036276	0,24748	n.s.	leucine-rich repeat-containing G-protein coupled receptor 4-like
2788	AX-198060176	Apop_00297	NW_015441078	817745	T // C	0,000764702	0,005808342	outlier	0,020674	0,88617	n.s.	CDK5 regulatory subunit-associated protein 3-like%2C transcript variant X1
19210	AX-212313206	Apop_05709	NW_015442130	11439	T // C	0,001278893	0,008853193	outlier	0,020663	0,88741	n.s.	HBS1-like protein
18703	AX-198063390	Apop_02312	NW_015441914	111855	A // G	0,03147884	0,09241264	n.s.	0,075991	0,037777	outlier	multiple epidermal growth factor-like domains protein 11 isoform X1
9966	AX-198061533	Apop_01136	NW_015441226	212787	A // G	1,58E-14	1,51E-12	outlier	0,19595	0	outlier	serine/threonine-protein kinase 33-like
19424	AX-212297179	AP_var_5835	NW_015442297	45060	T // C	0,000536674	0,004307077	outlier	0,031797	0,33106	n.s.	uncharacterized LOC107329179%2C transcript variant X3
2928	AX-197962769	Apop_00304	NW_015441079	1240224	A // G	0,001309227	0,00899809	outlier	0,021285	0,72879	n.s.	chorion transcription factor Cf2-like
2974	AX-212292017	AP_var_0658	NW_015441081	323651	C // G	1,91E-07	4,00E-06	outlier	0,027021	0,37724	n.s.	sorcin-like
696	AX-197962331	Apop_00044	NW_015441058	654707	A // G	0,000179298	0,001787454	outlier	0,022782	0,60043	n.s.	uncharacterized LOC107331131%2C transcript variant X1
3230	AX-212315150	Apop_07649	NW_015441084	438047	A // G	0,000575669	0,004553212	outlier	0,023202	0,55392	n.s.	chromodomain-helicase-DNA-binding protein 2-like
3232	AX-212315152	Apop_07651	NW_015441084	481056	T // G	0,001008981	0,007242988	outlier	0,020632	0,86676	n.s.	cyclic AMP-responsive element-binding protein 3-like protein 3-B%2C transcript variant X1
3752	AX-212315247	Apop_07746	NW_015441093	525597	T // C	6,30E-05	0,000753754	outlier	0,02377	0,51781	n.s.	serine-protein kinase ATM-like
3880	AX-198060398	Apop_00437	NW_015441095	398522	A // G	1,50E-05	0,000207655	outlier	0,022451	0,57544	n.s.	E3 ubiquitin-protein ligase HECTD3-like
3936	AX-197963003	Apop_00445	NW_015441096	5818	A // T	5,42E-06	8,41E-05	outlier	0,023214	0,57251	n.s.	sodium/potassium/calcium exchanger 3-like%2C transcript variant X2
4106	AX-212305619	AP_var_0983	NW_015441098	561168	A // G	4,49E-07	8,86E-06	outlier	0,064781	0,09319	n.s.	FRAS1-related extracellular matrix protein 2-like
4258	AX-212315350	Apop_07849	NW_015441103	973914	T // C	0,000970595	0,007010564	outlier	0,023189	0,54561	n.s.	uncharacterized LOC107333259%2C transcript variant X2
4806	AX-212313533	Apop_06036	NW_015441108	897494	T // G	7,12E-05	0,000841647	outlier	0,023182	0,55392	n.s.	cartilage oligomeric matrix protein-like
16799	AX-198063021	Apop_02073	NW_015441589	165791	A // T	0,01503708	0,05657141	n.s.	0,088228	0,018328	outlier	MFS-type transporter SLC18B1-like
4808	AX-212297958	Apop_03758	NW_015441108	903330	T // G	7,12E-05	0,000841647	outlier	0,023044	0,54841	n.s.	cartilage oligomeric matrix protein-like
4812	AX-212315406	Apop_07905	NW_015441108	1003680	A // G	0,001309185	0,00899809	outlier	0,02171	0,62249	n.s.	uncharacterized LOC107333792%2C transcript variant X5
4898	AX-198060574	Apop_00552	NW_015441112	305931	T // C	5,95E-06	9,00E-05	outlier	0,030385	0,33936	n.s.	bone morphogenetic protein 2-A-like%2C transcript variant X2
978	AX-212291493	AP_var_0134	NW_015441060	810542	T // C	6,04E-06	9,10E-05	outlier	0,029848	0,32651	n.s.	uncharacterized LOC107335171
5672	AX-212292741	AP_var_1391	NW_015441127	52538	A // G	6,34E-09	1,88E-07	outlier	0,049327	0,1253	n.s.	multidrug resistance-associated protein 1-like
6134	AX-198060815	Apop_00691	NW_015441136	896861	T // G	1,48E-06	2,58E-05	outlier	0,067337	0,058469	n.s.	potassium voltage-gated channel subfamily H member 6-like%2C transcript variant X1
1162	AX-212313369	Apop_05872	NW_015441061	1126240	T // C	2,38E-09	7,84E-08	outlier	0,061271	0,064048	n.s.	bifunctional glutamate/proline--tRNA ligase-like
8905	AX-198078837	Apop_02743	NW_015441196	578377	T // C	0,007717947	0,03460587	n.s.	0,095333	0,0028709	outlier	protein furry homolog-like
6756	AX-212302429	Apop_08199	NW_015441148	818007	C // G	1,09E-06	1,95E-05	outlier	0,028595	0,36451	n.s.	probable RNA-binding protein 19%2C transcript variant X1

7002	AX-212298432	Apop_04232	NW_01544115	196820	A // C	5,43E-07	1,04E-05	outlier	0,029728	0,32519	n.s.	uncharacterized LOC107337339%2C transcript variant X1
18702	AX-212304419	Apop_10189	NW_01544191	104335	T // C	7,35E-08	1,74E-06	outlier	0,075789	0,042925	outlier	uncharacterized LOC107358661
4798	AX-212298783	Apop_04583	NW_01544110	865769	A // C	5,00E-11	2,42E-09	outlier	0,083921	0,017676	outlier	uncharacterized LOC107333733
4802	AX-212313608	Apop_06111	NW_01544110	872768	A // G	1,47E-12	1,03E-10	outlier	0,10186	0,0012295	outlier	uncharacterized LOC107333733
7474	AX-212306504	AP_var_1868	NW_01544116	456350	T // G	0,000270613	0,002454859	outlier	0,021805	0,63472	n.s.	transcription initiation factor IIB-like
17436	AX-212313348	Apop_05851	NW_01544166	98206	T // C	7,10E-09	2,09E-07	outlier	0,096154	0,034426	outlier	E3 ubiquitin-protein ligase MARCH6-like
7528	AX-197963675	Apop_00844	NW_01544116	547812	A // T	3,47E-07	6,99E-06	outlier	0,054189	0,10786	n.s.	HEAT repeat-containing protein 6-like
1286	AX-197962477	Apop_00135	NW_01544106	738286	A // G	0,000109309	0,001216432	outlier	0,023494	0,53871	n.s.	A disintegrin and metalloproteinase with thrombospondin motifs 18-like
1392	AX-212314931	Apop_07430	NW_01544106	1306162	A // G	0,001209158	0,008416103	outlier	0,024602	0,49324	n.s.	DNA polymerase epsilon catalytic subunit A-like
7692	AX-212315913	Apop_08395	NW_01544116	665928	T // C	0,000305801	0,002722456	outlier	0,030352	0,33555	n.s.	inactive tyrosine-protein kinase transmembrane receptor ROR1-like%2C transcript variant X1
7764	AX-212315926	Apop_08408	NW_01544116	590252	A // G	1,82E-06	3,07E-05	outlier	0,029494	0,34063	n.s.	WD repeat-containing protein 1-A-like
7964	AX-212315949	Apop_08431	NW_01544117	284230	T // C	0,001434878	0,009546159	outlier	0,020335	0,88567	n.s.	myoferlin-like
8594	AX-212306818	AP_var_2185	NW_01544119	239315	A // G	0,000877659	0,00652393	outlier	0,02094	0,84061	n.s.	TBC1 domain family member 22B-like
13221	AX-212316720	Apop_09202	NW_01544136	3975	T // G	0,03384689	0,09734899	n.s.	0,06828	0,046726	outlier	probable ubiquitin carboxyl-terminal hydrolase FAF-X
1428	AX-212314334	Apop_06850	NW_01544106	1224375	A // G	0,000203379	0,00194642	outlier	0,076578	0,074687	n.s.	kinesin-like protein KIF28P
1482	AX-212312333	Apop_04848	NW_01544106	1618684	A // T	0,000144989	0,001541786	outlier	0,025101	0,46756	n.s.	granulins-like
9290	AX-198061377	Apop_01036	NW_01544120	691116	A // G	5,13E-09	1,56E-07	outlier	0,041222	0,20469	n.s.	zinc transporter 2-like
13223	AX-212316722	Apop_09204	NW_01544136	33014	C // G	0,03384689	0,09734899	n.s.	0,068114	0,04946	outlier	probable ubiquitin carboxyl-terminal hydrolase FAF-X
10062	AX-212302909	Apop_08679	NW_01544123	338060	A // G	0,001025296	0,007314654	outlier	0,024006	0,53308	n.s.	fibrocystin-L-like
10110	AX-198061596	Apop_01176	NW_01544123	450295	T // C	8,46E-06	0,000122642	outlier	0,025391	0,43445	n.s.	cathepsin O-like
716	AX-212304699	AP_var_0054	NW_01544105	1122310	T // C	0,000956042	0,006931604	outlier	0,092029	0,037398	outlier	polypeptide N-acetylgalactosaminyltransferase 2-like
10118	AX-212302929	Apop_08699	NW_01544123	181733	T // C	5,72E-06	8,68E-05	outlier	0,02435	0,49964	n.s.	uncharacterized LOC107342233
10506	AX-197964332	Apop_01228	NW_01544125	122477	C // G	5,05E-05	0,000624202	outlier	0,059262	0,11719	n.s.	frizzled-4-like
10608	AX-212307437	AP_var_2804	NW_01544125	459334	C // G	0,000431033	0,003618563	outlier	0,022146	0,60105	n.s.	EF-hand calcium-binding domain-containing protein 6-like
11082	AX-198061801	Apop_01314	NW_01544127	233235	C // G	0,000455113	0,003762953	outlier	0,021303	0,86551	n.s.	epithelial splicing regulatory protein 1-like
11210	AX-198078883	Apop_02778	NW_01544127	17008	A // G	1,09E-16	1,61E-14	outlier	0,08022	0,0072874	outlier	RNA-binding protein 24-like%2C transcript variant X5
11212	AX-197981793	Apop_02781	NW_01544127	17768	A // G	8,37E-10	2,99E-08	outlier	0,059687	0,079935	n.s.	RNA-binding protein 24-like%2C transcript variant X5
17618	AX-212296432	AP_var_5088	NW_01544169	19095	A // G	0,000519103	0,004192429	outlier	0,084422	0,01238	outlier	transient receptor potential cation channel subfamily A member 1-like
11356	AX-212316428	Apop_08910	NW_01544127	461364	A // G	3,83E-09	1,23E-07	outlier	0,066779	0,078201	n.s.	uncharacterized LOC107344204

11394	AX-198061859	Apop_01351	NW_01544128C	372492	T // C	0,000151197	0,001590131	outlier	0,040131	0,22335	n.s.	uncharacterized LOC107344225
12905	AX-212316662	Apop_09144	NW_01544135З	382885	A // C	3,00E-39	5,74E-36	outlier	0,31008	0	outlier	tumor necrosis factor receptor superfamily member 16-like, transcript variant X2
1652	AX-212314962	Apop_07461	NW_015441067	180627	T // C	0,000246057	0,002275233	outlier	0,020643	0,88932	n.s.	vinculin-like
10074	AX-212316203	Apop_08685	NW_015441234	20888	C // G	2,59E-05	0,000341459	outlier	0,026225	0,40699	n.s.	phospholipase B-like 1
18572	AX-198058578	Afix_14802	NW_015441887	3699	T // C	0,000805232	0,006068033	outlier	0,027289	0,40179	n.s.	UDP-glucuronic acid decarboxylase 1-like
14814	AX-212316979	Apop_09461	NW_01544143S	372460	A // G	7,49E-10	2,73E-08	outlier	0,070914	0,073502	n.s.	uncharacterized LOC107349989
17974	AX-212296515	AP_var_5171	NW_015441767	133946	A // T	6,67E-06	9,86E-05	outlier	0,021479	0,67702	n.s.	uncharacterized LOC107356830
19426	AX-197958124	Afix_12928	NW_015442298	49123	T // C	0,000549229	0,004362113	outlier	0,020869	0,88193	n.s.	microtubule-actin cross-linking factor 1-like
7674	AX-212315910	Apop_08392	NW_01544116E	463088	T // C	3,20E-05	0,000409338	outlier	0,023987	0,51846	n.s.	tubulin polyglutamylase TTLL7-like
11782	AX-198079006	Apop_02849	NW_015441302	467158	C // G	0,000161754	0,001633826	outlier	0,024433	0,49179	n.s.	sushi%2C von Willebrand factor type A%2C EGF and pentraxin domain-containing protein 1-like%2C transcript variant X1
12416	AX-198062082	Apop_01479	NW_015441324	214139	A // G	4,50E-06	7,14E-05	outlier	0,036311	0,23593	n.s.	patatin-like phospholipase domain-containing protein 7
17934	AX-197966025	Apop_02228	NW_015441751	125379	T // C	1,47E-08	4,21E-07	outlier	0,059413	0,077038	n.s.	phosphate carrier protein%2C mitochondrial-like
1686	AX-212314966	Apop_07465	NW_015441067	818850	C // G	1,33E-09	4,61E-08	outlier	0,041046	0,21855	n.s.	uncharacterized LOC107345109
12825	AX-197938643	Afix_00587	NW_015441352	30216	A // G	0,004430687	0,02298293	n.s.	0,087482	0,0036621	outlier	prominin-1-A-like isoform X1
16571	AX-212313652	Apop_06155	NW_01544156S	47572	A // G	2,25E-08	6,25E-07	outlier	0,14785	0	outlier	leucine--tRNA ligase, cytoplasmic-like
12500	AX-212312378	Apop_04893	NW_01544133C	422240	A // C	0,000784106	0,005932191	outlier	0,028448	0,36034	n.s.	kinesin-like protein KIF16B
12488	AX-197964831	Apop_01503	NW_01544133C	150971	C // G	0,000611381	0,004776467	outlier	0,022005	0,61955	n.s.	early endosome antigen 1-like%2C transcript variant X5
16975	AX-212312304	Apop_04819	NW_015441618	251815	A // G	0,02865449	0,08664621	n.s.	0,080415	0,017361	outlier	fibroblast growth factor receptor 3-like
12426	AX-212305005	AP_var_0360	NW_015441325	365185	T // C	0,000155798	0,001625119	outlier	0,021607	0,82425	n.s.	uncharacterized LOC107347339%2C transcript variant X2
11260	AX-212316423	Apop_08905	NW_015441279	219768	A // G	2,53E-12	1,70E-10	outlier	0,13424	0	outlier	protein FAM65B-like
13784	AX-197955665	Afix_11353	NW_015441378	138745	A // C	0,001040636	0,007363636	outlier	0,037321	0,25722	n.s.	uncharacterized LOC107348149
19205	AX-212314099	Apop_06615	NW_015442124	32540	A // C	0,01357767	0,05255567	n.s.	0,073977	0,04376	outlier	adhesion G-protein coupled receptor D1-like%2C transcript variant X1
1854	AX-212305032	AP_var_0387	NW_01544106S	460889	C // G	0,0003744	0,003199254	outlier	0,023678	0,55119	n.s.	fibulin-1-like
14306	AX-212316911	Apop_09393	NW_01544140S	334414	T // G	0,00012365	0,001344751	outlier	0,058721	0,14034	n.s.	dehydrogenase/reductase SDR family member 7-like%2C transcript variant X1
14530	AX-212303661	Apop_09431	NW_015441428	345729	T // C	3,15E-09	1,02E-07	outlier	0,046888	0,14795	n.s.	exocyst complex component 3-like
15212	AX-198062675	Apop_01856	NW_015441458	302868	T // C	5,63E-05	0,000686996	outlier	0,022242	0,6252	n.s.	putative beta-lactamase-like 1
15320	AX-198062713	Apop_01878	NW_01544146S	37944	T // C	0,000146382	0,001547996	outlier	0,02133	0,8722	n.s.	uncharacterized LOC107350829%2C transcript variant X1
16395	AX-198062917	Apop_02006	NW_01544154S	234133	T // C	0,000627617	0,004875491	outlier	0,103	0,00045226	outlier	transcription factor 4-like
15318	AX-198062712	Apop_01877	NW_01544146S	32348	A // G	0,000146382	0,001547996	outlier	0,021144	0,87389	n.s.	uncharacterized LOC107350829%2C transcript variant X1

18720	AX-212304426	Apop_10196	NW_015441923	61746	A // G	5,78E-08	1,40E-06	outlier	0,083043	0,03969	outlier	EGF and laminin G domain-containing protein-like
15796	AX-212303794	Apop_09564	NW_015441499	216349	A // T	3,50E-08	9,24E-07	outlier	0,052869	0,12831	n.s.	transcription factor E2F4-like
18028	AX-212303782	Apop_09552	NW_015441783	38448	T // C	0,000202916	0,00194642	outlier	0,021215	0,88617	n.s.	uncharacterized protein PB18E9.04c-like
15806	AX-197965519	Apop_01929	NW_015441501	48268	A // G	0,000445815	0,003710115	outlier	0,069516	0,094957	n.s.	probable protein phosphatase 2C T23F11.1
15864	AX-212299900	Apop_05690	NW_015441504	282089	T // G	0,000930315	0,006770724	outlier	0,023867	0,54103	n.s.	adhesion G-protein coupled receptor G2-like
14629	AX-197956569	Afix_11969	NW_015441435	232323	A // T	0,002907476	0,01681314	n.s.	0,098173	0,0010514	outlier	prominin-2-like
3574	AX-212315229	Apop_07728	NW_015441091	502331	T // C	2,37E-17	4,33E-15	outlier	0,091335	0,0026496	outlier	polycystic kidney disease 1-related protein
16256	AX-197965600	Apop_01982	NW_015441536	96580	T // C	0,000602383	0,004715791	outlier	0,021847	0,84061	n.s.	transformation/transcription domain-associated protein-like
2182	AX-212301719	Apop_07506	NW_015441072	323585	T // C	1,30E-05	0,0001846	outlier	0,036703	0,24058	n.s.	uncharacterized LOC107352748
19206	AX-212297485	Apop_03285	NW_015442124	34184	T // C	4,03E-10	1,57E-08	outlier	0,07829	0,0266	outlier	adhesion G-protein coupled receptor D1-like%2C transcript variant X1
16560	AX-212303950	Apop_09720	NW_015441567	114729	C // G	0,000926011	0,006762087	outlier	0,0215	0,83257	n.s.	GTPase-activating Rap/Ran-GAP domain-like protein 3
16608	AX-212295993	AP_var_4649	NW_015441572	220214	A // C	1,14E-07	2,56E-06	outlier	0,027929	0,35491	n.s.	tolloid-like protein 2
16668	AX-212317262	Apop_09744	NW_015441579	168933	A // C	0,000297699	0,002662715	outlier	0,020668	0,87309	n.s.	uncharacterized LOC107353541%2C transcript variant X2
16694	AX-212296046	AP_var_4702	NW_015441584	254288	C // G	0,000279504	0,002523555	outlier	0,043668	0,16736	n.s.	nucleoporin NDC1-like%2C transcript variant X1
14320	AX-212316915	Apop_09397	NW_015441411	61967	A // G	5,47E-10	2,07E-08	outlier	0,10455	0,00021823	outlier	sperm receptor for egg jelly-like
16864	AX-197965766	Apop_02083	NW_015441599	127776	A // G	0,000313519	0,002771831	outlier	0,021148	0,87555	n.s.	uncharacterized LOC107353948
17046	AX-212317367	Apop_09849	NW_015441625	148197	A // G	1,87E-05	0,000256174	outlier	0,042824	0,17312	n.s.	aquaporin-5-like
17118	AX-212317379	Apop_09861	NW_015441631	128770	A // T	0,0002851	0,002568026	outlier	0,024059	0,53247	n.s.	uncharacterized LOC107354539
15853	AX-212312393	Apop_04908	NW_015441504	203048	A // G	0,01246089	0,04968995	n.s.	0,078753	0,022949	outlier	adhesion G-protein coupled receptor D1-like%2C transcript variant X2
7527	AX-198061051	Apop_00843	NW_015441164	546048	A // G	0,01536972	0,05724318	n.s.	0,072141	0,037016	outlier	HEAT repeat-containing protein 6-like
17798	AX-198063216	Apop_02199	NW_015441725	133557	A // T	3,47E-05	0,000438969	outlier	0,022067	0,60456	n.s.	putative ammonium transporter 3%2C transcript variant X1
4774	AX-212315389	Apop_07888	NW_015441108	416183	A // C	7,81E-10	2,82E-08	outlier	0,074481	0,041702	outlier	gamma-aminobutyric acid type B receptor subunit 2-like%2C transcript variant X3
17900	AX-198063242	Apop_02216	NW_015441742	48356	A // T	8,14E-05	0,00094128	outlier	0,021065	0,82756	n.s.	sulfotransferase 1C4-like
18124	AX-212304330	Apop_10100	NW_015441801	65947	A // G	6,00E-10	2,23E-08	outlier	0,068485	0,065818	n.s.	uncharacterized LOC107357306
18178	AX-212298029	Apop_03829	NW_015441812	124867	A // G	0,000183545	0,001825042	outlier	0,022163	0,60209	n.s.	uncharacterized LOC107357440
7140	AX-212302512	Apop_08282	NW_015441157	435968	C // G	1,55E-15	1,80E-13	outlier	0,1043	0,00021823	outlier	uncharacterized LOC107337495%2C transcript variant X2
18838	AX-212317740	Apop_10223	NW_015441976	90319	A // T	0,000510176	0,004137789	outlier	0,021091	0,88537	n.s.	uncharacterized LOC107327218
1394	AX-212314932	Apop_07431	NW_015441062	1346253	T // C	0,000110257	0,001223428	outlier	0,020804	0,85977	n.s.	uncharacterized LOC107339563
10812	AX-212312775	Apop_05289	NW_015441265	13904	T // C	4,74E-06	7,44E-05	outlier	0,024196	0,5045	n.s.	multidrug resistance-associated protein 1-like
12450	AX-212307996	AP_var_3363	NW_015441326	291774	A // G	3,76E-08	9,80E-07	outlier	0,046215	0,16158	n.s.	uncharacterized LOC107346133%2C transcript variant X4

1862	AX-212305038	AP_var_0393	NW_015441069	632762	A // T	0,000911802	0,006725486	outlier	0,022126	0,63917	n.s.	allene oxide synthase-lipoxygenase protein-like
14246	AX-198062492	Apop_01747	NW_015441408	153571	T // C	0,000991442	0,007147655	outlier	0,020972	0,84296	n.s.	uncharacterized LOC107349101
1904	AX-198038158	Afix_02308	NW_015441069	1292211	A // G	0,000333638	0,002886725	outlier	0,021232	0,76894	n.s.	octopamine receptor 1-like%2C transcript variant X3
1902	AX-198038156	Afix_02307	NW_015441069	1291372	A // G	0,000333638	0,002886725	outlier	0,021376	0,84945	n.s.	octopamine receptor 1-like%2C transcript variant X3
12012	AX-198061980	Apop_01418	NW_015441307	472343	A // G	0,001326746	0,00908587	outlier	0,025437	0,47594	n.s.	#N/D
5640	AX-197963280	Apop_00614	NW_015441126	281548	A // C	4,60E-12	2,89E-10	outlier	0,096795	0,0013651	outlier	transient receptor potential cation channel subfamily A member 1-like%2C transcript variant X3
9425	AX-212310718	Apop_03232	NW_015441211	574303	A // T	0,006712988	0,03130137	n.s.	0,097134	0,0031115	outlier	uncharacterized protein LOC107340839
15854	AX-212298916	Apop_04716	NW_015441504	207032	A // G	4,03E-10	1,57E-08	outlier	0,078824	0,033343	outlier	adhesion G-protein coupled receptor D1-like%2C transcript variant X9
15858	AX-212299899	Apop_05689	NW_015441504	249868	T // C	4,03E-10	1,57E-08	outlier	0,078263	0,030877	outlier	adhesion G-protein coupled receptor D1-like%2C transcript variant X9
15856	AX-212300637	Apop_06439	NW_015441504	234182	C // G	4,60E-09	1,44E-07	outlier	0,074555	0,042112	outlier	adhesion G-protein coupled receptor D1-like%2C transcript variant X9
18770	AX-197966200	Apop_02323	NW_015441949	114812	A // C	2,69E-06	4,38E-05	outlier	0,023847	0,50655	n.s.	pantothenate kinase 4-like
4088	AX-198060418	Apop_00451	NW_015441097	1056888	T // C	0,000106631	0,001190089	outlier	0,03428	0,27518	n.s.	uncharacterized protein LOC107332647
4306	AX-212315354	Apop_07853	NW_015441104	351285	T // G	0,000152947	0,001601923	outlier	0,027729	0,3949	n.s.	ephrin-B2a-like isoform X1
4312	AX-198060493	Apop_00500	NW_015441104	559898	A // G	1,66E-06	2,83E-05	outlier	0,02533	0,44225	n.s.	lipopolysaccharide-induced tumor necrosis factor-alpha factor homolog
11454	AX-198061868	Apop_01358	NW_015441285	422104	A // C	1,02E-06	1,85E-05	outlier	0,10022	0,044183	outlier	thyroid receptor-interacting protein 11-like%2C transcript variant X2
16396	AX-212317201	Apop_09683	NW_015441549	244391	T // C	9,96E-22	2,72E-19	outlier	0,1095	0,00025867	outlier	transcription factor 12-like
7144	AX-212293136	AP_var_1786	NW_015441157	464680	A // G	1,26E-20	2,84E-18	outlier	0,13412	0	outlier	uncharacterized LOC107337462
1390	AX-198078313	Apop_02405	NW_015441062	1295211	A // T	0,001209158	0,008416103	outlier	0,024243	0,52038	n.s.	DNA polymerase epsilon catalytic subunit A-like
1384	AX-198059881	Apop_00118	NW_015441062	1273232	A // C	0,00120785	0,008416103	outlier	0,023268	0,53096	n.s.	golgin subfamily A member 4-like
10098	AX-197964230	Apop_01166	NW_015441236	153074	A // G	0,00130828	0,00899809	outlier	0,022481	0,59726	n.s.	CUB and sushi domain-containing protein 1-like
10514	AX-198061681	Apop_01236	NW_015441253	163719	A // T	0,000171291	0,001716571	outlier	0,050248	0,16239	n.s.	uncharacterized protein LOC107343009
10822	AX-212294232	AP_var_2885	NW_015441265	256850	A // G	1,89E-05	0,000257739	outlier	0,065917	0,095551	n.s.	rap1 GTPase-activating protein 2-like
16397	AX-197965641	Apop_02007	NW_015441549	245728	C // G	0,008436644	0,03703769	n.s.	0,07534	0,023318	outlier	transcription factor 12-like
11784	AX-197981915	Apop_02852	NW_015441302	473374	A // T	0,000212306	0,002011735	outlier	0,023224	0,54841	n.s.	E3 ubiquitin-protein ligase TRIM71-like
8898	AX-197981714	Apop_02732	NW_015441196	554973	T // C	3,69E-12	2,35E-10	outlier	0,086479	0,0092556	outlier	uncharacterized LOC107339948
12954	AX-198062264	Apop_01596	NW_015441355	255868	T // G	0,0005947	0,004665187	outlier	0,025528	0,47634	n.s.	uncharacterized protein LOC107347239
16574	AX-198062487	Apop_01744	NW_015441569	96827	A // G	5,29E-12	3,27E-10	outlier	0,068707	0,055339	n.s.	uncharacterized LOC107348982
10039	AX-212293908	AP_var_2561	NW_015441230	523223	A // G	0,004724068	0,02401662	n.s.	0,11063	6,67E-05	outlier	#N/D
16840	AX-198051718	Afix_10666	NW_015441598	109569	T // G	1,54E-07	3,30E-06	outlier	0,04954	0,14109	n.s.	kelch-like protein diablo
18168	AX-197966095	Apop_02267	NW_015441809	158744	T // C	7,80E-08	1,83E-06	outlier	0,027576	0,37724	n.s.	probable ATP-dependent helicase PF08_0048

18436	AX-197966144	Apop_02293	NW_015441856	14368	A // G	2,19E-05	0,000296094	outlier	0,031675	0,30642	n.s.	LOW QUALITY PROTEIN: uncharacterized protein LOC107358039
18704	AX-197966182	Apop_02313	NW_015441914	142444	A // T	0,000334055	0,002886725	outlier	0,023552	0,54219	n.s.	uncharacterized protein LOC107358670
10815	AX-212301258	Apop_07061	NW_015441265	85587	T // G	0,002994062	0,01723574	n.s.	0,11596	6,67E-05	outlier	#N/D
4782	AX-197963147	Apop_00533	NW_015441108	467950	A // T	0,001467067	0,009699784	outlier	0,020482	0,88416	n.s.	cartilage oligomeric matrix protein-like
16508	AX-198062948	Apop_02027	NW_015441561	105670	A // C	2,64E-07	5,38E-06	outlier	0,034003	0,27589	n.s.	#N/D
19077	AX-197966246	Apop_02347	NW_015442053	88733	T // G	0,02204423	0,07300083	n.s.	0,08761	0,0061609	outlier	#N/D
16001	AX-198062845	Apop_01958	NW_015441516	9052	T // C	0,0170652	0,06082721	n.s.	0,078931	0,012933	outlier	#N/D
5606	AX-198078664	Apop_02631	NW_015441124	619782	T // G	5,38E-08	1,32E-06	outlier	0,05383	0,11073	n.s.	basement membrane-specific heparan sulfate proteoglycan core protein-like
9458	AX-197964070	Apop_01072	NW_015441214	33995	T // C	0,000755614	0,005762181	outlier	0,020434	0,88759	n.s.	ADP-ribosyl cyclase/cyclic ADP-ribose hydrolase-like
13739	AX-212303513	Apop_09283	NW_015441377	153490	A // G	0,004902527	0,02463116	n.s.	0,089763	0,013485	outlier	#N/D
13736	AX-212308310	AP_var_3677	NW_015441377	52798	T // G	0,00018532	0,001837915	outlier	0,02959	0,34189	n.s.	uncharacterized LOC107350302
14021	AX-212299968	Apop_05758	NW_015441389	398790	T // C	0,01246089	0,04968995	n.s.	0,078267	0,028385	outlier	#N/D
9212	AX-212316073	Apop_08555	NW_015441206	576036	C // G	0,00088006	0,0065291	outlier	0,070742	0,043343	outlier	uncharacterized LOC107340492
7422	AX-198039684	Afix_03262	NW_015441162	610666	A // G	4,98E-09	1,54E-07	outlier	0,081801	0,01321	outlier	uncharacterized protein LOC107337856
19110	AX-198063451	Apop_02352	NW_015442074	60506	T // C	2,06E-31	1,42E-28	outlier	0,10791	3,16E-05	outlier	uncharacterized protein LOC107328041
14354	AX-198062530	Apop_01768	NW_015441414	124105	A // C	2,28E-14	2,03E-12	outlier	0,081095	0,010994	outlier	golgin subfamily B member 1-like
7420	AX-212306497	AP_var_1861	NW_015441162	599413	C // G	3,41E-08	9,07E-07	outlier	0,070872	0,047175	outlier	putative aminopeptidase W07G4.4
150	AX-197962311	Apop_00032	NW_015441057	990159	A // G	6,97E-30	3,34E-27	outlier	0,14242	0	outlier	uncharacterized protein LOC107357903
4804	AX-198060550	Apop_00536	NW_015441108	884786	C // G	2,08E-11	1,14E-09	outlier	0,087147	0,011546	outlier	uncharacterized protein LOC107333733
12398	AX-212307974	AP_var_3341	NW_015441323	276867	T // C	1,14E-09	4,04E-08	outlier	0,10363	0,0084315	outlier	major facilitator superfamily domain-containing protein 12-like
2846	AX-197962775	Apop_00308	NW_015441079	436535	T // C	1,81E-16	2,47E-14	outlier	0,14777	0	outlier	uncharacterized protein LOC107329195 isoform X2
11229	AX-197964070	Apop_01072	NW_015441279	33995	T // C	0,00118145	0,008283491	outlier	0,077531	0,010213	outlier	ADP-ribosyl cyclase/cyclic ADP-ribose hydrolase-like
7310	AX-198061009	Apop_00816	NW_015441161	339133	A // G	1,04E-13	8,69E-12	outlier	0,11407	0,0005812	outlier	protein KTI12 homolog
14340	AX-197965240	Apop_01761	NW_015441413	206838	T // C	2,41E-15	2,64E-13	outlier	0,09845	0,001579	outlier	uncharacterized protein LOC107349266 isoform X1
1493	AX-212291613	AP_var_0254	NW_015441064	1323058	A // G	0,02026556	0,06823213	n.s.	0,1036	0,0005812	outlier	#N/D
17465	AX-212304156	Apop_09926	NW_015441665	219233	A // C	0,002528228	0,01507551	n.s.	0,1198	3,16E-05	outlier	#N/D
10041	AX-212307195	AP_var_2562	NW_015441230	530855	C // G	0,01563572	0,0578759	n.s.	0,082306	0,0090504	outlier	#N/D
5404	AX-197963214	Apop_00575	NW_015441117	722866	A // G	4,11E-11	2,01E-09	outlier	0,072168	0,027317	outlier	protein phosphatase 1D-like
12901	AX-198062238	Apop_01580	NW_015441353	350801	A // G	4,39E-10	1,70E-08	outlier	0,22371	0	outlier	mitochondrial amidoxime reducing component 2-like isoform X2
17596	AX-198063182	Apop_02177	NW_015441695	155086	T // C	4,48E-09	1,42E-07	outlier	0,078884	0,017044	outlier	probable ubiquitin carboxyl-terminal hydrolase FAF-X
10100	AX-198061583	Apop_01168	NW_015441236	174152	A // G	4,96E-08	1,24E-06	outlier	0,076381	0,021845	outlier	LOW QUALITY PROTEIN: E3 ubiquitin-protein ligase HERC2-like
5994	AX-198060785	Apop_00673	NW_015441133	814755	A // G	1,32E-25	4,59E-23	outlier	0,10205	0,00064487	outlier	protocadherin-like protein

15866	AX-212313511	Apop_06014	NW_015441504	287819	A // G	4,03E-10	1,57E-08	outlier	0,077378	0,02945	outlier	adhesion G-protein coupled receptor G2-like
15794	AX-212303792	Apop_09562	NW_015441499	172789	T // C	3,52E-17	6,13E-15	outlier	0,095947	0,00064487	outlier	LOW QUALITY PROTEIN: AP-1 complex subunit gamma-1-like
11272	AX-197964542	Apop_01345	NW_015441279	244375	A // G	8,01E-13	5,78E-11	outlier	0,13503	0	outlier	solute carrier family 22 member 15-like isoform X2
11278	AX-198078913	Apop_02797	NW_015441279	260525	T // C	8,01E-13	5,78E-11	outlier	0,13713	0	outlier	solute carrier family 22 member 15-like isoform X2
5408	AX-198060619	Apop_00579	NW_015441117	808655	A // G	4,11E-11	2,01E-09	outlier	0,073594	0,024416	outlier	F-box/LRR-repeat protein 2-like
5410	AX-197963225	Apop_00581	NW_015441117	809276	T // C	1,13E-10	5,09E-09	outlier	0,069537	0,03969	outlier	F-box/LRR-repeat protein 2-like
2906	AX-212311091	Apop_03605	NW_015441079	1112357	T // G	0,000293919	0,002635063	outlier	0,021626	0,67148	n.s.	peroxisome biogenesis factor 1-like
3270	AX-197962856	Apop_00360	NW_015441085	402145	C // G	0,000167988	0,001692331	outlier	0,021562	0,72879	n.s.	PR domain zinc finger protein 14-like
3308	AX-197962868	Apop_00367	NW_015441087	274693	T // C	4,79E-06	7,49E-05	outlier	0,024461	0,50347	n.s.	tartrate-resistant acid phosphatase type 5-like isoform X2
8906	AX-197963921	Apop_00987	NW_015441196	578853	C // G	8,45E-15	8,29E-13	outlier	0,11485	0,00013336	outlier	protein furry homolog-like
3750	AX-212305532	AP_var_0896	NW_015441093	508321	C // G	2,92E-05	0,000376016	outlier	0,026286	0,41108	n.s.	LOW QUALITY PROTEIN: serine-protein kinase ATM-like
3748	AX-197962952	Apop_00414	NW_015441093	477948	T // C	0,00013924	0,001488918	outlier	0,023013	0,57974	n.s.	involucrin-like isoform X1
4056	AX-198060431	Apop_00458	NW_015441097	89909	C // G	0,000104372	0,001168284	outlier	0,030767	0,33363	n.s.	uncharacterized protein LOC107332625
4058	AX-198060433	Apop_00459	NW_015441097	95715	T // C	0,000259349	0,002380888	outlier	0,023815	0,5191	n.s.	uncharacterized protein LOC107332625
19307	AX-212297124	AP_var_5780	NW_015442204	48206	A // G	0,01908261	0,06551693	n.s.	0,092301	0,010994	outlier	uncharacterized LOC107328743
4152	AX-198060454	Apop_00474	NW_015441100	254722	A // T	1,42E-05	0,000199461	outlier	0,028721	0,37209	n.s.	homeobox protein GBX-2-like isoform X2
4158	AX-198060459	Apop_00478	NW_015441100	555051	A // G	0,000138989	0,001488918	outlier	0,022258	0,62158	n.s.	zinc transporter Slc39a7-like
4380	AX-212302079	Apop_07866	NW_015441104	855381	T // G	7,28E-05	0,000852314	outlier	0,02596	0,46428	n.s.	melanopsin-like
4314	AX-197963093	Apop_00502	NW_015441104	571242	A // G	7,81E-05	0,000908529	outlier	0,028357	0,38564	n.s.	anion exchange protein 2-like
4576	AX-198060531	Apop_00524	NW_015441106	64359	T // G	1,48E-07	3,21E-06	outlier	0,051756	0,13281	n.s.	acyl-coenzyme A thioesterase 11-like
4574	AX-197963128	Apop_00523	NW_015441106	52937	A // G	6,62E-07	1,26E-05	outlier	0,044156	0,1723	n.s.	UPF0759 protein YunF-like isoform X2
4780	AX-197981516	Apop_02611	NW_015441108	427451	T // C	8,89E-07	1,66E-05	outlier	0,036921	0,26523	n.s.	microfibrillar-associated protein 1-like
4118	AX-198060434	Apop_00460	NW_015441098	1004157	T // C	5,42E-09	1,63E-07	outlier	0,079264	0,022949	outlier	protein saal1-like
4842	AX-198060565	Apop_00547	NW_015441110	840170	T // C	0,000514863	0,004166979	outlier	0,02466	0,50722	n.s.	phthiocerol synthesis polyketide synthase type I PpsC-like
5110	AX-212292589	AP_var_1239	NW_015441114	326189	A // G	0,000267513	0,002438295	outlier	0,023117	0,5531	n.s.	uncharacterized protein LOC107334247
5124	AX-212302164	Apop_07951	NW_015441115	228378	A // C	0,000196811	0,001936826	outlier	0,023547	0,54925	n.s.	disks large homolog 1-like
5995	AX-212302303	Apop_08090	NW_015441133	858542	A // T	2,48E-06	4,06E-05	outlier	0,11473	0	outlier	protocadherin-like protein
5652	AX-198060695	Apop_00622	NW_015441126	486992	A // G	1,90E-09	6,45E-08	outlier	0,027567	0,38175	n.s.	WD repeat-containing protein 36-like
5648	AX-198060689	Apop_00619	NW_015441126	393217	T // C	5,36E-08	1,32E-06	outlier	0,055646	0,12456	n.s.	FGGY carbohydrate kinase domain-containing protein-like isoform X1
6136	AX-198060819	Apop_00693	NW_015441136	903063	C // G	0,000358895	0,003080513	outlier	0,026647	0,40021	n.s.	potassium voltage-gated channel subfamily H member 7-like
14413	AX-212295289	AP_var_3942	NW_015441418	348961	C // G	0,02184985	0,07248252	n.s.	0,084605	0,020368	outlier	uncharacterized LOC107349419
6734	AX-197963530	Apop_00758	NW_015441148	729055	A // T	2,09E-08	5,88E-07	outlier	0,061588	0,085708	n.s.	protein unc-93 homolog A-like

15785	AX-212303784	Apop_09554	NW_015441499	55101	C // G	0,001999489	0,01246641	n.s.	0,091317	0,0010002	outlier	uncharacterized protein PB18E9.04c-like
4800	AX-212298300	Apop_04100	NW_015441108	870486	T // C	2,08E-11	1,14E-09	outlier	0,087523	0,014324	outlier	uncharacterized protein LOC107333733
6812	AX-212315746	Apop_08228	NW_015441151	220752	A // C	2,09E-08	5,88E-07	outlier	0,037689	0,20881	n.s.	mu-type opioid receptor-like
7130	AX-198060980	Apop_00798	NW_015441157	14621	A // G	0,000245868	0,002275233	outlier	0,02258	0,58255	n.s.	LOW QUALITY PROTEIN: AP-2 complex subunit alpha-2-like
7138	AX-212293132	AP_var_1782	NW_015441157	253307	T // G	6,26E-08	1,51E-06	outlier	0,055253	0,10363	n.s.	serine-rich adhesin for platelets-like isoform X3
7270	AX-212315842	Apop_08324	NW_015441160	261580	T // C	5,12E-07	9,91E-06	outlier	0,07004	0,053348	n.s.	annexin A6-like
7476	AX-197963665	Apop_00837	NW_015441163	470719	T // C	0,000307531	0,002731511	outlier	0,022064	0,63303	n.s.	kinesin-like protein KIF14
13865	AX-198062401	Apop_01688	NW_015441379	114441	A // G	8,23E-07	1,55E-05	outlier	0,15389	0	outlier	CCR4-NOT transcription complex subunit 1-like
9414	AX-197964047	Apop_01058	NW_015441211	550174	A // G	1,18E-11	6,72E-10	outlier	0,09697	0,0029896	outlier	protein yippee-like 2 isoform X2
7460	AX-212306503	AP_var_1867	NW_015441163	310902	T // C	0,00086075	0,006448323	outlier	0,03454	0,29273	n.s.	spliceosome RNA helicase Ddx39b
7676	AX-198061091	Apop_00867	NW_015441168	480392	A // G	0,000198781	0,00194642	outlier	0,024709	0,48923	n.s.	uncharacterized protein LOC107338309 isoform X3
8370	AX-197963848	Apop_00943	NW_015441183	272527	A // G	0,000314373	0,002772974	outlier	0,024586	0,52913	n.s.	uncharacterized protein LOC107339244
8976	AX-212293622	AP_var_2275	NW_015441198	529598	T // C	8,49E-05	0,000979269	outlier	0,0225	0,57808	n.s.	Down syndrome cell adhesion molecule-like protein 1 homolog
1472	AX-212312472	Apop_04986	NW_015441063	1341949	C // G	0,000203379	0,00194642	outlier	0,076261	0,074096	n.s.	uncharacterized protein LOC107340032
1440	AX-212314340	Apop_06856	NW_015441063	1258402	T // G	0,000203379	0,00194642	outlier	0,080045	0,063467	n.s.	uncharacterized protein LOC107340588
1476	AX-212299205	Apop_05006	NW_015441063	1369267	T // C	0,000203379	0,00194642	outlier	0,075139	0,08282	n.s.	uncharacterized protein LOC107340661
1474	AX-212312490	Apop_05004	NW_015441063	1368664	C // G	0,000203379	0,00194642	outlier	0,074057	0,087426	n.s.	uncharacterized protein LOC107340661
1478	AX-212312494	Apop_05008	NW_015441063	1369414	T // C	0,000271865	0,002460387	outlier	0,063406	0,11433	n.s.	uncharacterized protein LOC107340661
9518	AX-212293784	AP_var_2437	NW_015441215	130118	A // C	0,000323164	0,00283775	outlier	0,021782	0,65591	n.s.	arylsulfatase H-like
9638	AX-212302849	Apop_08619	NW_015441219	173862	T // G	1,51E-06	2,61E-05	outlier	0,037681	0,27801	n.s.	DNA repair protein REV1-like
9964	AX-212298563	Apop_04363	NW_015441226	197980	T // C	1,94E-07	4,00E-06	outlier	0,11195	0,0018189	outlier	CCR4-NOT transcription complex subunit 2-like
9676	AX-197964123	Apop_01103	NW_015441222	192844	A // G	0,001436351	0,009546159	outlier	0,020253	0,88987	n.s.	importin-7-like
9670	AX-212302858	Apop_08628	NW_015441222	65461	A // G	0,000449575	0,003733291	outlier	0,021068	0,84061	n.s.	centromere-associated protein E-like
10508	AX-197964334	Apop_01230	NW_015441253	123707	T // C	5,05E-05	0,000624202	outlier	0,061185	0,11647	n.s.	frizzled-4-like
10842	AX-197964413	Apop_01276	NW_015441267	291352	T // C	0,00010208	0,001145978	outlier	0,023015	0,55036	n.s.	tumor protein p63-regulated gene 1-like protein
10984	AX-198061793	Apop_01309	NW_015441272	68971	A // G	0,00053644	0,004307077	outlier	0,022171	0,61484	n.s.	beta-mannosidase-like
11227	AX-198078939	Apop_02812	NW_015441279	32929	C // G	0,002675024	0,01575452	n.s.	0,070896	0,022216	outlier	uncharacterized protein LOC107344142
11078	AX-197964482	Apop_01312	NW_015441273	219997	T // C	0,000159014	0,001633826	outlier	0,023118	0,54219	n.s.	uncharacterized protein LOC107343867
11084	AX-212316383	Apop_08865	NW_015441273	245187	A // G	0,000455113	0,003762953	outlier	0,021057	0,86419	n.s.	epithelial splicing regulatory protein 1-like
11794	AX-197981930	Apop_02862	NW_015441302	480895	T // C	0,000161754	0,001633826	outlier	0,024756	0,49033	n.s.	E3 ubiquitin-protein ligase TRIM71-like
11170	AX-198061820	Apop_01326	NW_015441277	469588	A // T	1,25E-11	7,05E-10	outlier	0,076434	0,018659	outlier	stimulated by retinoic acid gene 6 protein homolog
11792	AX-212303219	Apop_08989	NW_015441302	479377	A // G	0,000240629	0,002235844	outlier	0,024631	0,49894	n.s.	E3 ubiquitin-protein ligase TRIM71-like
11900	AX-198061922	Apop_01387	NW_015441303	259429	T // C	0,001404864	0,009449458	outlier	0,021108	0,76894	n.s.	UHRF1-binding protein 1-like
12008	AX-197964664	Apop_01414	NW_015441307	458765	T // C	0,000392318	0,00333746	outlier	0,02779	0,39915	n.s.	polycystic kidney disease protein 1-like 2

12188	AX-212300269	Apop_06059	NW_015441314	366949	C // G	0,000438438	0,003664655	outlier	0,064922	0,093778	n.s.	cell wall protein DAN4-like isoform X2
12654	AX-197964878	Apop_01534	NW_015441337	414193	T // C	1,29E-07	2,85E-06	outlier	0,027566	0,40699	n.s.	inhibitor of nuclear factor kappa-B kinase subunit alpha-like
12732	AX-212303362	Apop_09132	NW_015441347	8143	A // G	0,000482176	0,003952564	outlier	0,022106	0,58417	n.s.	tuberin-like
376	AX-198059710	Apop_00012	NW_015441057	1628659	T // C	0,000418818	0,003531509	outlier	0,022193	0,63803	n.s.	uncharacterized protein LOC107346978 isoform X2
12828	AX-197964944	Apop_01576	NW_015441352	33321	A // G	1,14E-06	2,03E-05	outlier	0,039999	0,20716	n.s.	two pore potassium channel protein sup-9-like
12922	AX-198062249	Apop_01586	NW_015441354	124140	T // C	3,37E-05	0,000429005	outlier	0,023112	0,56524	n.s.	transcriptional regulator Myc-B-like
12952	AX-197964970	Apop_01594	NW_015441355	242451	A // G	0,000403503	0,003425	outlier	0,027143	0,41561	n.s.	uncharacterized protein LOC107347266
13088	AX-212308226	AP_var_3593	NW_015441362	293866	A // G	0,000287655	0,00258495	outlier	0,022243	0,58939	n.s.	collagen alpha-4(VI) chain-like
13800	AX-198062399	Apop_01686	NW_015441378	215132	T // C	0,00087048	0,006495794	outlier	0,022038	0,69176	n.s.	U1 small nuclear ribonucleoprotein 70 kDa-like
13782	AX-197938701	Afix_00627	NW_015441378	133682	A // C	0,001040636	0,007363636	outlier	0,037394	0,26016	n.s.	uncharacterized protein LOC107348149
13868	AX-197965127	Apop_01690	NW_015441379	236047	A // T	4,48E-05	0,000560351	outlier	0,025559	0,45674	n.s.	tyrosine aminotransferase-like
14062	AX-212303567	Apop_09337	NW_015441395	222031	A // G	0,001339129	0,009149608	outlier	0,02059	0,88759	n.s.	uncharacterized LOC107348698
14058	AX-212316853	Apop_09335	NW_015441395	174121	A // G	0,000592458	0,004657136	outlier	0,02084	0,8894	n.s.	uncharacterized protein LOC107348725 isoform X2
2847	AX-197962777	Apop_00309	NW_015441079	444425	A // G	0,002263656	0,01393191	n.s.	0,14709	0	outlier	uncharacterized protein LOC107328618
14196	AX-198062483	Apop_01741	NW_015441404	247864	A // G	0,000363461	0,003112729	outlier	0,022027	0,60456	n.s.	methyltransferase-like protein 9
14192	AX-198062481	Apop_01739	NW_015441404	113290	T // G	0,000415512	0,003511366	outlier	0,021739	0,65957	n.s.	uncharacterized protein LOC107348982 isoform X3
14198	AX-212303612	Apop_09382	NW_015441404	270750	T // C	3,70E-06	5,92E-05	outlier	0,027711	0,37324	n.s.	interferon-inducible GTPase 1-like
14332	AX-212295241	AP_var_3894	NW_015441411	261314	T // C	7,58E-06	0,000110776	outlier	0,026497	0,41511	n.s.	monocarboxylate transporter 10-like
14624	AX-212308637	AP_var_4004	NW_015441435	153906	T // C	5,09E-07	9,90E-06	outlier	0,029344	0,35124	n.s.	transmembrane anterior posterior transformation protein 1 homolog
6710	AX-212292986	AP_var_1636	NW_015441148	13483	A // T	4,36E-08	1,12E-06	outlier	0,081681	0,014608	outlier	centromere protein F-like
11280	AX-197981827	Apop_02799	NW_015441279	265897	C // G	1,55E-10	6,80E-09	outlier	0,12572	6,67E-05	outlier	organic cation transporter protein-like
14710	AX-197965324	Apop_01812	NW_015441438	166874	A // G	0,000169207	0,001700141	outlier	0,038816	0,21613	n.s.	uncharacterized protein LOC107349976 isoform X1
1898	AX-198038148	Afix_02301	NW_015441069	1282640	T // G	0,000333638	0,002886725	outlier	0,020664	0,88725	n.s.	neuronal acetylcholine receptor subunit alpha-10-like isoform X2
15050	AX-197965373	Apop_01842	NW_015441449	210434	T // G	0,000111826	0,00123725	outlier	0,021087	0,75559	n.s.	lysyl oxidase homolog 4-like
1894	AX-198060011	Apop_00197	NW_015441069	1273317	A // G	2,62E-05	0,000342842	outlier	0,024381	0,53157	n.s.	neuropeptide FF receptor 2-like
15616	AX-212298598	Apop_04398	NW_015441494	114803	T // C	2,14E-08	5,99E-07	outlier	0,05681	0,10104	n.s.	trichohyalin-like isoform X3
15614	AX-197965486	Apop_01909	NW_015441494	112470	C // G	2,48E-10	1,07E-08	outlier	0,070234	0,047632	outlier	trichohyalin-like isoform X3
15790	AX-212303788	Apop_09558	NW_015441499	83722	A // T	1,78E-13	1,42E-11	outlier	0,026932	0,39112	n.s.	pachytene checkpoint protein 2 homolog
16048	AX-197965567	Apop_01959	NW_015441517	262546	A // T	5,70E-05	0,00069312	outlier	0,022986	0,55768	n.s.	uncharacterized protein LOC107352106 isoform X2
16072	AX-212317125	Apop_09607	NW_015441518	282168	A // G	0,001262487	0,008755451	outlier	0,020955	0,88891	n.s.	inositol phosphorylceramide synthase catalytic subunit aur1-like
16304	AX-197965606	Apop_01986	NW_015441538	193078	T // C	5,13E-09	1,56E-07	outlier	0,041276	0,20057	n.s.	adrenocorticotropic hormone receptor-like
16576	AX-198062974	Apop_02043	NW_015441569	98253	T // C	3,21E-12	2,08E-10	outlier	0,062024	0,091423	n.s.	leucine--tRNA ligase, cytoplasmic-like isoform X3
16680	AX-212303983	Apop_09753	NW_015441583	65594	C // G	9,68E-07	1,78E-05	outlier	0,031018	0,31791	n.s.	E3 ubiquitin-protein ligase RNF213-like

2248	AX-198060074	Apop_00238	NW_015441073	223836	T // C	9,30E-06	0,00013378	outlier	0,023622	0,54103	n.s.	melanocyte-stimulating hormone receptor-like
11228	AX-197981850	Apop_02813	NW_015441279	33090	C // G	2,97E-13	2,28E-11	outlier	0,071786	0,029805	outlier	uncharacterized protein LOC107344142
16842	AX-197965762	Apop_02081	NW_015441598	110091	A // G	1,54E-07	3,30E-06	outlier	0,049431	0,13732	n.s.	kelch-like protein diablo
17072	AX-212304089	Apop_09859	NW_015441628	116084	T // G	0,000143369	0,001528801	outlier	0,023463	0,53457	n.s.	disrupted in renal carcinoma protein 2-like isoform X1
17928	AX-197966019	Apop_02225	NW_015441749	172505	A // G	0,000324855	0,002845761	outlier	0,02375	0,51291	n.s.	uncharacterized protein LOC107356543
17932	AX-198063268	Apop_02233	NW_015441751	53822	A // G	0,000112781	0,001244221	outlier	0,025944	0,45928	n.s.	mRNA-decapping enzyme 1B-like
18012	AX-198063284	Apop_02243	NW_015441774	165647	T // C	0,000410855	0,003479688	outlier	0,023066	0,55119	n.s.	multidrug and toxin extrusion protein 2-like
18134	AX-198063309	Apop_02259	NW_015441802	154092	T // C	0,000160511	0,001633826	outlier	0,033831	0,28084	n.s.	hepatocyte nuclear factor 1-beta-A-like isoform X1
18210	AX-197966110	Apop_02275	NW_015441816	34079	T // C	3,97E-07	7,96E-06	outlier	0,050377	0,14185	n.s.	protein FAM26E-like
18262	AX-197966118	Apop_02279	NW_015441817	14551	C // G	0,0005333	0,004298023	outlier	0,022862	0,58646	n.s.	structural maintenance of chromosomes protein 1A-like
2538	AX-198060095	Apop_00252	NW_015441076	1087807	A // G	0,000160649	0,001633826	outlier	0,024824	0,50208	n.s.	uncharacterized protein PB18E9.04c-like
18440	AX-197966148	Apop_02295	NW_015441856	88058	T // C	1,24E-09	4,34E-08	outlier	0,029266	0,33809	n.s.	exportin-T-like
18622	AX-198063385	Apop_02309	NW_015441897	26703	T // G	4,42E-06	7,05E-05	outlier	0,024306	0,47828	n.s.	uncharacterized protein LOC107358496
11283	AX-197981829	Apop_02800	NW_015441279	270531	T // C	0,004213056	0,0221847	n.s.	0,13831	0	outlier	organic cation transporter protein-like
15615	AX-197965487	Apop_01910	NW_015441494	113291	A // G	0,01144049	0,04674076	n.s.	0,071129	0,049001	outlier	trichohyalin-like isoform X3
18700	AX-212317705	Apop_10188	NW_015441914	91550	T // C	0,001466976	0,009699784	outlier	0,024547	0,49646	n.s.	protein FAM214A-like
8521	AX-212316000	Apop_08482	NW_015441188	2721	A // G	0,002216824	0,0137098	n.s.	0,094728	0,00030498	outlier	#N/D
14033	AX-197982160	Apop_02992	NW_015441393	64062	T // C	0,0111096	0,04558348	n.s.	0,10105	0,00021823	outlier	#N/D
15795	AX-212303793	Apop_09563	NW_015441499	208905	A // T	0,002659663	0,01568818	n.s.	0,088775	0,0017378	outlier	#N/D
5107	AX-212292586	AP_var_1236	NW_015441114	259641	A // G	0,023705	0,07620884	n.s.	0,085965	0,0045993	outlier	#N/D
7305	AX-212302571	Apop_08341	NW_015441161	331568	T // C	0,01174906	0,04750375	n.s.	0,11203	0,001106	outlier	#N/D
7143	AX-198078703	Apop_02655	NW_015441157	457277	C // G	0,01410316	0,05403354	n.s.	0,081147	0,0039646	outlier	#N/D
143	AX-197981168	Apop_02394	NW_015441057	916328	C // G	0,008572371	0,03737637	n.s.	0,093504	0,0007104	outlier	#N/D
1345	AX-198059861	Apop_00105	NW_015441062	1032034	T // C	0,006826498	0,03163797	n.s.	0,082953	0,0097176	outlier	#N/D
12189	AX-212312102	Apop_04616	NW_015441314	367510	A // T	0,05274298	0,1301839	n.s.	0,073238	0,031933	outlier	#N/D
8055	AX-212293377	AP_var_2030	NW_015441177	245662	T // C	0,01463635	0,05560501	n.s.	0,087197	0,0067119	outlier	#N/D
9417	AX-197964051	Apop_01060	NW_015441211	552329	A // G	0,006712988	0,03130137	n.s.	0,096638	0,0024543	outlier	#N/D
9423	AX-212300427	Apop_06217	NW_015441211	572850	C // G	0,006712988	0,03130137	n.s.	0,096746	0,0026496	outlier	#N/D
961	AX-212291473	AP_var_0114	NW_015441060	24336	A // T	0,006258319	0,029650841	n.s.	0,087188	0,0056233	outlier	#N/D
12453	AX-212316589	Apop_09071	NW_015441326	315279	A // G	0,01787701	0,06238484	n.s.	0,066252	0,045453	outlier	#N/D
8595	AX-197963894	Apop_00971	NW_015441190	288398	T // G	0,02513513	0,07945619	n.s.	0,077896	0,019325	outlier	#N/D
18665	AX-212317696	Apop_10179	NW_015441902	51385	A // T	0,001565966	0,01017789	n.s.	0,085291	0,021103	outlier	#N/D
12181	AX-212300211	Apop_06001	NW_015441314	362772	T // G	0,06692118	0,1522194	n.s.	0,078628	0,027317	outlier	#N/D
19639	AX-212298926	Apop_04726	NW_015442919	4784	A // G	0,06692118	0,1522194	n.s.	0,075446	0,036273	outlier	#N/D

12183	AX-212310733	Apop_03247	NW_015441314	363274	A // T	0,06692118	0,1522194	n.s.	0,075974	0,035532	outlier	#N/D
17163	AX-212304101	Apop_09871	NW_015441636	89790	T // C	0,006908626	0,03182595	n.s.	0,069786	0,034794	outlier	#N/D
5607	AX-197981550	Apop_02632	NW_015441124	620028	A // G	0,02157988	0,07164903	n.s.	0,07672	0,045027	outlier	#N/D
17729	AX-212317514	Apop_09996	NW_015441710	65636	A // G	0,02211163	0,07309755	n.s.	0,087238	0,014901	outlier	#N/D
5407	AX-198060618	Apop_00578	NW_015441117	797988	A // C	0,009640662	0,04100672	n.s.	0,069436	0,04088	outlier	#N/D
15833	AX-198062802	Apop_01933	NW_015441503	187382	T // C	0,02667388	0,08210105	n.s.	0,094629	0,025146	outlier	#N/D
1419	AX-197981197	Apop_02409	NW_015441063	1017097	A // T	0,04373447	0,1164275	n.s.	0,082934	0,012102	outlier	#N/D
5411	AX-197963227	Apop_00582	NW_015441117	820279	T // C	0,02013686	0,067959	n.s.	0,079545	0,011268	outlier	#N/D
10115	AX-212302926	Apop_08696	NW_015441237	118552	A // C	0,02373179	0,07620884	n.s.	0,088598	0,035901	outlier	#N/D
19465	AX-212304605	Apop_10375	NW_015442377	32655	T // C	0,02884143	0,08686841	n.s.	0,086033	0,0090504	outlier	#N/D
7273	AX-198061002	Apop_00811	NW_015441160	290637	T // C	0,01552397	0,05755425	n.s.	0,091727	0,0033828	outlier	#N/D
3381	AX-197962893	Apop_00381	NW_015441089	534515	T // C	0,009832419	0,04163462	n.s.	0,076533	0,049929	outlier	#N/D
2849	AX-212305279	AP_var_0634	NW_015441079	467412	A // G	0,01411292	0,05403354	n.s.	0,0818	0,032993	outlier	#N/D
5973	AX-197963372	Apop_00665	NW_015441133	162420	A // T	0,03850881	0,1068266	n.s.	0,0838	0,018	outlier	#N/D
7271	AX-212315843	Apop_08325	NW_015441160	276828	A // G	0,05101932	0,1284937	n.s.	0,074922	0,03892	outlier	#N/D
12495	AX-212316604	Apop_09086	NW_015441330	248575	T // G	0,0172991	0,06127237	n.s.	0,083048	0,016413	outlier	#N/D
17731	AX-212317515	Apop_09997	NW_015441710	74573	T // G	0,2872043	0,3722931	n.s.	0,11605	0,0082359	outlier	#N/D
12397	AX-212294687	AP_var_3340	NW_015441323	266307	T // C	0,01086672	0,04487553	n.s.	0,10156	0,0078465	outlier	#N/D
12399	AX-212307975	AP_var_3342	NW_015441323	283170	A // G	0,01496758	0,05646003	n.s.	0,10149	0,0094745	outlier	#N/D
2995	AX-212292028	AP_var_0670	NW_015441081	821877	T // C	0,01318791	0,05151583	n.s.	0,078672	0,034065	outlier	#N/D
145	AX-198078300	Apop_02396	NW_015441057	952586	A // G	5,68E-06	8,66E-05	outlier	0,14224	0	outlier	#N/D
146	AX-197981176	Apop_02398	NW_015441057	966667	T // C	3,73E-34	3,57E-31	outlier	0,15066	0	outlier	#N/D
148	AX-198078307	Apop_02400	NW_015441057	976090	T // G	6,97E-30	3,34E-27	outlier	0,14143	0	outlier	#N/D
12902	AX-212316660	Apop_09142	NW_015441353	352310	C // G	3,14E-53	1,20E-49	outlier	0,18121	0	outlier	#N/D
15788	AX-212303787	Apop_09557	NW_015441499	78647	A // G	5,39E-37	6,88E-34	outlier	0,19671	0	outlier	#N/D
2832	AX-197942719	Afix_03180	NW_015441079	220405	C // G	4,86E-06	7,57E-05	outlier	0,093406	0,017044	outlier	#N/D
2836	AX-198039559	Afix_03186	NW_015441079	237170	T // C	1,83E-07	3,87E-06	outlier	0,10395	0,0044379	outlier	#N/D
12824	AX-197954637	Afix_10691	NW_015441352	28578	T // C	4,21E-14	3,66E-12	outlier	0,095091	0,0015014	outlier	#N/D
2840	AX-212291989	AP_var_0630	NW_015441079	316141	C // G	7,99E-09	2,32E-07	outlier	0,11313	0,00043244	outlier	#N/D
9405	AX-212306351	AP_var_1715	NW_015441211	458272	T // C	0,000788137	0,005950925	outlier	0,11841	0,00030498	outlier	#N/D
12182	AX-212306884	AP_var_2251	NW_015441314	363137	A // C	4,21E-07	8,35E-06	outlier	0,073421	0,025873	outlier	#N/D
15996	AX-212309034	AP_var_4404	NW_015441515	165253	A // T	8,54E-05	0,000981268	outlier	0,094818	0,035164	outlier	#N/D
17999	AX-212296530	AP_var_5186	NW_015441773	54242	T // C	0,000260517	0,002385888	outlier	0,098752	0,00033975	outlier	#N/D
18666	AX-212296772	AP_var_5428	NW_015441902	53282	T // C	2,52E-15	2,68E-13	outlier	0,082244	0,030163	outlier	#N/D
1488	AX-198059946	Apop_00156	NW_015441064	884998	A // G	2,26E-21	5,76E-19	outlier	0,12151	3,16E-05	outlier	#N/D
2586	AX-198060132	Apop_00273	NW_015441077	28479	T // C	1,32E-25	4,59E-23	outlier	0,10107	0,00025867	outlier	#N/D

2848	AX-198060200	Apop_00310	NW_01544107!	449882	A // C	8,40E-12	5,02E-10	outlier	0,14373	0,00021823	outlier	#N/D
4110	AX-198060444	Apop_00467	NW_01544109l	781826	T // C	4,73E-08	1,20E-06	outlier	0,090692	0,0072874	outlier	#N/D
5406	AX-197963218	Apop_00577	NW_01544111	787901	T // C	4,11E-11	2,01E-09	outlier	0,071781	0,030877	outlier	#N/D
12003	AX-198061965	Apop_01410	NW_01544130l	382444	T // G	3,04E-06	4,90E-05	outlier	0,10966	0	outlier	#N/D
12138	AX-197964695	Apop_01431	NW_01544131	383905	T // C	1,01E-06	1,83E-05	outlier	0,080643	0,01966	outlier	#N/D
14009	AX-197965164	Apop_01713	NW_01544138l	288402	A // C	0,000583909	0,004599386	outlier	0,11378	0	outlier	#N/D
14346	AX-197965244	Apop_01763	NW_01544141	260185	T // C	2,01E-22	5,93E-20	outlier	0,10396	0,00033975	outlier	#N/D
17998	AX-197966049	Apop_02242	NW_01544177	48608	C // G	2,24E-16	2,95E-14	outlier	0,097353	0,00086359	outlier	#N/D
19109	AX-197966253	Apop_02351	NW_01544207	58087	C // G	5,92E-05	0,000714458	outlier	0,11256	6,67E-05	outlier	#N/D
8899	AX-198078822	Apop_02733	NW_01544119l	557406	A // C	0,001475024	0,009718819	outlier	0,10871	0,00021823	outlier	#N/D
8900	AX-197981720	Apop_02735	NW_01544119l	560200	T // G	1,77E-15	2,00E-13	outlier	0,10849	0,00047438	outlier	#N/D
8901	AX-197981722	Apop_02736	NW_01544119l	560352	A // G	0,001475024	0,009718819	outlier	0,10736	0,00043244	outlier	#N/D
8904	AX-198078835	Apop_02742	NW_01544119l	576004	A // C	8,71E-07	1,63E-05	outlier	0,079479	0,038541	outlier	#N/D
11206	AX-198078879	Apop_02774	NW_01544127!	10893	A // T	2,01E-14	1,83E-12	outlier	0,075604	0,015804	outlier	#N/D
11288	AX-198078919	Apop_02801	NW_01544127!	279230	A // T	2,66E-12	1,76E-10	outlier	0,13392	3,16E-05	outlier	#N/D
11220	AX-197981831	Apop_02802	NW_01544127!	29438	A // G	7,12E-16	8,52E-14	outlier	0,081089	0,0076614	outlier	#N/D
11221	AX-198078923	Apop_02803	NW_01544127!	29562	T // C	0,000917557	0,006729044	outlier	0,081463	0,0052704	outlier	#N/D
11222	AX-197981837	Apop_02805	NW_01544127!	30188	T // C	1,09E-16	1,61E-14	outlier	0,080788	0,0065265	outlier	#N/D
11223	AX-198078929	Apop_02807	NW_01544127!	31332	C // G	0,000917557	0,006729044	outlier	0,080297	0,008039	outlier	#N/D
11224	AX-198078931	Apop_02808	NW_01544127!	32028	T // C	1,09E-16	1,61E-14	outlier	0,07948	0,007475	outlier	#N/D
11205	AX-198078988	Apop_02840	NW_01544127!	9180	A // G	0,00066622	0,00511737	outlier	0,081698	0,0049239	outlier	#N/D
11214	AX-212316417	Apop_08899	NW_01544127!	19882	T // C	2,70E-10	1,13E-08	outlier	0,061592	0,061783	n.s.	#N/D
13738	AX-197982124	Apop_02972	NW_01544137	145701	A // G	2,45E-13	1,91E-11	outlier	0,10292	0,0023578	outlier	#N/D
16838	AX-212304012	Apop_09782	NW_01544159l	85215	A // G	2,39E-08	6,59E-07	outlier	0,065182	0,054834	n.s.	#N/D
13740	AX-198079232	Apop_02973	NW_01544137	157621	A // T	1,30E-10	5,78E-09	outlier	0,095425	0,010213	outlier	#N/D
7142	AX-212310996	Apop_03510	NW_01544115	447358	T // C	4,28E-19	8,63E-17	outlier	0,12196	3,16E-05	outlier	#N/D
17377	AX-212311312	Apop_03826	NW_01544165l	20136	A // G	7,85E-05	0,000910862	outlier	0,11249	0,00013336	outlier	#N/D
9422	AX-212311489	Apop_04003	NW_01544121	572305	A // G	1,18E-11	6,72E-10	outlier	0,096677	0,0027538	outlier	#N/D
11232	AX-198078970	Apop_02830	NW_01544127!	57607	T // C	7,65E-13	5,74E-11	outlier	0,058747	0,082239	n.s.	#N/D
14106	AX-198062463	Apop_01727	NW_01544139!	61217	A // G	1,37E-08	3,94E-07	outlier	0,058365	0,089703	n.s.	#N/D
9416	AX-212298444	Apop_04244	NW_01544121	551925	T // C	6,77E-12	4,11E-10	outlier	0,10114	0,0014329	outlier	#N/D
9410	AX-212311961	Apop_04475	NW_01544121	547031	T // C	7,85E-08	1,83E-06	outlier	0,08473	0,012657	outlier	#N/D
12180	AX-212312181	Apop_04695	NW_01544131	362263	A // G	4,54E-06	7,16E-05	outlier	0,076169	0,032641	outlier	#N/D
15328	AX-212313261	Apop_05764	NW_01544147l	204386	T // C	4,90E-16	6,25E-14	outlier	0,11582	0	outlier	#N/D
11204	AX-197981891	Apop_02838	NW_01544127!	8502	C // G	2,05E-09	6,81E-08	outlier	0,05202	0,10858	n.s.	#N/D
9306	AX-212313323	Apop_05826	NW_01544120l	498891	T // C	2,77E-09	9,06E-08	outlier	0,10642	0,010463	outlier	#N/D

12184	AX-212300119	Apop_05909	NW_015441314	363922	T // C	4,54E-06	7,16E-05	outlier	0,07721	0,027673	outlier	#N/D
19640	AX-212313665	Apop_06168	NW_015442919	5280	A // G	2,01E-06	3,35E-05	outlier	0,076258	0,020733	outlier	#N/D
19638	AX-212314176	Apop_06692	NW_015442919	2496	A // T	2,99E-05	0,000384714	outlier	0,071851	0,049001	outlier	#N/D
144	AX-212301499	Apop_07311	NW_015441057	950956	T // C	8,46E-14	7,20E-12	outlier	0,09329	0,00078516	outlier	#N/D
151	AX-212314790	Apop_07314	NW_015441057	991822	A // C	0,000227945	0,002133526	outlier	0,12201	0	outlier	#N/D
1338	AX-212314918	Apop_07417	NW_015441062	1006701	A // C	1,58E-09	5,45E-08	outlier	0,07917	0,02	outlier	#N/D
16492	AX-197965669	Apop_02025	NW_015441556	160798	C // G	1,10E-07	2,49E-06	outlier	0,058403	0,083398	n.s.	#N/D
3583	AX-212315230	Apop_07729	NW_015441091	524487	A // G	0,001409459	0,009449458	outlier	0,084971	0,0058012	outlier	#N/D
6758	AX-212302430	Apop_08200	NW_015441148	828299	T // C	4,11E-11	2,01E-09	outlier	0,070928	0,031581	outlier	#N/D
7268	AX-212302551	Apop_08321	NW_015441160	188497	A // C	1,79E-16	2,47E-14	outlier	0,10812	0,00025867	outlier	#N/D
10892	AX-212316332	Apop_08814	NW_015441267	447652	T // C	3,41E-10	1,39E-08	outlier	0,047725	0,14566	n.s.	#N/D
10886	AX-212303042	Apop_08812	NW_015441267	423649	A // G	3,41E-10	1,39E-08	outlier	0,048024	0,14642	n.s.	#N/D
9406	AX-212316104	Apop_08586	NW_015441211	459855	A // G	1,27E-13	1,04E-11	outlier	0,11353	0,0013651	outlier	#N/D
11074	AX-212303091	Apop_08861	NW_015441273	189154	T // C	2,24E-11	1,19E-09	outlier	0,075178	0,028027	outlier	#N/D
18730	AX-212304431	Apop_10201	NW_015441931	138560	T // G	0,00012365	0,001344751	outlier	0,059206	0,14489	n.s.	#N/D
4396	AX-212302080	Apop_07867	NW_015441104	895958	A // C	7,15E-08	1,71E-06	outlier	0,058057	0,1093	n.s.	#N/D
9772	AX-212302873	Apop_08643	NW_015441224	50693	C // G	5,16E-07	9,93E-06	outlier	0,036352	0,23515	n.s.	#N/D
15852	AX-212300790	Apop_06593	NW_015441504	198362	A // T	1,92E-09	6,45E-08	outlier	0,063069	0,079359	n.s.	#N/D
11180	AX-212303120	Apop_08890	NW_015441278	220143	A // G	5,44E-10	2,07E-08	outlier	0,078991	0,038159	outlier	#N/D
17448	AX-212296347	AP_var_5003	NW_015441663	107893	A // C	2,07E-07	4,26E-06	outlier	0,051254	0,1426	n.s.	#N/D
14341	AX-212303631	Apop_09401	NW_015441413	210201	T // C	9,91E-05	0,001122258	outlier	0,10324	0,00021823	outlier	#N/D
4268	AX-212315351	Apop_07850	NW_015441104	31836	T // G	1,34E-07	2,95E-06	outlier	0,033512	0,26738	n.s.	#N/D
8266	AX-212315978	Apop_08460	NW_015441181	462154	A // G	0,000257646	0,002370942	outlier	0,063129	0,097957	n.s.	#N/D
15454	AX-212317044	Apop_09526	NW_015441479	56144	T // C	5,65E-11	2,67E-09	outlier	0,080962	0,0072874	outlier	#N/D
5620	AX-212302244	Apop_08031	NW_015441125	25339	T // C	5,98E-05	0,000719735	outlier	0,056718	0,10433	n.s.	#N/D
18984	AX-212317774	Apop_10257	NW_015442016	76448	A // G	6,23E-09	1,86E-07	outlier	0,038308	0,22095	n.s.	#N/D
15786	AX-212317073	Apop_09555	NW_015441499	63304	C // G	7,30E-11	3,33E-09	outlier	0,072686	0,024782	outlier	#N/D
9536	AX-212316116	Apop_08598	NW_015441215	283074	A // G	2,34E-05	0,000315341	outlier	0,032646	0,30574	n.s.	#N/D
16972	AX-212304063	Apop_09833	NW_015441618	226165	A // G	1,02E-07	2,36E-06	outlier	0,076661	0,044605	outlier	#N/D
10214	AX-212316232	Apop_08714	NW_015441240	192312	A // G	0,000122914	0,001344385	outlier	0,06149	0,10168	n.s.	#N/D
2406	AX-198060081	Apop_00242	NW_015441074	1194496	A // C	1,44E-06	2,54E-05	outlier	0,038057	0,23671	n.s.	#N/D
16992	AX-212317356	Apop_09838	NW_015441623	21001	T // G	1,92E-14	1,79E-12	outlier	0,093288	0,00052511	outlier	#N/D
5526	AX-212315511	Apop_08010	NW_015441122	642177	A // G	2,09E-10	9,10E-09	outlier	0,038488	0,24366	n.s.	#N/D
14982	AX-212317000	Apop_09482	NW_015441448	269046	A // G	1,65E-05	0,000227657	outlier	0,058624	0,094366	n.s.	#N/D
12968	AX-212303404	Apop_09174	NW_015441356	399749	A // G	9,27E-07	1,72E-05	outlier	0,030304	0,31991	n.s.	#N/D
13832	AX-212303518	Apop_09288	NW_015441378	344168	A // C	2,18E-07	4,46E-06	outlier	0,033092	0,30438	n.s.	#N/D

4372	AX-212315365	Apop_07864	NW_015441104	836572	T // G	6,28E-06	9,35E-05	outlier	0,039877	0,21371	n.s.	#N/D
17048	AX-197965829	Apop_02116	NW_015441625	156366	T // G	2,58E-05	0,000341459	outlier	0,040392	0,18645	n.s.	#N/D
11262	AX-212294353	AP_var_3006	NW_015441279	227329	T // C	1,61E-12	1,10E-10	outlier	0,029451	0,32782	n.s.	#N/D
1418	AX-212314942	Apop_07441	NW_015441063	1016603	A // T	2,71E-05	0,000353521	outlier	0,039295	0,21044	n.s.	#N/D
3768	AX-198060374	Apop_00421	NW_015441093	860033	A // G	4,13E-05	0,000518631	outlier	0,027652	0,36627	n.s.	#N/D
19076	AX-212317797	Apop_10280	NW_015442053	82417	A // G	6,67E-11	3,07E-09	outlier	0,095466	0,00090862	outlier	#N/D
7754	AX-212315924	Apop_08406	NW_015441169	449894	T // C	1,50E-07	3,25E-06	outlier	0,032639	0,28154	n.s.	#N/D
1446	AX-212314342	Apop_06858	NW_015441063	1262159	T // C	0,000203379	0,00194642	outlier	0,073935	0,084554	n.s.	#N/D
1444	AX-198059932	Apop_00148	NW_015441063	1260313	A // G	0,000203379	0,00194642	outlier	0,078042	0,066411	n.s.	#N/D
1442	AX-197962499	Apop_00147	NW_015441063	1259966	A // G	0,000203379	0,00194642	outlier	0,076843	0,075865	n.s.	#N/D
1438	AX-212299176	Apop_04977	NW_015441063	1257490	T // G	0,000203379	0,00194642	outlier	0,076058	0,072321	n.s.	#N/D
10212	AX-212302943	Apop_08713	NW_015441240	162498	T // C	3,27E-05	0,000417017	outlier	0,037796	0,24519	n.s.	#N/D
4262	AX-212292433	AP_var_1083	NW_015441103	1090334	A // G	2,72E-05	0,000353521	outlier	0,029833	0,34441	n.s.	#N/D
7728	AX-212314770	Apop_07290	NW_015441169	148784	T // C	0,001010342	0,007242988	outlier	0,030481	0,35001	n.s.	#N/D
14366	AX-212303635	Apop_09405	NW_015441415	87222	A // C	0,000248393	0,002291299	outlier	0,031336	0,29618	n.s.	#N/D
7292	AX-198045027	Afix_06620	NW_015441161	210899	T // C	1,95E-06	3,27E-05	outlier	0,060025	0,10233	n.s.	#N/D
13062	AX-212308225	AP_var_3592	NW_015441362	121531	T // C	1,35E-05	0,000190733	outlier	0,036789	0,24135	n.s.	#N/D
12960	AX-197964977	Apop_01598	NW_015441355	408277	A // G	0,000323199	0,00283775	outlier	0,026297	0,44717	n.s.	#N/D
3988	AX-212301998	Apop_07785	NW_015441096	708887	T // C	0,001131744	0,007993548	outlier	0,03745	0,255	n.s.	#N/D
3396	AX-198060324	Apop_00388	NW_015441090	187231	A // G	1,29E-05	0,000183924	outlier	0,025064	0,46138	n.s.	#N/D
10038	AX-212299473	Apop_05274	NW_015441230	517848	T // C	2,60E-05	0,000341459	outlier	0,029722	0,32322	n.s.	#N/D
6736	AX-212315714	Apop_08196	NW_015441148	741367	T // C	0,000263153	0,002404283	outlier	0,036839	0,25574	n.s.	#N/D
6744	AX-212315715	Apop_08197	NW_015441148	765282	A // G	0,000508755	0,004135029	outlier	0,036219	0,26951	n.s.	#N/D
17926	AX-197966016	Apop_02223	NW_015441749	142594	A // G	6,87E-07	1,30E-05	outlier	0,037312	0,22811	n.s.	#N/D
7730	AX-212299406	Apop_05207	NW_015441169	149794	T // C	0,000542318	0,004333176	outlier	0,029551	0,3543	n.s.	#N/D
15880	AX-212295703	AP_var_4359	NW_015441505	296489	A // G	1,19E-06	2,12E-05	outlier	0,027243	0,38119	n.s.	#N/D
7738	AX-197963719	Apop_00869	NW_015441169	217565	T // C	0,000438091	0,003664655	outlier	0,028597	0,3692	n.s.	#N/D
12502	AX-197964841	Apop_01509	NW_015441330	442437	C // G	0,000208658	0,001982081	outlier	0,046371	0,15753	n.s.	#N/D
8082	AX-212293400	AP_var_2053	NW_015441178	611595	T // C	5,54E-05	0,000680156	outlier	0,031653	0,30302	n.s.	#N/D
7660	AX-212297844	Apop_03644	NW_015441168	331735	A // G	3,05E-06	4,90E-05	outlier	0,028503	0,36803	n.s.	#N/D
7358	AX-198061022	Apop_00825	NW_015441162	50958	A // T	2,83E-05	0,000367005	outlier	0,024413	0,48021	n.s.	#N/D
1480	AX-212311697	Apop_04211	NW_015441063	1613083	C // G	2,48E-05	0,00032916	outlier	0,02749	0,40336	n.s.	#N/D
15362	AX-212303751	Apop_09521	NW_015441475	55725	A // C	1,45E-06	2,54E-05	outlier	0,028569	0,38785	n.s.	#N/D
9092	AX-212316057	Apop_08539	NW_015441202	148993	A // G	0,000634725	0,004918688	outlier	0,032029	0,32388	n.s.	#N/D
5530	AX-212315514	Apop_08013	NW_015441122	686677	T // G	1,87E-06	3,14E-05	outlier	0,025311	0,49503	n.s.	#N/D
14620	AX-198060083	Apop_00244	NW_015441435	126746	A // G	1,81E-07	3,86E-06	outlier	0,02843	0,39167	n.s.	#N/D

10112	AX-197964249	Apop_01178	NW_015441236	465439	A // T	1,44E-05	0,000201626	outlier	0,024814	0,45417	n.s.	#N/D
4252	AX-198060485	Apop_00495	NW_015441103	773056	T // C	1,30E-05	0,0001846	outlier	0,025459	0,43538	n.s.	#N/D
16664	AX-212312301	Apop_04816	NW_015441579	128737	A // G	3,55E-08	9,31E-07	outlier	0,030827	0,31184	n.s.	#N/D
10192	AX-212316225	Apop_08707	NW_015441238	364639	C // G	1,92E-05	0,000261104	outlier	0,04282	0,17974	n.s.	#N/D
19222	AX-212317834	Apop_10317	NW_015442137	33781	A // G	0,001325851	0,00908587	outlier	0,023987	0,5226	n.s.	#N/D
5528	AX-212315513	Apop_08012	NW_015441122	662686	A // G	8,84E-08	2,05E-06	outlier	0,026916	0,44043	n.s.	#N/D
15248	AX-212308781	AP_var_4148	NW_015441464	248273	A // C	0,00096148	0,006957855	outlier	0,024493	0,48402	n.s.	#N/D
10646	AX-212316308	Apop_08790	NW_015441259	507375	T // C	4,68E-05	0,000583173	outlier	0,024273	0,50518	n.s.	#N/D
17684	AX-212317511	Apop_09993	NW_015441708	64616	T // G	0,000543322	0,004333176	outlier	0,025632	0,44717	n.s.	#N/D
10316	AX-198061651	Apop_01213	NW_015441248	350972	A // G	8,81E-05	0,00100939	outlier	0,061398	0,11002	n.s.	#N/D
7542	AX-212302596	Apop_08366	NW_015441164	644970	T // C	0,000502738	0,004094814	outlier	0,023185	0,55953	n.s.	#N/D
13300	AX-212316742	Apop_09224	NW_015441365	409321	T // C	0,00011897	0,001308729	outlier	0,025451	0,46918	n.s.	#N/D
5604	AX-212302240	Apop_08027	NW_015441124	615895	T // C	0,00117863	0,008278884	outlier	0,024339	0,52038	n.s.	#N/D
15354	AX-197965442	Apop_01882	NW_015441472	357634	A // G	0,000619895	0,004833118	outlier	0,023909	0,52386	n.s.	#N/D
11790	AX-198079021	Apop_02858	NW_015441302	475986	T // C	0,000226216	0,002129799	outlier	0,024049	0,51025	n.s.	#N/D
9446	AX-212316112	Apop_08594	NW_015441213	100901	A // T	7,16E-05	0,000843682	outlier	0,023676	0,54419	n.s.	#N/D
11800	AX-197940163	Afix_01585	NW_015441302	496006	T // C	6,86E-05	0,000815414	outlier	0,022839	0,56421	n.s.	#N/D
11080	AX-212303094	Apop_08864	NW_015441273	223964	A // G	0,000159014	0,001633826	outlier	0,023762	0,52418	n.s.	#N/D
11076	AX-212303092	Apop_08862	NW_015441273	207535	T // G	0,000159014	0,001633826	outlier	0,023567	0,52667	n.s.	#N/D
16094	AX-212317131	Apop_09613	NW_015441521	35706	A // G	0,00057909	0,004570833	outlier	0,023237	0,55768	n.s.	#N/D
6142	AX-212306153	AP_var_1517	NW_015441137	90831	T // C	0,000329548	0,002880282	outlier	0,023072	0,555	n.s.	#N/D
2850	AX-198060202	Apop_00311	NW_015441079	481914	T // C	0,00063683	0,004921305	outlier	0,023303	0,55581	n.s.	#N/D
700	AX-212301520	Apop_07332	NW_015441058	672420	A // G	0,000233247	0,002177826	outlier	0,022423	0,6243	n.s.	#N/D
6754	AX-212315716	Apop_08198	NW_015441148	812475	A // G	0,000348538	0,003005096	outlier	0,024686	0,49753	n.s.	#N/D
7546	AX-212315885	Apop_08367	NW_015441164	650081	A // G	0,001404266	0,009449458	outlier	0,023759	0,52386	n.s.	#N/D
9110	AX-197963957	Apop_01009	NW_015441202	566188	T // C	0,000667047	0,00511737	outlier	0,021503	0,78575	n.s.	#N/D
16096	AX-198062852	Apop_01963	NW_015441521	136440	A // G	0,00022647	0,002129799	outlier	0,023663	0,51192	n.s.	#N/D
19518	AX-212297266	AP_var_5922	NW_015442468	20698	C // G	0,000388184	0,00330965	outlier	0,044351	0,18896	n.s.	#N/D
12454	AX-198062096	Apop_01488	NW_015441326	316088	A // G	2,36E-05	0,000315341	outlier	0,049779	0,15753	n.s.	#N/D
12460	AX-198062104	Apop_01493	NW_015441326	349283	A // G	2,36E-05	0,000315341	outlier	0,046563	0,17394	n.s.	#N/D
12462	AX-197964817	Apop_01495	NW_015441326	351471	C // G	2,36E-05	0,000315341	outlier	0,049469	0,15834	n.s.	#N/D
15998	AX-212295749	AP_var_4405	NW_015441515	188931	C // G	6,03E-05	0,000723159	outlier	0,02228	0,58759	n.s.	#N/D
19384	AX-198063480	Apop_02370	NW_015442268	48376	T // G	0,00136777	0,009300265	outlier	0,023363	0,57664	n.s.	#N/D
16152	AX-212303854	Apop_09624	NW_015441525	140659	A // T	7,20E-08	1,71E-06	outlier	0,028521	0,35733	n.s.	#N/D
11466	AX-212294403	AP_var_3056	NW_015441287	205248	A // C	0,000541084	0,004333176	outlier	0,021866	0,68367	n.s.	#N/D
1252	AX-198059842	Apop_00095	NW_015441061	1817312	A // G	0,000189122	0,001865957	outlier	0,022056	0,67702	n.s.	#N/D

16832	AX-212298251	Apop_04051	NW_015441597	30195	C // G	0,001481002	0,009741446	outlier	0,02186	0,67434	n.s.	#N/D
12340	AX-212316568	Apop_09050	NW_015441321	53046	T // C	0,00114049	0,008040489	outlier	0,02149	0,79054	n.s.	#N/D
18602	AX-212297773	Apop_03573	NW_015441889	117399	T // G	0,000767266	0,005816276	outlier	0,022008	0,62768	n.s.	#N/D
11788	AX-198079016	Apop_02855	NW_015441302	474908	A // C	0,000763022	0,005807108	outlier	0,021639	0,71163	n.s.	#N/D
2084	AX-197962635	Apop_00224	NW_015441071	592942	T // C	0,000332636	0,002886725	outlier	0,022172	0,60859	n.s.	#N/D
12456	AX-212307998	AP_var_3365	NW_015441326	341028	A // C	6,42E-05	0,000765099	outlier	0,04448	0,19063	n.s.	#N/D
14338	AX-212314659	Apop_07176	NW_015441413	174173	T // C	0,000124673	0,001352037	outlier	0,042801	0,21694	n.s.	#N/D
18452	AX-212296707	AP_var_5363	NW_015441858	147527	T // C	0,00091168	0,006725486	outlier	0,021448	0,79423	n.s.	#N/D
17282	AX-198063127	Apop_02142	NW_015441651	170377	T // C	0,000161066	0,001633826	outlier	0,021815	0,64237	n.s.	#N/D
9420	AX-197964055	Apop_01062	NW_015441211	559456	A // G	0,000100684	0,001136973	outlier	0,021577	0,62029	n.s.	#N/D
9444	AX-212316111	Apop_08593	NW_015441213	64504	C // G	0,001515909	0,009936899	outlier	0,021777	0,64094	n.s.	#N/D
3734	AX-212315242	Apop_07741	NW_015441093	272876	T // C	0,000354717	0,003051497	outlier	0,022199	0,65591	n.s.	#N/D
19080	AX-197966248	Apop_02348	NW_015442057	53484	A // G	9,28E-05	0,001057408	outlier	0,021952	0,60859	n.s.	#N/D
16510	AX-198062950	Apop_02028	NW_015441561	197364	A // G	0,000101143	0,0011388	outlier	0,021743	0,74183	n.s.	#N/D
5654	AX-198060699	Apop_00624	NW_015441126	497261	A // G	6,26E-06	9,35E-05	outlier	0,021393	0,76534	n.s.	#N/D
6122	AX-212315626	Apop_08108	NW_015441136	786540	A // G	0,000877216	0,00652393	outlier	0,021573	0,66461	n.s.	#N/D
17522	AX-212313826	Apop_06329	NW_015441682	172380	A // C	0,000468047	0,003844984	outlier	0,021315	0,8628	n.s.	#N/D
10034	AX-212311850	Apop_04364	NW_015441230	182207	C // G	0,000468047	0,003844984	outlier	0,021524	0,838	n.s.	#N/D
7304	AX-197948098	Afix_06635	NW_015441161	330405	C // G	0,000420928	0,003541499	outlier	0,021935	0,66957	n.s.	#N/D
1544	AX-212304921	AP_var_0276	NW_015441065	818376	C // G	1,21E-07	2,69E-06	outlier	0,021264	0,77843	n.s.	#N/D
16728	AX-212317283	Apop_09765	NW_015441588	197397	T // C	0,001207727	0,008416103	outlier	0,022027	0,66711	n.s.	#N/D
2500	AX-212301787	Apop_07574	NW_015441076	99148	C // G	0,001016111	0,007270732	outlier	0,021194	0,76062	n.s.	#N/D
2516	AX-197962697	Apop_00260	NW_015441076	407362	C // G	0,001457675	0,009671096	outlier	0,023308	0,59227	n.s.	#N/D
4794	AX-198060548	Apop_00535	NW_015441108	700479	T // C	0,000627877	0,004875491	outlier	0,02078	0,85307	n.s.	#N/D
19368	AX-198054606	Afix_12434	NW_015442238	65687	T // C	0,000493817	0,004030729	outlier	0,021371	0,82425	n.s.	#N/D
17422	AX-198063142	Apop_02151	NW_015441659	87575	A // G	0,001392268	0,009416662	outlier	0,020924	0,86133	n.s.	#N/D
17752	AX-212304237	Apop_10007	NW_015441712	210033	T // C	0,000869624	0,006495794	outlier	0,020879	0,88091	n.s.	#N/D
3158	AX-197962828	Apop_00343	NW_015441083	712286	T // C	0,001340833	0,009149608	outlier	0,028316	0,38287	n.s.	#N/D
13108	AX-197954984	Afix_10916	NW_015441363	64072	T // C	0,000703909	0,005389358	outlier	0,021066	0,8628	n.s.	#N/D
16666	AX-212303972	Apop_09742	NW_015441579	137781	C // G	0,00026906	0,002446566	outlier	0,020468	0,88963	n.s.	#N/D
11096	AX-197952870	Afix_09582	NW_015441274	3585	C // G	0,001213366	0,008430068	outlier	0,020746	0,86419	n.s.	#N/D

Protein accessic	mRNA accession	Adig genome v2	GO Terms	KEGG	SNPeff prediction	Putative_imp	DNA change	AA change	cDNA_position / cDNA_len	CDS_position / CDS_len	Protein_position / Protein_len	Distance to feature:
XP_015748225.	XM_015892739	aug_v2a.00311	GO:0005524; G	#N/D	3_prime_UTR_variant	MODIFIER	c.*496T>C					496
XP_015750347.	XM_015894861	aug_v2a.07121	#N/D	#N/D	3_prime_UTR_variant	MODIFIER	c.*56A>G					56
XP_015751934.	XM_015896448	#N/D	#N/D	#N/D	3_prime_UTR_variant	MODIFIER	c.*207G>C					207
XP_015753011.	XM_015897525	aug_v2a.16824	#N/D	#N/D	3_prime_UTR_variant	MODIFIER	c.*2181T>C					2181
XP_015753573.	XM_015898087	aug_v2a.09315	GO:0005829; G	K10886	3_prime_UTR_variant	MODIFIER	c.*1733A>G					1733
XP_015753644.	XM_015898158	aug_v2a.04792	GO:0016021	K19370	3_prime_UTR_variant	MODIFIER	c.*866C>G					866
XP_015754996.	XM_015899510	aug_v2a.17340	GO:0005524; G	#N/D	3_prime_UTR_variant	MODIFIER	c.*102A>G					102
XP_015759041.	XM_015903555	aug_v2a.12203	GO:0030424; G	K05122	3_prime_UTR_variant	MODIFIER	c.*1457A>C					1457
XP_015765243.	XM_015909757	aug_v2a.15540	#N/D	#N/D	3_prime_UTR_variant	MODIFIER	c.*34T>C					34
XP_015766108.	XM_015910622	aug_v2a.06707	GO:0005912; G	K04237	3_prime_UTR_variant	MODIFIER	c.*109A>G					109
XP_015763353.	XM_015907867	aug_v2a.21182	GO:0015629; G	K05700	3_prime_UTR_variant	MODIFIER	c.*257T>C					257
XP_015766287.	XM_015910801	aug_v2a.01851	GO:0000932; G	#N/D	3_prime_UTR_variant	MODIFIER	c.*666T>C					666
XP_015770102.	XM_015914616	aug_v2a.06444	GO:0005737; G	K08465	3_prime_UTR_variant	MODIFIER	c.*711A>G					711
XP_015771951.	XM_015916465	aug_v2a.11999	GO:0030054; G	#N/D	3_prime_UTR_variant	MODIFIER	c.*16T>C					16
XP_015773275.	XM_015917789	aug_v2a.17397	GO:0005776; G	K00920	3_prime_UTR_variant	MODIFIER	c.*271A>G					271
XP_015773668.	XM_015918182	#N/D	#N/D	#N/D	3_prime_UTR_variant	MODIFIER	c.*160A>G					160
XP_015773743.	XM_015918257	aug_v2a.09408	GO:0000139; G	K00738	3_prime_UTR_variant	MODIFIER	c.*857T>G					857
XP_015779272.	XM_015923786	aug_v2a.08615	#N/D	#N/D	3_prime_UTR_variant	MODIFIER	c.*48T>G					48
XP_015748788.	XM_015893302	aug_v2a.15582	GO:0000139; G	K20293	intron_variant	MODIFIER	c.629-84T>C					
XP_015775865.	XM_015920379	aug_v2a.09745	GO:0000166 G	K08844	synonymous_variant	LOW	c.5732T>G	p.Ser1911Ser	6052/7464	5732/6687	1911/2228	
XP_015761708.	XM_015906222	aug_v2a.01491	GO:0005737; G	K20402	3_prime_UTR_variant	MODIFIER	c.*45T>G					45
XP_015754999.	XM_015899513	aug_v2a.17344	GO:0005829; G	#N/D	3_prime_UTR_variant	MODIFIER	c.*364G>C					364
XP_015757455.	XM_015901969	aug_v2a.16725	GO:0000166 G	K06942	missense_variant	MODERATE	c.1432T>C	p.Phe478Leu	1550/2112	1432/1728	478/575	
XP_015762529.	XM_015907043	aug_v2a.17709	GO:0016020; G	#N/D	3_prime_UTR_variant	MODIFIER	c.*168G>C					168
XP_015756351.	XM_015900865	aug_v2a.03461	GO:0001701 G	K21436	synonymous_variant	LOW	c.65T>C	p.Pro22Pro	251/2874	65/2688	22/895	
XP_015768476.	XM_015912990	aug_v2a.05254	GO:0016021	#N/D	3_prime_UTR_variant	MODIFIER	c.*213T>C					213
XP_015758161.	XM_015902675	aug_v2a.06441	GO:0030424; G	K05126	3_prime_UTR_variant	MODIFIER	c.*315A>C					315

XP_015770883.:	XM_015915397	aug_v2a.07682	GO:0003677	K20478	synonymous_variant	LOW	c.7250T>G	p.Arg2417Arg	7260/11892	7250/11754	2417/3917	
XP_015772251.:	XM_015916765	aug_v2a.14913	GO:0003677 GC	K05638	synonymous_variant	LOW	c.560T>A	p.Tyr187Tyr	743/2574	560/1698	187/565	
XP_015761721.:	XM_015906235	aug_v2a.01487	GO:0034451; G(	K21765	3_prime_UTR_variant	MODIFIER	c.*442G>C					442
XP_015749294.:	XM_015893808	aug_v2a.15185	#N/D	K11663	3_prime_UTR_variant	MODIFIER	c.*223C>G					223
XP_015751069.:	XM_015895583	aug_v2a.13882	GO:0008017; G(	#N/D	5_prime_UTR_prematu	LOW	c.-32A>G					
XP_015760868.:	XM_015905382	aug_v2a.10971	GO:0072562; G(	K10104	5_prime_UTR_prematu	LOW	c.-237T>C					
XP_015766225.:	XM_015910739	aug_v2a.01852	GO:0000932; G(	K12035	5_prime_UTR_prematu	LOW	c.-432A>G					
XP_015770891.:	XM_015915405	aug_v2a.14368	#N/D	#N/D	5_prime_UTR_prematu	LOW	c.-6A>G					
XP_015761706.:	XM_015906220	aug_v2a.01490	GO:0003841 GC	K19007	synonymous_variant	LOW	c.344C>G	p.Arg115Arg	417/1082	344/951	115/316	
XP_015755137.:	XM_015899651	aug_v2a.23924	#N/D	#N/D	5_prime_UTR_variant	MODIFIER	c.-29T>C					29
XP_015765243.:	XM_015909757	aug_v2a.15540	#N/D	#N/D	3_prime_UTR_variant	MODIFIER	c.*229A>G					229
XP_015765243.:	XM_015909757	aug_v2a.15540	#N/D	#N/D	3_prime_UTR_variant	MODIFIER	c.*615A>G					615
XP_015763473.:	XM_015907987	aug_v2a.11997	GO:0005509	#N/D	5_prime_UTR_variant	MODIFIER	c.-16T>C					16
XP_015766100.:	XM_015910614	aug_v2a.09265	#N/D	#N/D	5_prime_UTR_variant	MODIFIER	c.-89T>C					89
XP_015766857.:	XM_015911371	aug_v2a.11511	GO:0016021	#N/D	5_prime_UTR_variant	MODIFIER	c.-40A>T					40
XP_015768854.:	XM_015913368	aug_v2a.07763	GO:0005634; G(	K14589	5_prime_UTR_variant	MODIFIER	c.-52A>C					52
XP_015769258.:	XM_015913772	aug_v2a.20502	GO:0005576; G(	K23318	5_prime_UTR_variant	MODIFIER	c.-289A>G					1372
XP_015769652.:	XM_015914166	aug_v2a.10630	GO:0005622; G(	#N/D	5_prime_UTR_variant	MODIFIER	c.-350T>C					350
XP_015776763.:	XM_015921277	aug_v2a.07275	GO:0005737; G(	K03440	5_prime_UTR_variant	MODIFIER	c.-469A>G					469
XP_015778673.:	XM_015923187	aug_v2a.17233	GO:0005634; G(	#N/D	5_prime_UTR_variant	MODIFIER	c.-65A>G					65
XP_015780625.:	XM_015925139	aug_v2a.08859	GO:0004497 GC	K00488	synonymous_variant	LOW	c.1121A>G	p.Arg374Arg	1287/2076	1121/1596	374/531	
XP_015754395.:	XM_015898909	aug_v2a.01024	GO:0032592; G(	K05657	5_prime_UTR_prematu	LOW	c.-420A>G					
XP_015765242.:	XM_015909756	#N/D	#N/D	#N/D	5_prime_UTR_prematu	LOW	c.-56A>G					
XP_015763165.:	XM_015907679	aug_v2a.06182	GO:0004842 GC	K10595	synonymous_variant	LOW	c.3480T>C	p.Pro1160Pro	3615/11573	3480/11187	1160/3728	
XP_015763165.:	XM_015907679	aug_v2a.06182	GO:0004842 GC	K10595	synonymous_variant	LOW	c.6024A>G	p.Pro2008Pro	6159/11573	6024/11187	2008/3728	
XP_015765661.:	XM_015910175	aug_v2a.17470	GO:0004857	#N/D	splice_region_variant&:	LOW	c.654A>G	p.Ala218Ala	814/1855	654/735	218/244	
XP_015760790.:	XM_015905304	aug_v2a.24587	GO:0005737; G(	K09366	5_prime_UTR_variant	MODIFIER	c.-647T>C					647
#N/D	#N/D	#N/D	#N/D	#N/D	downstream_gene_vari	MODIFIER	c.*1547A>G					878
#N/D	#N/D	#N/D	#N/D	#N/D	downstream_gene_vari	MODIFIER	c.*6451T>C					2197

#N/D	#N/D	#N/D	#N/D	#N/D	downstream_gene_vari	MODIFIER	c.*4820C>G					4406
XP_015773513.	XM_015918027	aug_v2a.00401	GO:0004871 GC	K04594	synonymous_variant	LOW	c.1086T>C	p.Leu362Leu	1157/2801	1086/1866	362/621	
XP_015771406.	XM_015915920	#N/D	#N/D	#N/D	5_prime_UTR_variant	MODIFIER	c.-209T>C					209
#N/D	#N/D	#N/D	#N/D	#N/D	downstream_gene_vari	MODIFIER	c.*7180A>G					1995
#N/D	#N/D	#N/D	#N/D	#N/D	downstream_gene_vari	MODIFIER	c.*4875C>G					4501
#N/D	#N/D	#N/D	#N/D	#N/D	downstream_gene_vari	MODIFIER	n.*2934A>G					2934
#N/D	#N/D	#N/D	#N/D	#N/D	downstream_gene_vari	MODIFIER	c.*4476A>T					3295
#N/D	#N/D	#N/D	#N/D	#N/D	downstream_gene_vari	MODIFIER	c.*3220T>C					2285
XP_015761821.	XM_015906335	aug_v2a.14028	GO:0005085 GC	K06277	synonymous_variant	LOW	c.1260A>C	p.Gly420Gly	2843/5193	1260/2781	420/926	
#N/D	#N/D	#N/D	#N/D	#N/D	downstream_gene_vari	MODIFIER	c.*3328A>G					343
#N/D	#N/D	#N/D	#N/D	#N/D	downstream_gene_vari	MODIFIER	c.*26589A>T					4249
#N/D	#N/D	#N/D	#N/D	#N/D	downstream_gene_vari	MODIFIER	c.*11593T>C					4681
#N/D	#N/D	#N/D	#N/D	#N/D	downstream_gene_vari	MODIFIER	c.*6788T>C					621
#N/D	#N/D	#N/D	#N/D	#N/D	downstream_gene_vari	MODIFIER	c.*8343A>G					2498
#N/D	#N/D	#N/D	#N/D	#N/D	downstream_gene_vari	MODIFIER	c.*9634A>G					3789
#N/D	#N/D	#N/D	#N/D	#N/D	downstream_gene_vari	MODIFIER	c.*2208A>G					555
#N/D	#N/D	#N/D	#N/D	#N/D	downstream_gene_vari	MODIFIER	c.*14650A>T					1007
#N/D	#N/D	#N/D	#N/D	#N/D	downstream_gene_vari	MODIFIER	n.*4731T>G					4731
#N/D	#N/D	#N/D	#N/D	#N/D	downstream_gene_vari	MODIFIER	n.*4682T>C					4682
#N/D	#N/D	#N/D	#N/D	#N/D	downstream_gene_vari	MODIFIER	c.*20664C>G					3431
#N/D	#N/D	#N/D	#N/D	#N/D	downstream_gene_vari	MODIFIER	c.*3213T>C					516
#N/D	#N/D	#N/D	#N/D	#N/D	downstream_gene_vari	MODIFIER	c.*2447A>G					2172
#N/D	#N/D	#N/D	#N/D	#N/D	downstream_gene_vari	MODIFIER	c.*8598C>G					4342
#N/D	#N/D	#N/D	#N/D	#N/D	downstream_gene_vari	MODIFIER	n.*527T>G					527
#N/D	#N/D	#N/D	#N/D	#N/D	downstream_gene_vari	MODIFIER	n.*2328T>C					2328
#N/D	#N/D	#N/D	#N/D	#N/D	downstream_gene_vari	MODIFIER	c.*6041A>G					1515
#N/D	#N/D	#N/D	#N/D	#N/D	downstream_gene_vari	MODIFIER	c.*4368C>G					4168
#N/D	#N/D	#N/D	#N/D	#N/D	downstream_gene_vari	MODIFIER	c.*42204A>G					4211
#N/D	#N/D	#N/D	#N/D	#N/D	downstream_gene_vari	MODIFIER	n.*329A>G					329
#N/D	#N/D	#N/D	#N/D	#N/D	downstream_gene_vari	MODIFIER	n.*4338T>C					4338
#N/D	#N/D	#N/D	#N/D	#N/D	downstream_gene_vari	MODIFIER	n.*1050C>G					1050
#N/D	#N/D	#N/D	#N/D	#N/D	downstream_gene_vari	MODIFIER	c.*11023A>T					1053
#N/D	#N/D	#N/D	#N/D	#N/D	downstream_gene_vari	MODIFIER	c.*13073T>C					1196
XP_015754071.	XM_015898585	aug_v2a.13969	GO:0005198 GC	K04659	synonymous_variant	LOW	c.594A>G	p.Ala198Ala	594/3274	594/3075	198/1024	

XP_015771269.	XM_015915783	aug_v2a.19221	#N/D	#N/D	5_prime_UTR_variant	MODIFIER	c.-1426G>C					1426
#N/D	#N/D	#N/D	#N/D	#N/D	downstream_gene_vari	MODIFIER	n.*274A>G					274
#N/D	#N/D	#N/D	#N/D	#N/D	downstream_gene_vari	MODIFIER	c.*3217T>G					2773
XP_015754993.	XM_015899507	aug_v2a.10966	GO:0005215 GC	K14638	missense_variant	MODERATE	c.436A>G	p.Asn146Asp	1306/2811	436/1554	146/517	
#N/D	#N/D	#N/D	#N/D	#N/D	downstream_gene_vari	MODIFIER	c.*8444T>C					4084
#N/D	#N/D	#N/D	#N/D	#N/D	downstream_gene_vari	MODIFIER	c.*4588T>G					4357
#N/D	#N/D	#N/D	#N/D	#N/D	downstream_gene_vari	MODIFIER	c.*2729T>C					2413
XP_015760757.	XM_015905271	aug_v2a.24586	GO:0005488 GC	#N/D	synonymous_variant	LOW	c.1022T>C	p.Ala341Ala	1356/10573	1022/9609	341/3202	
#N/D	#N/D	#N/D	#N/D	#N/D	downstream_gene_vari	MODIFIER	c.*37786A>C					1419
XP_015755542.	XM_015900056	aug_v2a.10189	GO:0005509 GC	K17341	synonymous_variant	LOW	c.1032A>C	p.Ile344Ile	1049/2002	1032/1767	344/588	
#N/D	#N/D	#N/D	#N/D	#N/D	downstream_gene_vari	MODIFIER	c.*14168A>T					3797
#N/D	#N/D	#N/D	#N/D	#N/D	downstream_gene_vari	MODIFIER	n.*3808T>C					3808
#N/D	#N/D	#N/D	#N/D	#N/D	downstream_gene_vari	MODIFIER	c.*27201A>G					4861
#N/D	#N/D	#N/D	#N/D	#N/D	downstream_gene_vari	MODIFIER	n.*1108T>G					1108
#N/D	#N/D	#N/D	#N/D	#N/D	intergenic_region	MODIFIER						
#N/D	#N/D	#N/D	#N/D	#N/D	intergenic_region	MODIFIER	n.134642C>G					
#N/D	#N/D	#N/D	#N/D	#N/D	intergenic_region	MODIFIER	n.43692A>C					
#N/D	#N/D	#N/D	#N/D	#N/D	intergenic_region	MODIFIER	n.92786A>G					
#N/D	#N/D	#N/D	#N/D	#N/D	intergenic_region	MODIFIER	n.702995A>G					
#N/D	#N/D	#N/D	#N/D	#N/D	intergenic_region	MODIFIER	n.684750A>C					
#N/D	#N/D	#N/D	#N/D	#N/D	intergenic_region	MODIFIER	n.33162T>C					
#N/D	#N/D	#N/D	#N/D	#N/D	intergenic_region	MODIFIER	n.3564T>C					
#N/D	#N/D	#N/D	#N/D	#N/D	intergenic_region	MODIFIER	n.447531A>G					
#N/D	#N/D	#N/D	#N/D	#N/D	intergenic_region	MODIFIER	n.185399T>C					
#N/D	#N/D	#N/D	#N/D	#N/D	intergenic_region	MODIFIER	n.130092A>C					
#N/D	#N/D	#N/D	#N/D	#N/D	intergenic_region	MODIFIER	n.512200C>G					
#N/D	#N/D	#N/D	#N/D	#N/D	intergenic_region	MODIFIER	n.153101A>G					
#N/D	#N/D	#N/D	#N/D	#N/D	intergenic_region	MODIFIER	n.466817T>C					
#N/D	#N/D	#N/D	#N/D	#N/D	intergenic_region	MODIFIER	n.4328A>G					
#N/D	#N/D	#N/D	#N/D	#N/D	intergenic_region	MODIFIER	n.2074347A>C					
#N/D	#N/D	#N/D	#N/D	#N/D	intergenic_region	MODIFIER	n.2072842T>G					
#N/D	#N/D	#N/D	#N/D	#N/D	intergenic_region	MODIFIER	n.289067C>G					
#N/D	#N/D	#N/D	#N/D	#N/D	intergenic_region	MODIFIER	n.2069866A>G					
#N/D	#N/D	#N/D	#N/D	#N/D	intergenic_region	MODIFIER	n.375272A>C					

#N/D	#N/D	#N/D	#N/D	#N/D	intergenic_region	MODIFIER	n.369986A>C					
#N/D	#N/D	#N/D	#N/D	#N/D	intergenic_region	MODIFIER	n.186700T>G					
#N/D	#N/D	#N/D	#N/D	#N/D	intergenic_region	MODIFIER	n.86395T>C					
#N/D	#N/D	#N/D	#N/D	#N/D	intergenic_region	MODIFIER	n.97513C>G					
#N/D	#N/D	#N/D	#N/D	#N/D	intergenic_region	MODIFIER	n.370272A>C					
#N/D	#N/D	#N/D	#N/D	#N/D	intergenic_region	MODIFIER	n.75620A>C					
#N/D	#N/D	#N/D	#N/D	#N/D	intergenic_region	MODIFIER	n.42830A>G					
XP_015767513.	XM_015912027	aug_v2a.09920	GO:0005524; G	K17916	synonymous_variant	LOW	c.3054T>C	p.Thr1018Thr	3054/3603	3054/3603	1018/1200	
#N/D	#N/D	#N/D	#N/D	#N/D	downstream_gene_vari	MODIFIER	n.*1357T>C					1357
#N/D	#N/D	#N/D	#N/D	#N/D	downstream_gene_vari	MODIFIER	n.*3299T>G					3299
#N/D	#N/D	#N/D	#N/D	#N/D	intergenic_region	MODIFIER	n.79755T>C					
#N/D	#N/D	#N/D	#N/D	#N/D	intergenic_region	MODIFIER	n.333318C>G					
#N/D	#N/D	#N/D	#N/D	#N/D	intergenic_region	MODIFIER	n.11900A>G					
#N/D	#N/D	#N/D	#N/D	#N/D	intergenic_region	MODIFIER						
XP_015757452.	XM_015901966	aug_v2a.16722	GO:0005634; G	K09372	intron_variant	MODIFIER	c.805+275T>C					
#N/D	#N/D	#N/D	#N/D	#N/D	intragenic_variant	MODIFIER	n.15998A>T					
#N/D	#N/D	#N/D	#N/D	#N/D	intergenic_region	MODIFIER	n.254569T>C					
XP_015773399.	XM_015917913	aug_v2a.12815	GO:0005634; G	#N/D	3_prime_UTR_variant	MODIFIER	c.*524A>G					524
XP_015766763.	XM_015911277	aug_v2a.15593	GO:0005730; G	K14856	splice_region_variant&i	LOW	c.1962+8A>G					
#N/D	#N/D	#N/D	#N/D	#N/D	intergenic_region	MODIFIER	n.389135A>T					
XP_015760790.	XM_015905304	aug_v2a.24587	GO:0005737; G	K09366	5_prime_UTR_variant	MODIFIER	c.-1388A>G					1388
#N/D	#N/D	#N/D	#N/D	#N/D	intergenic_region	MODIFIER	n.4960T>C					
XP_015778515.	XM_015923029	aug_v2a.20235	GO:0005737; G	#N/D	intron_variant	MODIFIER	c.207+230T>A					
XP_015754070.	XM_015898584	aug_v2a.14607	GO:0005737; G	K06838	intron_variant	MODIFIER	c.1560-2234T>G					
XP_015754070.	XM_015898584	aug_v2a.14607	GO:0005737; G	K06838	intron_variant	MODIFIER	c.885-680A>T					
#N/D	#N/D	#N/D	#N/D	#N/D	intergenic_region	MODIFIER	n.98378A>G					
#N/D	#N/D	#N/D	#N/D	#N/D	intragenic_variant	MODIFIER	n.1006913T>C					
XP_015754070.	XM_015898584	aug_v2a.14607	GO:0005737; G	K06838	splice_region_variant&i	LOW	c.1914-3T>C					
XP_015759566.	XM_015904080	aug_v2a.22531	GO:0005739; G	K00814	intron_variant	MODIFIER	c.445-37T>G					
XP_015759591.	XM_015904105	aug_v2a.09212	GO:0003676; G	#N/D	intron_variant	MODIFIER	c.631-132G>C					
XP_015772585.	XM_015917099	aug_v2a.04543	GO:0005576; G	K05129	intron_variant	MODIFIER	c.946+1615A>G					
XP_015758379.	XM_015902893	aug_v2a.12633	GO:0005576; G	#N/D	intron_variant	MODIFIER	c.-3-1286T>C					
XP_015760851.	XM_015905365	aug_v2a.05383	GO:0005622; G	#N/D	intron_variant	MODIFIER	c.1183-206A>G					
XP_015765248.	XM_015909762	aug_v2a.03315	GO:0005829; G	#N/D	intron_variant	MODIFIER	c.-45+649T>C					

XP_015747454.:	XM_015891968	aug_v2a.03778	GO:0005737; G(	K08129	intron_variant	MODIFIER	c.62+1412A>G					
XP_015748494.:	XM_015893008	aug_v2a.03123	GO:0005813; G(	#N/D	intron_variant	MODIFIER	c.547-72T>C					
XP_015748596.:	XM_015893110	aug_v2a.06535	GO:0005622; G(	K14416	intron_variant	MODIFIER	c.671+67A>G					
XP_015780736.:	XM_015925250	aug_v2a.10426	GO:0005886 GC	#N/D	synonymous_variant	LOW	c.2385T>C	p.Val795Val	2432/3390	2385/2856	795/951	
XP_015762588.:	XM_015907102	aug_v2a.07127	GO:0005622; G(	K08813	intron_variant	MODIFIER	c.300+13A>G					
XP_015749399.:	XM_015893913	#N/D	#N/D	#N/D	intron_variant	MODIFIER	c.-60+106A>G					
XP_015749946.:	XM_015894460	aug_v2a.13919	GO:0005634; G(	K09216	intron_variant	MODIFIER	c.100+160A>G					
XP_015750990.:	XM_015895504	aug_v2a.11167	GO:0042584; G(	K23924	intron_variant	MODIFIER	c.-4-144G>C					
XP_015751166.:	XM_015895680	aug_v2a.02852	GO:0005813; G(	K11584	intron_variant	MODIFIER	c.938+2915T>C					
XP_015751371.:	XM_015895885	aug_v2a.02085	GO:0043231; G(	K20091	intron_variant	MODIFIER	c.191+2163A>G					
XP_015751395.:	XM_015895909	aug_v2a.20705	GO:0005829; G(	K09048	intron_variant	MODIFIER	c.519+33T>G					
XP_015752405.:	XM_015896919	aug_v2a.15260	GO:0031410; G(	K04728	intron_variant	MODIFIER	c.8610+414T>C					
XP_015752627.:	XM_015897141	aug_v2a.02121	GO:0005737; G(	K12233	intron_variant	MODIFIER	c.1918-238T>C					
XP_015752778.:	XM_015897292	aug_v2a.12207	GO:0005737; G(	K13752	intron_variant	MODIFIER	c.442+190T>A					
XP_015752937.:	XM_015897451	aug_v2a.07078	GO:0005604; G(	K23383	intron_variant	MODIFIER	c.5445+315A>G					
XP_015753525.:	XM_015898039	aug_v2a.16181	GO:0016021	#N/D	intron_variant	MODIFIER	c.648+8050T>C					
XP_015754071.:	XM_015898585	aug_v2a.13969	GO:0031012; G(	K04659	intron_variant	MODIFIER	c.1357-633T>G					
XP_015775618.:	XM_015920132	aug_v2a.04700	GO:0005887; G(	#N/D	intron_variant	MODIFIER	c.507+31T>A					
XP_015754071.:	XM_015898585	aug_v2a.13969	GO:0031012; G(	K04659	intron_variant	MODIFIER	c.2032-179T>G					
XP_015754149.:	XM_015898663	aug_v2a.16129	GO:0019901; G(	K16831	intron_variant	MODIFIER	c.983-177A>G					
XP_015754445.:	XM_015898959	aug_v2a.01025	GO:0005615; G(	K21283	intron_variant	MODIFIER	c.-128-1352T>C					
XP_015755646.:	XM_015900160	aug_v2a.01326	GO:0007165	#N/D	intron_variant	MODIFIER	c.*34-246A>G					
XP_015755730.:	XM_015900244	aug_v2a.00533	GO:0016323; G(	K05665	intron_variant	MODIFIER	c.1437+59A>G					
XP_015756579.:	XM_015901093	aug_v2a.18002	GO:0005887; G(	K04905	intron_variant	MODIFIER	c.437-1562T>G					
XP_015757012.:	XM_015901526	aug_v2a.11065	GO:0017101; G(	K14163	intron_variant	MODIFIER	c.3468+85A>G					
XP_015760757.:	XM_015905271	aug_v2a.24586	GO:0005938; G(	#N/D	5_prime_UTR_variant	MODIFIER	c.-10T>C					10
XP_015757453.:	XM_015901967	aug_v2a.16723	GO:0005694; G(	K14787	intron_variant	MODIFIER	c.2391+82G>C					

XP_015757955.:	XM_015902469	aug_v2a.14055	GO:0016323; G(	#N/D	intron_variant	MODIFIER	c.2254-129T>G
XP_015780738.:	XM_015925252	aug_v2a.10427	GO:0005634; G(	#N/D	intron_variant	MODIFIER	c.494+131A>G
XP_015754070.:	XM_015898584	aug_v2a.14607	GO:0005737; G(	K06838	intron_variant	MODIFIER	c.1560-3316A>C
XP_015754070.:	XM_015898584	aug_v2a.14607	GO:0005737; G(	K06838	intron_variant	MODIFIER	c.2058-285A>G
XP_015758645.:	XM_015903159	aug_v2a.06770	GO:0005634; G(	K03124	intron_variant	MODIFIER	c.517+75A>C
XP_015776996.:	XM_015921510	aug_v2a.11702	GO:0005783; G(	K10661	intron_variant	MODIFIER	c.1302+48A>G
XP_015758694.:	XM_015903208	aug_v2a.24129	GO:0031410; G(	K10356	intron_variant	MODIFIER	c.2741+2705A>T
XP_015758816.:	XM_015903330	aug_v2a.16964	GO:0005578; G(	K08626	intron_variant	MODIFIER	c.1930-750A>G
XP_015758965.:	XM_015903479	aug_v2a.13150	GO:0008622; G(	K02324	intron_variant	MODIFIER	c.1680+326T>C
XP_015759041.:	XM_015903555	aug_v2a.12203	GO:0030424; G(	K05122	intron_variant	MODIFIER	c.281+2642T>C
XP_015759104.:	XM_015903618	aug_v2a.06747	GO:0005884; G(	#N/D	intron_variant	MODIFIER	c.1143+76T>C
XP_015759464.:	XM_015903978	aug_v2a.02339	GO:0005901; G(	K22125	intron_variant	MODIFIER	c.3877-224A>G
XP_015760444.:	XM_015904958	aug_v2a.04473	GO:0071889; G(	K20360	intron_variant	MODIFIER	c.1267+62A>G
XP_015769014.:	XM_015913528	aug_v2a.03037	GO:0015629; G(	K05700	intron_variant	MODIFIER	c.259-70T>G
XP_015760851.:	XM_015905365	aug_v2a.05383	GO:0005622; G(	#N/D	intron_variant	MODIFIER	c.710+66T>C
XP_015760957.:	XM_015905471	aug_v2a.17494	GO:0016021	K23879	intron_variant	MODIFIER	c.-4+535A>T
XP_015761455.:	XM_015905969	aug_v2a.08768	GO:0005737; G(	K14689	intron_variant	MODIFIER	c.612-422A>G
XP_015769014.:	XM_015913528	aug_v2a.03037	GO:0015629; G(	K05700	intron_variant	MODIFIER	c.3758+57C>G
XP_015763013.:	XM_015907527	aug_v2a.07326	GO:0005929; G(	#N/D	intron_variant	MODIFIER	c.1527+34T>C
XP_015763170.:	XM_015907684	aug_v2a.19674	GO:0005615; G(	K01374	intron_variant	MODIFIER	c.664-378T>C
XP_015747368.:	XM_015891882	aug_v2a.06843	GO:0005789; G(	K00710	intron_variant	MODIFIER	c.623-82T>C
XP_015763201.:	XM_015907715	aug_v2a.01744	GO:0005524; G(	K05305	intron_variant	MODIFIER	c.223-77A>G
XP_015764002.:	XM_015908516	aug_v2a.09304	GO:0016021; G(	K02842	intron_variant	MODIFIER	c.-388+633C>G
XP_015764161.:	XM_015908675	aug_v2a.20457	GO:0005509	K23851	intron_variant	MODIFIER	c.758+15G>C
XP_015764975.:	XM_015909489	aug_v2a.05494	GO:0016604; G(	K14947	intron_variant	MODIFIER	c.381+15C>G
XP_015765248.:	XM_015909762	aug_v2a.03315	GO:0005829; G(	#N/D	intron_variant	MODIFIER	c.-45+1909T>C
XP_015765248.:	XM_015909762	aug_v2a.03315	GO:0005829; G(	#N/D	intron_variant	MODIFIER	c.-45+1149T>C
XP_015777828.:	XM_015922342	aug_v2a.14263	GO:0005887; G(	K04984	intron_variant	MODIFIER	c.1419+270A>G
XP_015765333.:	XM_015909847	aug_v2a.16083	#N/D	#N/D	intron_variant	MODIFIER	c.779+231T>C

XP_015765352.	XM_015909866	aug_v2a.10335	#N/D	#N/D	intron_variant	MODIFIER	c.747+1377A>G					
XP_015768520.	XM_015913034	aug_v2a.11053	GO:0009986; G	K02583	intron_variant	MODIFIER	c.838+1375T>G					
XP_015766117.	XM_015910631	aug_v2a.20459	GO:0015629; G	K05700	intron_variant	MODIFIER	c.1864-31A>G					
XP_015763046.	XM_015907560	aug_v2a.13646	GO:0015629; G	K05700	intron_variant	MODIFIER	c.857-79C>G					
XP_015780485.	XM_015924999	aug_v2a.01298	GO:0015629; G	K05700	intron_variant	MODIFIER	c.353-427T>C					
XP_015771671.	XM_015916185	aug_v2a.21581	GO:0015629; G	K05700	intron_variant	MODIFIER	c.287+274A>G					
XP_015778941.	XM_015923455	aug_v2a.09473	GO:0015629; G	K05700	intron_variant	MODIFIER	c.2220+223A>T					
XP_015773281.	XM_015917795	aug_v2a.09602	GO:0015629; G	K05700	intron_variant	MODIFIER	c.1515+526T>C					
XP_015758988.	XM_015903502	aug_v2a.12922	GO:0015629; G	K05700	intron_variant	MODIFIER	c.2403+33A>G					
XP_015766285.	XM_015910799	aug_v2a.01853	GO:0015629; G	K05700	intron_variant	MODIFIER	c.1330+189C>G					
XP_015767289.	XM_015911803	aug_v2a.10178	GO:0015629; G	K05700	intron_variant	MODIFIER	c.329+2117A>G					
XP_015778671.	XM_015923185	aug_v2a.17055	GO:0015629; G	K05700	intron_variant	MODIFIER	c.860-231T>C					
XP_015766299.	XM_015910813	aug_v2a.23156	GO:0005604; G	#N/D	intron_variant	MODIFIER	c.5336-14430G>C					
XP_015768473.	XM_015912987	aug_v2a.05254	GO:0016021	#N/D	synonymous_variant	LOW	c.513A>G	p.Ser171Ser	830/1236	513/552	171/183	
XP_015775122.	XM_015919636	aug_v2a.07618	GO:0015629; G	K05700	intron_variant	MODIFIER	c.1995-314A>G					
XP_015767513.	XM_015912027	aug_v2a.09920	GO:0005524; G	K17916	intron_variant	MODIFIER	c.1376+304A>C					
XP_015767528.	XM_015912042	aug_v2a.09647	#N/D	#N/D	intron_variant	MODIFIER	c.1189-308G>C					
XP_015776239.	XM_015920753	aug_v2a.05694	GO:0016021; G	K05126	3_prime_UTR_variant	MODIFIER	c.*362A>G					362
XP_015768732.	XM_015913246	aug_v2a.10096	GO:0016021	#N/D	intron_variant	MODIFIER	c.549+365T>C					
XP_015765331.	XM_015909845	aug_v2a.06109	GO:0015629; G	K05700	intron_variant	MODIFIER	c.15+190A>G					
XP_015769652.	XM_015914166	aug_v2a.10630	GO:0005622; G	#N/D	intron_variant	MODIFIER	c.1699+134A>C					
XP_015748571.	XM_015893085	aug_v2a.23754	GO:0016021; G	K08465	intron_variant	MODIFIER	c.-309+294A>C					
XP_015770190.	XM_015914704	aug_v2a.06470	GO:0005509	K17307	intron_variant	MODIFIER	c.70-445G>C					
XP_015770715.	XM_015915229	aug_v2a.21189	GO:0016020; G	K11165	intron_variant	MODIFIER	c.241-317A>C					
XP_015771333.	XM_015915847	aug_v2a.06849	GO:0000145; G	K06110	intron_variant	MODIFIER	c.1247+15T>C					
XP_015772270.	XM_015916784	aug_v2a.14916	GO:0016021	#N/D	intron_variant	MODIFIER	c.747-24A>G					
XP_015772562.	XM_015917076	aug_v2a.02792	#N/D	#N/D	intron_variant	MODIFIER	c.36-27768A>G					
XP_015774674.	XM_015919188	aug_v2a.06219	GO:0015629; G	K05700	intron_variant	MODIFIER	c.61-77A>G					
XP_015772562.	XM_015917076	aug_v2a.02792	#N/D	#N/D	intron_variant	MODIFIER	c.36-22172T>C					

XP_015780817.:	XM_015925331	aug_v2a.06122	GO:0016021	K07380	intron_variant	MODIFIER	c.1147+847T>C	
XP_015773377.:	XM_015917891	aug_v2a.16851	GO:0005634; G(	K04682	intron_variant	MODIFIER	c.231-158T>A	
XP_015773395.:	XM_015917909	aug_v2a.12812	#N/D	#N/D	intron_variant	MODIFIER	c.1613-728T>C	
XP_015773443.:	XM_015917957	aug_v2a.05097	GO:0048786; G(	K14803	intron_variant	MODIFIER	c.686-96T>C	
XP_015773509.:	XM_015918023	aug_v2a.23754	GO:0016021; G(	K08465	intron_variant	MODIFIER	c.1063-196A>C	
XP_015771534.:	XM_015916048	aug_v2a.05253	GO:0016324; G(	#N/D	intron_variant	MODIFIER	c.81+4058A>T	
XP_015752125.:	XM_015896639	aug_v2a.02830	GO:0016021; G(	K04987	intron_variant	MODIFIER	c.3442-177A>G	
XP_015774341.:	XM_015918855	aug_v2a.11078	GO:0005794; G(	K08874	intron_variant	MODIFIER	c.2836-40T>C	
XP_015774551.:	XM_015919065	aug_v2a.05342	#N/D	#N/D	intron_variant	MODIFIER	c.110+3979A>G	
XP_015748571.:	XM_015893085	aug_v2a.23754	GO:0016021; G(	K08465	intron_variant	MODIFIER	c.-180+307T>C	
XP_015775080.:	XM_015919594	aug_v2a.10768	GO:0005096; G(	#N/D	intron_variant	MODIFIER	c.1810+4634G>C	
XP_015775208.:	XM_015919722	aug_v2a.11976	GO:0005576; G(	K14616	intron_variant	MODIFIER	c.1064-321A>C	
XP_015775348.:	XM_015919862	aug_v2a.12680	GO:0005576; G(	#N/D	intron_variant	MODIFIER	c.-39-2696T>G	
XP_015775476.:	XM_015919990	aug_v2a.09570	GO:0016021; G(	K14315	intron_variant	MODIFIER	c.1318-85C>G	
XP_015770781.:	XM_015915295	aug_v2a.12439	GO:0016021; G(	K04988	intron_variant	MODIFIER	c.638+134T>C	
XP_015775823.:	XM_015920337	aug_v2a.12763	#N/D	#N/D	intron_variant	MODIFIER	c.1248-2021A>G	
XP_015776386.:	XM_015920900	aug_v2a.18202	GO:0005783; G(	K09866	intron_variant	MODIFIER	c.1020+266A>G	
XP_015776505.:	XM_015921019	aug_v2a.11224	GO:0031012; G(	#N/D	intron_variant	MODIFIER	c.2470+405T>A	
XP_015773513.:	XM_015918027	aug_v2a.00401	GO:0030424; G(	K04594	3_prime_UTR_variant	MODIFIER	c.*3T>C	3
XP_015758694.:	XM_015903208	aug_v2a.24129	GO:0031410; G(	K10356	intron_variant	MODIFIER	c.2741+941A>G	
XP_015778358.:	XM_015922872	aug_v2a.14238	GO:0005887; G(	K03320	intron_variant	MODIFIER	c.-31+1339T>A	
XP_015754101.:	XM_015898615	aug_v2a.15790	GO:0030054; G(	K04615	intron_variant	MODIFIER	c.1664+404A>C	
XP_015778550.:	XM_015923064	aug_v2a.00480	GO:0005737; G(	K01025	intron_variant	MODIFIER	c.594+69A>T	
XP_015779430.:	XM_015923944	aug_v2a.16031	GO:0016021	#N/D	intron_variant	MODIFIER	c.470-154A>G	
XP_015779566.:	XM_015924080	aug_v2a.20129	GO:0005634; G(	#N/D	intron_variant	MODIFIER	c.27-83A>G	
XP_015758162.:	XM_015902676	aug_v2a.06441	GO:0030424; G(	K05126	intron_variant	MODIFIER	c.783+314C>G	
#N/D	XR_001560406.:	aug_v2a.13508	GO:0031012; G(	#N/D	intron_variant	MODIFIER	n.762-78A>T	
#N/D	XR_001563270.:	#N/D	#N/D	#N/D	intron_variant	MODIFIER	n.472-2678A>G	
#N/D	XR_001564224.:	aug_v2a.14365	GO:0016323; G(	K05665	intron_variant	MODIFIER	n.419-482A>G	
#N/D	XR_001564779.:	aug_v2a.09680	GO:0016021	#N/D	intron_variant	MODIFIER	n.443+43A>G	

#N/D	XR_001565393.	aug_v2a.08343	GO:0005737; G	K00461	intron_variant	MODIFIER	n.225-141T>A					
#N/D	XR_001565502.	#N/D	#N/D	#N/D	intron_variant	MODIFIER	n.990+3050A>G					
#N/D	XR_001565798.	aug_v2a.11999	GO:0030054; G	#N/D	intron_variant	MODIFIER	n.534+122T>C					
#N/D	XR_001565798.	aug_v2a.11999	GO:0030054; G	#N/D	intron_variant	MODIFIER	n.834+239T>C					
#N/D	#N/D	#N/D	#N/D	#N/D	intron_variant	MODIFIER	n.2384-119A>G					
XP_015755672.	XM_015900186	aug_v2a.05185	GO:0034703; G	K04984	intron_variant	MODIFIER	c.2061-332A>C					
XP_015761721.	XM_015906235	aug_v2a.01487	GO:0034451; G	K21765	synonymous_variant	LOW	c.639T>A	p.Thr213Thr	979/1927	639/906	213/301	
XP_015773521.	XM_015918035	aug_v2a.04308	#N/D	#N/D	intron_variant	MODIFIER	c.1626+19043T>C					
XP_015773521.	XM_015918035	aug_v2a.04308	#N/D	#N/D	intron_variant	MODIFIER	c.1113+139A>G					
XP_015773521.	XM_015918035	aug_v2a.04308	#N/D	#N/D	intron_variant	MODIFIER	c.1114-6376G>C					
XP_015747200.	XM_015891714	aug_v2a.06768	GO:0000166 GC	K09680	missense_variant	MODERATE	c.1757A>C	p.Asp586Ala	1882/2188	1757/1932	586/643	
XP_015752866.	XM_015897380	aug_v2a.02856	#N/D	#N/D	missense_variant	MODERATE	c.1189A>G	p.Thr397Ala	1306/2175	1189/2058	397/685	
XP_015753605.	XM_015898119	aug_v2a.20275	GO:0001756 GC	K05463	missense_variant	MODERATE	c.20A>C	p.Gln7Pro	289/1340	20/834	7/277	
XP_015753654.	XM_015898168	aug_v2a.04794	GO:0005737 GC	K19363	missense_variant	MODERATE	c.218A>G	p.Glu73Gly	299/768	218/438	73/145	
XP_015765531.	XM_015910045	aug_v2a.04222	#N/D	K23368	intron_variant	MODIFIER	c.1392+102T>G					
XP_015774694.	XM_015919208	aug_v2a.06220	#N/D	#N/D	intron_variant	MODIFIER	c.208-58A>G					
XP_015758104.	XM_015902618	aug_v2a.06443	#N/D	#N/D	intron_variant	MODIFIER	c.466-78A>G					
XP_015758965.	XM_015903479	aug_v2a.13150	GO:0000166 GC	K02324	missense_variant	MODERATE	c.4864T>A	p.Tyr1622Asn	5052/6921	4864/6456	1622/2151	
XP_015760268.	XM_015904782	aug_v2a.18643	#N/D	K20478	missense_variant	MODERATE	c.6498A>C	p.Glu2166Asp	6550/9051	6498/8493	2166/2830	
XP_015763164.	XM_015907678	aug_v2a.06181	GO:0003682 GC	K17495	missense_variant	MODERATE	c.428A>G	p.Asn143Ser	428/1062	428/1062	143/353	
XP_015764021.	XM_015908535	aug_v2a.09307	GO:0005506 GC	K16914	missense_variant	MODERATE	c.313A>T	p.Met105Leu	358/501	313/456	105/151	
XP_015764558.	XM_015909072	aug_v2a.17807	GO:0000166 GC	K13431	missense_variant	MODERATE	c.337A>G	p.Thr113Ala	629/1777	337/1416	113/471	
XP_015774694.	XM_015919208	aug_v2a.06220	#N/D	#N/D	5_prime_UTR_variant	MODIFIER	c.-470G>C					470
XP_015766225.	XM_015910739	aug_v2a.01852	GO:0005515 GC	K12035	missense_variant	MODERATE	c.497T>A	p.Leu166Gln	1419/2581	497/1659	166/552	
#N/D	XR_001563374.	#N/D	#N/D	#N/D	intron_variant	MODIFIER	n.930+2361T>C					
XP_015768623.	XM_015913137	aug_v2a.16918	GO:0005515 GC	#N/D	missense_variant	MODERATE	c.966T>G	p.Ser322Arg	1718/3386	966/2634	322/877	
XP_015770562.	XM_015915076	aug_v2a.13885	#N/D	#N/D	missense_variant	MODERATE	c.1006A>G	p.Asn336Asp	1036/8855	1006/8361	336/2786	
#N/D	#N/D	#N/D	#N/D	#N/D	downstream_gene_vari	MODIFIER	c.*4512A>G					2263
XP_015775797.	XM_015920311	aug_v2a.01757	GO:0003779 GC	K10457	missense_variant	MODERATE	c.768A>C	p.Lys256Asn	802/1894	768/1860	256/619	
XP_015779520.	XM_015924034	aug_v2a.20978	#N/D	#N/D	missense_variant	MODERATE	c.922A>G	p.Thr308Ala	1140/4631	922/2526	308/841	

XP_015780155.	XM_015924669	aug_v2a.07934	GO:0003824 GC	K16608	missense_variant	MODERATE	c.1033T>C	p.Tyr345His	1288/5596	1033/3066	345/1021	
XP_015780750.	XM_015925264	aug_v2a.22215	#N/D	#N/D	missense_variant	MODERATE	c.29T>A	p.Leu10His	87/1643	29/396	10/131	
#N/D	#N/D	#N/D	#N/D	#N/D	downstream_gene_vari	MODIFIER	n.*1629T>G					1629
XP_015754059.	XM_015898573	aug_v2a.20102	GO:0003417 GC	K04659	missense_variant&splic	MODERATE	c.1921T>A	p.Cys641Ser	1921/3621	1921/3606	641/1201	
#N/D	#N/D	#N/D	#N/D	#N/D	non_coding_transcript_	MODIFIER	n.1000T>G					
#N/D	#N/D	#N/D	#N/D	#N/D	downstream_gene_vari	MODIFIER	n.*2824T>G					2824
#N/D	#N/D	#N/D	#N/D	#N/D	downstream_gene_vari	MODIFIER	c.*7469T>C					3755
XP_015755542.	XM_015900056	aug_v2a.10189	GO:0005604; G	K17341	splice_region_variant&i	LOW	c.1166-4T>G					
XP_015761884.	XM_015906398	aug_v2a.11480	GO:0031410; G	K18152	splice_region_variant&i	LOW	c.378-4T>C					
#N/D	#N/D	#N/D	#N/D	#N/D	downstream_gene_vari	MODIFIER	n.*7577A>G					4926
#N/D	XR_001565228.	#N/D	#N/D	#N/D	splice_region_variant&i	LOW	n.230T>G					
#N/D	#N/D	#N/D	#N/D	#N/D	downstream_gene_vari	MODIFIER	c.*2232T>C					1974
XP_015761332.	XM_015905846	aug_v2a.05883	#N/D	#N/D	intron_variant	MODIFIER	c.528+18C>G					
XP_015758578.	XM_015903092	aug_v2a.11007	#N/D	#N/D	intron_variant	MODIFIER	c.806-137T>C					
XP_015748242.	XM_015892756	aug_v2a.05207	GO:0000166 GC	K05126	missense_variant	MODERATE	c.2613A>G	p.Ile871Met	3084/4530	2613/3036	871/1011	
XP_015770883.	XM_015915397	aug_v2a.07682	GO:0003677	K20478	missense_variant	MODERATE	c.10352A>C	p.Asn3451Thr	10362/11892	10352/11754	3451/3917	
XP_015758531.	XM_015903045	aug_v2a.11004	GO:0004177 GC	K01255	missense_variant	MODERATE	c.755G>C	p.Gly252Ala	813/1954	755/1548	252/515	
XP_015780340.	XM_015924854	aug_v2a.05409	GO:0004719 GC	K00573	missense_variant	MODERATE	c.301T>C	p.Ser101Pro	301/312	301/312	101/103	
XP_015754070.	XM_015898584	aug_v2a.14607	GO:0005515 GC	K06838	missense_variant	MODERATE	c.4333C>G	p.Gln1445Glu	4426/4986	4333/4872	1445/1623	
XP_015767252.	XM_015911766	aug_v2a.11374	GO:0016021	#N/D	missense_variant	MODERATE	c.785A>G	p.Lys262Arg	850/1655	785/1590	262/529	
XP_015749426.	XM_015893940	aug_v2a.06213	#N/D	#N/D	missense_variant	MODERATE	c.649A>G	p.Met217Val	912/4827	649/4539	217/1512	
XP_015761884.	XM_015906398	aug_v2a.11480	GO:0031410; G	K18152	splice_region_variant&i	LOW	c.378-4T>C					
XP_015758440.	XM_015902954	aug_v2a.24500	GO:0000166 GC	K15456	synonymous_variant	LOW	c.48A>G	p.Lys16Lys	136/1214	48/546	16/181	
XP_015770867.	XM_015915381	aug_v2a.02871	GO:0000187 GC	#N/D	synonymous_variant	LOW	c.2098A>G	p.Ala700Ala	3564/5699	2098/3549	700/1182	
#N/D	#N/D	#N/D	#N/D	#N/D	intergenic_region	MODIFIER	n.1323058A>G					
#N/D	#N/D	#N/D	#N/D	#N/D	intergenic_region	MODIFIER	n.219233A>C					
#N/D	#N/D	#N/D	#N/D	#N/D	intergenic_region	MODIFIER	n.530855C>G					
XP_015754975.	XM_015899489	aug_v2a.19073	GO:0003824 GC	K10147	synonymous_variant	LOW	c.1044T>C	p.Ser348Ser	1164/1842	1044/1215	348/404	
XP_015768541.	XM_015913055	aug_v2a.10224	GO:0003824 GC	#N/D	synonymous_variant	LOW	c.285A>G	p.Glu95Glu	321/1050	285/999	95/332	
XP_015777776.	XM_015922290	aug_v2a.03038	GO:0004197 GC	K11840	synonymous_variant	LOW	c.2580T>C	p.Ala860Ala	2893/8599	2580/6969	860/2322	
XP_015763165.	XM_015907679	aug_v2a.06182	GO:0004842 GC	K10595	synonymous_variant	LOW	c.5097A>G	p.Arg1699Arg	5232/11573	5097/11187	1699/3728	
XP_015756276.	XM_015900790	aug_v2a.00421	GO:0004871 GC	K16506	synonymous_variant	LOW	c.5286A>G	p.Val1762Val	5533/7717	5286/7470	1762/2489	

XP_015773509.:	XM_015918023	aug_v2a.23754	GO:0004888 GC	K08465	synonymous_variant	LOW	c.381T>C	p.Ile127Ile	381/1911	381/1323	127/440
XP_015773375.:	XM_015917889	aug_v2a.07476	GO:0005215 GC	K12391	synonymous_variant	LOW	c.462A>G	p.Arg154Arg	491/2757	462/2358	154/785
XP_015765276.:	XM_015909790	aug_v2a.06108	GO:0005215 GC	#N/D	synonymous_variant	LOW	c.585T>C	p.Ser195Ser	734/2005	585/1557	195/518
XP_015765276.:	XM_015909790	aug_v2a.06108	GO:0005215 GC	#N/D	synonymous_variant	LOW	c.165A>G	p.Val55Val	255/1940	165/1551	55/516
XP_015754993.:	XM_015899507	aug_v2a.10966	GO:0005215 GC	K14638	synonymous_variant	LOW	c.246A>G	p.Leu82Leu	1116/2811	246/1554	82/517
XP_015754993.:	XM_015899507	aug_v2a.10966	GO:0005215 GC	K14638	synonymous_variant	LOW	c.867T>C	p.Ser289Ser	1737/2811	867/1554	289/517
XP_015749068.:	XM_015893582	aug_v2a.17185	GO:0005778; G(	K13338	synonymous_variant	LOW	c.671T>G	p.Ser224Ser	791/2512	671/2307	224/768
XP_015751474.:	XM_015895988	#N/D	#N/D	#N/D	synonymous_variant	LOW	c.471G>C	p.Ser157Ser	525/2338	471/1209	157/402
XP_015751698.:	XM_015896212	aug_v2a.01037	GO:0003993 GC	K14379	synonymous_variant	LOW	c.1002A>G	p.Ser334Ser	1139/1285	1002/1008	334/335
XP_015760757.:	XM_015905271	aug_v2a.24586	GO:0005488 GC	#N/D	synonymous_variant	LOW	c.147C>G	p.Leu49Leu	481/10573	147/9609	49/3202
XP_015752405.:	XM_015896919	aug_v2a.15260	GO:0000077 GC	K04728	synonymous_variant	LOW	c.4469C>G	p.Gly1490Gly	4604/10358	4469/10173	1490/3390
XP_015752454.:	XM_015896968	aug_v2a.14945	GO:0005509 GC	#N/D	synonymous_variant	LOW	c.297A>G	p.Lys99Lys	446/2065	297/966	99/321
XP_015752845.:	XM_015897359	aug_v2a.08393	GO:0005102 GC	K06252	synonymous_variant	LOW	c.7487G>C	p.Ala2496Ala	7812/16621	7487/16296	2496/5431
XP_015752845.:	XM_015897359	aug_v2a.08393	GO:0005102 GC	K06252	synonymous_variant	LOW	c.3276A>G	p.Lys1092Lys	3601/16621	3276/16296	1092/5431
XP_015748949.:	XM_015893463	aug_v2a.19052	#N/D	#N/D	intron_variant	MODIFIER	c.306+113T>C				
XP_015753209.:	XM_015897723	aug_v2a.04432	GO:0003677 GC	K09309	synonymous_variant	LOW	c.528T>A	p.Val176Val	794/1521	528/708	176/235
XP_015753231.:	XM_015897745	aug_v2a.24050	GO:0006810 GC	K14713	synonymous_variant	LOW	c.540A>G	p.Glu180Glu	598/1320	540/1167	180/388
XP_015753552.:	XM_015898066	#N/D	#N/D	K04255	synonymous_variant	LOW	c.893T>G	p.Cys298Cys	893/996	893/996	298/331
XP_015753634.:	XM_015898148	aug_v2a.04793	GO:0005215 GC	K13856	synonymous_variant	LOW	c.699T>C	p.Ser233Ser	2559/5740	699/3057	233/1018
XP_015753887.:	XM_015898401	aug_v2a.06186	GO:0004091 GC	K12417	synonymous_variant	LOW	c.124T>G	p.Ala42Ala	865/2578	124/1656	42/551
XP_015753889.:	XM_015898403	aug_v2a.16663	#N/D	#N/D	synonymous_variant	LOW	c.508A>G	p.Glu170Glu	526/2789	508/1443	170/480
XP_015754058.:	XM_015898572	aug_v2a.15791	GO:0005576 GC	K13110	synonymous_variant	LOW	c.822A>G	p.Thr274Thr	924/1715	822/1062	274/353
XP_015752949.:	XM_015897463	aug_v2a.21812	GO:0005488 GC	#N/D	synonymous_variant	LOW	c.567A>G	p.Ala189Ala	637/2554	567/1683	189/560
XP_015754310.:	XM_015898824	aug_v2a.12941	GO:0000036 GC	K12442	synonymous_variant	LOW	c.7831A>G	p.Val2611Val	8481/12017	7831/10746	2611/3581
XP_015754672.:	XM_015899186	aug_v2a.06739	GO:0003774 GC	#N/D	synonymous_variant	LOW	c.2473T>C	p.Arg825Arg	3121/4596	2473/3336	825/1111
XP_015754739.:	XM_015899253	aug_v2a.04852	GO:0005515 GC	#N/D	synonymous_variant	LOW	c.597A>C	p.Val199Val	890/1073	597/765	199/254
XP_015756278.:	XM_015900792	aug_v2a.19517	GO:0005509 GC	K16506	synonymous_variant	LOW	c.5117A>T	p.Val1706Val	5211/18466	5117/18372	1706/6123
XP_015755639.:	XM_015900153	aug_v2a.12414	GO:0005515 GC	K14554	synonymous_variant	LOW	c.1768A>G	p.Val590Val	1796/3533	1768/2568	590/855
XP_015755682.:	XM_015900196	aug_v2a.18180	GO:0003674 GC	#N/D	synonymous_variant	LOW	c.127A>G	p.Asp43Asp	311/1749	127/1491	43/496
XP_015756581.:	XM_015901095	#N/D	#N/D	#N/D	synonymous_variant	LOW	c.18C>G	p.Arg6Arg	116/1291	18/669	6/222
XP_015771047.:	XM_015915561	aug_v2a.14067	#N/D	#N/D	intron_variant	MODIFIER	c.196-46C>G				
XP_015757439.:	XM_015901953	aug_v2a.06864	GO:0016020 GC	#N/D	synonymous_variant	LOW	c.1280A>T	p.Phe427Phe	1565/2595	1280/1473	427/490

XP_015773395.	XM_015917909	aug_v2a.12812	#N/D	#N/D	intron_variant	MODIFIER	c.2456+97C>G				
XP_015754070.	XM_015898584	aug_v2a.14607	GO:0005515 GC	K06838	synonymous_variant	LOW	c.1743T>C	p.Phe581Phe	1836/4986	1743/4872	581/1623
XP_015757653.	XM_015902167	aug_v2a.00176	GO:0099078; G(	K04232	synonymous_variant	LOW	c.228A>C	p.Gly76Gly	228/1777	228/1077	76/358
XP_015758092.	XM_015902606	aug_v2a.11337	GO:0005488 GC	K11824	synonymous_variant	LOW	c.30T>C	p.Arg10Arg	304/2424	30/2150	10/715
XP_015758138.	XM_015902652	aug_v2a.23780	#N/D	#N/D	synonymous_variant	LOW	c.4631T>G	p.Arg1544Arg	4721/7706	4631/6882	1544/2293
XP_015758374.	XM_015902888	aug_v2a.12636	GO:0005509 GC	K17095	synonymous_variant	LOW	c.721A>G	p.Gly241Gly	1420/3051	721/978	241/325
XP_015758599.	XM_015903113	aug_v2a.22017	GO:0000166 GC	K17915	synonymous_variant	LOW	c.177T>C	p.Cys59Cys	216/1193	177/1128	59/375
XP_015769665.	XM_015914179	aug_v2a.10497	GO:0005515 GC	K12604	synonymous_variant	LOW	c.3894T>C	p.Asn1298Asn	4087/7702	3894/7332	1298/2443
XP_015761711.	XM_015906225	aug_v2a.01492	GO:0005634 GC	#N/D	synonymous_variant	LOW	c.288T>C	p.Tyr96Tyr	412/912	288/348	96/115
XP_015758626.	XM_015903140	aug_v2a.09757	GO:0000166 GC	K12812	synonymous_variant	LOW	c.312T>C	p.Thr104Thr	440/1625	312/1290	104/429
XP_015759029.	XM_015903543	aug_v2a.12924	GO:0016021; G(	#N/D	synonymous_variant	LOW	c.2334T>C	p.Val778Val	2445/4432	2334/3777	778/1258
XP_015759989.	XM_015904503	aug_v2a.02347	GO:0045179; G(	#N/D	synonymous_variant	LOW	c.621A>G	p.Gly207Gly	756/1642	621/1128	207/375
XP_015760867.	XM_015905381	aug_v2a.23959	GO:0003674 GC	K06768	synonymous_variant	LOW	c.206A>G	p.Trp69Trp	207/2241	206/1836	69/611
XP_015760869.	XM_015905383	aug_v2a.22167	GO:0003676; G(	#N/D	synonymous_variant	LOW	c.549G>C	p.Ser183Ser	880/7192	549/6177	183/2058
XP_015761441.	XM_015905955	aug_v2a.15867	GO:0000166 GC	K03979	synonymous_variant	LOW	c.1857T>G	p.Leu619Leu	1894/2563	1857/2028	619/675
XP_015761517.	XM_015906031	#N/D	#N/D	#N/D	synonymous_variant	LOW	c.1065T>C	p.Ile355Ile	1096/1974	1065/1446	355/481
XP_015761517.	XM_015906031	#N/D	#N/D	#N/D	synonymous_variant	LOW	c.462C>G	p.Thr154Thr	493/1974	462/1446	154/481
XP_015761517.	XM_015906031	#N/D	#N/D	#N/D	synonymous_variant	LOW	c.1212T>C	p.Ser404Ser	1243/1974	1212/1446	404/481
XP_015761961.	XM_015906475	#N/D	#N/D	K01131	synonymous_variant	LOW	c.252T>G	p.Leu84Leu	258/1956	252/1557	84/518
XP_015762163.	XM_015906677	aug_v2a.18390	GO:0000287 GC	K03515	synonymous_variant	LOW	c.810A>C	p.Asn270Asn	882/3144	810/3057	270/1018
XP_015762577.	XM_015907091	aug_v2a.07128	GO:0005634; G(	K12605	synonymous_variant	LOW	c.222T>C	p.Pro74Pro	222/795	222/681	74/226
XP_015762265.	XM_015906779	aug_v2a.05040	GO:0005083 GC	K20223	synonymous_variant	LOW	c.1475T>C	p.Pro492Pro	1605/3633	1475/3021	492/1006
XP_015762278.	XM_015906792	aug_v2a.09010	#N/D	K20478	synonymous_variant	LOW	c.5062T>C	p.Leu1688Leu	6049/11900	5062/10374	1688/3457
XP_015764002.	XM_015908516	aug_v2a.09304	GO:0004871 GC	K02842	synonymous_variant	LOW	c.162T>C	p.Pro54Pro	1929/4871	162/1956	54/651
XP_015764677.	XM_015909191	aug_v2a.09255	#N/D	#N/D	synonymous_variant	LOW	c.534T>C	p.Ser178Ser	534/980	534/651	178/216
XP_015764921.	XM_015909435	aug_v2a.17161	GO:0003824 GC	K01192	synonymous_variant	LOW	c.541T>C	p.Arg181Arg	578/2297	541/2187	181/728
XP_015765242.	XM_015909756	#N/D	#N/D	#N/D	missense_variant	MODERATE	c.437C>G	p.Ser146Trp	669/1487	437/717	146/238
XP_015764940.	XM_015909454	aug_v2a.05493	GO:0003723	#N/D	synonymous_variant	LOW	c.573T>C	p.Leu191Leu	1383/2467	573/1248	191/415
XP_015764975.	XM_015909489	aug_v2a.05494	GO:0000166 GC	K14947	synonymous_variant	LOW	c.1749A>G	p.Lys583Lys	1771/2499	1749/2097	583/698
XP_015766287.	XM_015910801	aug_v2a.01851	GO:0005515 GC	#N/D	synonymous_variant	LOW	c.2025T>C	p.Cys675Cys	3272/4257	2025/2268	675/755
XP_015765170.	XM_015909684	aug_v2a.08778	GO:0016021; G(	K23088	synonymous_variant	LOW	c.916T>A	p.Ile306Ile	931/2154	916/1812	306/603
XP_015766287.	XM_015910801	aug_v2a.01851	GO:0005515 GC	#N/D	synonymous_variant	LOW	c.507A>G	p.Glu169Glu	1754/4257	507/2268	169/755
XP_015766297.	XM_015910811	aug_v2a.23025	#N/D	#N/D	synonymous_variant	LOW	c.2792T>C	p.Ser931Ser	2837/3510	2792/3465	931/1154
XP_015766507.	XM_015911021	aug_v2a.00758	GO:0005216 GC	K04988	synonymous_variant	LOW	c.4314T>C	p.His1438His	4442/8365	4314/7974	1438/2657

XP_015766857.:	XM_015911371	aug_v2a.11511	GO:0016021	#N/D	synonymous_variant	LOW	c.973C>G	p.Val325Val	1195/2067	973/1176	325/391
XP_015767853.:	XM_015912367	aug_v2a.21960	GO:0000166 GC	K07209	synonymous_variant	LOW	c.48A>G	p.Lys16Lys	48/887	48/801	16/266
XP_015768229.:	XM_015912743	aug_v2a.20021	GO:0001843 GC	K07207	synonymous_variant	LOW	c.3549T>C	p.Ser1183Ser	3639/4512	3549/4251	1183/1416
XP_015768430.:	XM_015912944	aug_v2a.21854	GO:0000723 GC	K11109	synonymous_variant	LOW	c.298T>C	p.Arg100Arg	346/2781	298/2442	100/813
XP_015768478.:	XM_015912992	#N/D	#N/D	#N/D	synonymous_variant	LOW	c.427T>C	p.His143His	897/996	427/459	143/152
XP_015768595.:	XM_015913109	aug_v2a.22790	#N/D	K04377	synonymous_variant	LOW	c.120T>C	p.Leu40Leu	2257/4005	120/1050	40/349
XP_015768650.:	XM_015913164	#N/D	#N/D	#N/D	synonymous_variant	LOW	c.926T>C	p.Ala309Ala	1358/3133	926/2523	309/840
XP_015768940.:	XM_015913454	aug_v2a.10187	GO:0004867 GC	K06238	synonymous_variant	LOW	c.1547T>C	p.Ala516Ala	2733/5701	1547/4515	516/1504
XP_015769616.:	XM_015914130	aug_v2a.01755	GO:0005634; G(	K11093	synonymous_variant	LOW	c.927A>G	p.Arg309Arg	1063/1962	927/1095	309/364
XP_015769652.:	XM_015914166	aug_v2a.10630	GO:0005085 GC	#N/D	synonymous_variant	LOW	c.1024A>C	p.His342His	1947/4888	1024/3408	342/1135
XP_015769668.:	XM_015914182	aug_v2a.17148	GO:0003824 GC	K00815	synonymous_variant	LOW	c.825A>T	p.Ser275Ser	1999/2327	825/1128	275/375
XP_015770244.:	XM_015914758	aug_v2a.02318	GO:0000724 GC	K10484	synonymous_variant	LOW	c.508A>G	p.Val170Val	624/821	508/705	170/234
XP_015770272.:	XM_015914786	aug_v2a.02321	GO:0001906 GC	K15720	synonymous_variant	LOW	c.1141T>C	p.Arg381Arg	1906/4456	1141/3240	381/1079
XP_015748829.:	XM_015893343	#N/D	#N/D	#N/D	synonymous_variant	LOW	c.1249T>C	p.Pro417Pro	2273/3721	1249/2697	417/898
XP_015770557.:	XM_015915071	aug_v2a.19400	GO:0008168	#N/D	synonymous_variant	LOW	c.251T>C	p.Ala84Ala	478/1038	251/606	84/201
XP_015770563.:	XM_015915077	aug_v2a.13885	#N/D	#N/D	synonymous_variant	LOW	c.2959T>G	p.Val987Val	2989/8855	2959/8361	987/2786
XP_015770578.:	XM_015915092	aug_v2a.19402	GO:0000166 GC	#N/D	synonymous_variant	LOW	c.198T>C	p.Tyr66Tyr	543/1139	198/564	66/187
XP_015770789.:	XM_015915303	#N/D	#N/D	K08187	synonymous_variant	LOW	c.876T>C	p.Ser292Ser	904/1114	876/1086	292/361
XP_015771532.:	XM_015916046	aug_v2a.11297	GO:0001701 GC	K23404	synonymous_variant	LOW	c.1531T>C	p.Pro511Pro	1563/1805	1531/1713	511/570
XP_015757315.:	XM_015901829	aug_v2a.05536	#N/D	K20478	synonymous_variant	LOW	c.936A>T	p.Ile312Ile	1605/17880	936/17211	312/5736
XP_015765285.:	XM_015909799	aug_v2a.06106	#N/D	K08202	synonymous_variant	LOW	c.28C>G	p.Glu10Glu	188/1483	28/1308	10/435
XP_015771657.:	XM_015916171	aug_v2a.07386	GO:0003824 GC	#N/D	synonymous_variant	LOW	c.184T>C	p.Pro62Pro	234/563	184/432	62/143
XP_015771962.:	XM_015916476	aug_v2a.11999	GO:0004872 GC	#N/D	synonymous_variant	LOW	c.1098A>C	p.Ser366Ser	1123/1935	1098/1563	366/520
XP_015771990.:	XM_015916504	aug_v2a.18502	GO:0004720 GC	K00280	synonymous_variant	LOW	c.135A>C	p.Gly45Gly	569/2533	135/1209	45/402
XP_015772024.:	XM_015916538	#N/D	#N/D	K08375	synonymous_variant	LOW	c.491T>C	p.Ser164Ser	741/1599	491/1068	164/355
XP_015773220.:	XM_015917734	aug_v2a.11490	#N/D	K20283	synonymous_variant	LOW	c.3318T>C	p.His1106His	3359/5556	3318/5124	1106/1707
XP_015773220.:	XM_015917734	aug_v2a.11490	#N/D	K20283	synonymous_variant	LOW	c.985C>G	p.Glu329Glu	1026/5556	985/5124	329/1707
XP_015773372.:	XM_015917886	aug_v2a.12816	GO:0000166 GC	K22399	synonymous_variant	LOW	c.879T>A	p.Leu293Leu	1014/1460	879/1086	293/361
XP_015773913.:	XM_015918427	aug_v2a.13253	GO:0005515 GC	#N/D	synonymous_variant	LOW	c.759A>T	p.Val253Val	1014/2471	759/1005	253/334
XP_015773967.:	XM_015918481	aug_v2a.14638	GO:0003824 GC	#N/D	synonymous_variant	LOW	c.720A>G	p.Ser240Ser	1198/2080	720/984	240/327
XP_015774405.:	XM_015918919	aug_v2a.01565	#N/D	K04203	synonymous_variant	LOW	c.687T>C	p.Leu229Leu	2363/3442	687/1065	229/354
XP_015775132.:	XM_015919646	aug_v2a.07618	GO:0000166 GC	K01869	synonymous_variant	LOW	c.2970T>C	p.His990His	3119/3257	2970/3093	990/1030
XP_015775431.:	XM_015919945	aug_v2a.15020	GO:0005829; G(	#N/D	synonymous_variant	LOW	c.1498C>G	p.Glu500Glu	1539/2180	1498/2139	500/712

XP_015775636.	XM_015920150	#N/D	#N/D	K05052	synonymous_variant	LOW	c.306T>C	p.Tyr102Tyr	313/1134	306/960	102/319	
XP_015765242.	XM_015909756	#N/D	#N/D	#N/D	synonymous_variant	LOW	c.598C>G	p.Glu200Glu	830/1487	598/717	200/238	
XP_015775797.	XM_015920311	aug_v2a.01757	GO:0003779 GC	K10457	synonymous_variant	LOW	c.1200T>C	p.Ile400Ile	1510/3391	1200/1902	400/633	
XP_015776469.	XM_015920983	aug_v2a.00797	GO:0006810 GC	K15381	synonymous_variant	LOW	c.973T>G	p.Gly325Gly	1205/1699	973/1416	325/471	
XP_015778630.	XM_015923144	aug_v2a.00310	#N/D	#N/D	synonymous_variant	LOW	c.936A>G	p.Gln312Gln	1163/1615	936/966	312/321	
XP_015778668.	XM_015923182	aug_v2a.22849	GO:0005829; G(	K12611	synonymous_variant	LOW	c.132A>G	p.Ala44Ala	164/1244	132/1197	44/398	
XP_015779068.	XM_015923582	aug_v2a.03194	GO:0005886 GC	K03327	synonymous_variant	LOW	c.393A>G	p.Arg131Arg	556/2519	393/1947	131/648	
XP_015779439.	XM_015923953	aug_v2a.00960	GO:0001714 GC	K08036	synonymous_variant	LOW	c.151A>G	p.Ala51Ala	525/2662	151/1596	51/531	
XP_015779624.	XM_015924138	aug_v2a.07299	GO:0016020 GC	#N/D	synonymous_variant	LOW	c.495T>C	p.Gly165Gly	1269/2414	495/1089	165/362	
XP_015779647.	XM_015924161	aug_v2a.05585	GO:0000070 GC	K06636	synonymous_variant	LOW	c.384G>C	p.Val128Val	510/1738	384/1053	128/350	
XP_015779873.	XM_015924387	aug_v2a.13949	GO:0000077 GC	K10867	synonymous_variant	LOW	c.1710A>G	p.Pro570Pro	1755/2815	1710/2589	570/862	
XP_015780138.	XM_015924652	aug_v2a.12430	GO:0000049 GC	K14288	synonymous_variant	LOW	c.81A>G	p.Glu27Glu	360/3972	81/2604	27/867	
XP_015780581.	XM_015925095	aug_v2a.00700	GO:0001614 GC	K05218	synonymous_variant	LOW	c.771T>G	p.Met257Met	837/2979	771/2130	257/709	
XP_015765286.	XM_015909800	aug_v2a.06106	#N/D	K08202	synonymous_variant	LOW	c.1032T>C	p.Pro344Pro	1192/1483	1032/1308	344/435	
XP_015773220.	XM_015917734	aug_v2a.11490	#N/D	K20283	synonymous_variant	LOW	c.1806A>G	p.Glu602Glu	1847/5556	1806/5124	602/1707	
XP_015780739.	XM_015925253	aug_v2a.10428	#N/D	#N/D	synonymous_variant	LOW	c.630T>C	p.Val210Val	661/1603	630/1509	210/502	
#N/D	#N/D	#N/D	#N/D	#N/D	upstream_gene_variant	MODIFIER	c.-6053A>G					24
#N/D	#N/D	#N/D	#N/D	#N/D	upstream_gene_variant	MODIFIER	n.-1755A>G					1755
#N/D	#N/D	#N/D	#N/D	#N/D	upstream_gene_variant	MODIFIER	c.-356T>A					190
#N/D	#N/D	#N/D	#N/D	#N/D	upstream_gene_variant	MODIFIER	n.-4490T>C					4490
#N/D	#N/D	#N/D	#N/D	#N/D	upstream_gene_variant	MODIFIER	n.-966A>G					966
#N/D	#N/D	#N/D	#N/D	#N/D	upstream_gene_variant	MODIFIER	c.-5086G>C					4986
#N/D	#N/D	#N/D	#N/D	#N/D	upstream_gene_variant	MODIFIER	c.-3607G>C					3607
#N/D	#N/D	#N/D	#N/D	#N/D	upstream_gene_variant	MODIFIER	c.-1316A>G					1036
#N/D	#N/D	#N/D	#N/D	#N/D	upstream_gene_variant	MODIFIER	c.-6115A>T					2857
#N/D	#N/D	#N/D	#N/D	#N/D	upstream_gene_variant	MODIFIER	n.-2332A>G					2332
#N/D	#N/D	#N/D	#N/D	#N/D	upstream_gene_variant	MODIFIER	c.-6231T>C					4071
#N/D	#N/D	#N/D	#N/D	#N/D	upstream_gene_variant	MODIFIER	c.-2431C>G					505
#N/D	#N/D	#N/D	#N/D	#N/D	upstream_gene_variant	MODIFIER	c.-42958A>T					2273
#N/D	#N/D	#N/D	#N/D	#N/D	upstream_gene_variant	MODIFIER	c.-548T>C					331
#N/D	#N/D	#N/D	#N/D	#N/D	upstream_gene_variant	MODIFIER	c.-1608A>C					985
#N/D	#N/D	#N/D	#N/D	#N/D	upstream_gene_variant	MODIFIER	c.-18019A>T					4978
#N/D	#N/D	#N/D	#N/D	#N/D	upstream_gene_variant	MODIFIER	n.-1310A>C					1310
#N/D	#N/D	#N/D	#N/D	#N/D	upstream_gene_variant	MODIFIER	c.-3850A>G					888

#N/D	#N/D	#N/D	#N/D	#N/D	upstream_gene_varian	MODIFIER	n.-1812T>A	1812
#N/D	#N/D	#N/D	#N/D	#N/D	upstream_gene_varian	MODIFIER	c.-3108T>C	3012
#N/D	#N/D	#N/D	#N/D	#N/D	upstream_gene_varian	MODIFIER	n.-3291T>C	3291
#N/D	#N/D	#N/D	#N/D	#N/D	upstream_gene_varian	MODIFIER	c.-3885T>C	3804
#N/D	#N/D	#N/D	#N/D	#N/D	upstream_gene_varian	MODIFIER	c.-7665A>C	3880
#N/D	#N/D	#N/D	#N/D	#N/D	upstream_gene_varian	MODIFIER	c.-4767A>G	4655
#N/D	#N/D	#N/D	#N/D	#N/D	upstream_gene_varian	MODIFIER	c.-17554A>T	2111
#N/D	#N/D	#N/D	#N/D	#N/D	upstream_gene_varian	MODIFIER	c.-14493T>C	3316
#N/D	#N/D	#N/D	#N/D	#N/D	upstream_gene_varian	MODIFIER	c.-12619A>C	729
#N/D	#N/D	#N/D	#N/D	#N/D	upstream_gene_varian	MODIFIER	c.-16623T>C	3223
#N/D	#N/D	#N/D	#N/D	#N/D	upstream_gene_varian	MODIFIER	n.-710T>C	710
#N/D	#N/D	#N/D	#N/D	#N/D	upstream_gene_varian	MODIFIER	c.-1951T>C	1916
#N/D	#N/D	#N/D	#N/D	#N/D	upstream_gene_varian	MODIFIER	c.-894A>G	894
#N/D	#N/D	#N/D	#N/D	#N/D	upstream_gene_varian	MODIFIER	c.-6939A>T	249
#N/D	#N/D	#N/D	#N/D	#N/D	upstream_gene_varian	MODIFIER	c.-2656T>C	2448
#N/D	#N/D	#N/D	#N/D	#N/D	upstream_gene_varian	MODIFIER	c.-4850A>C	4651
#N/D	#N/D	#N/D	#N/D	#N/D	upstream_gene_varian	MODIFIER	c.-4384A>C	4308
#N/D	#N/D	#N/D	#N/D	#N/D	upstream_gene_varian	MODIFIER	c.-5295T>C	3033
#N/D	#N/D	#N/D	#N/D	#N/D	upstream_gene_varian	MODIFIER	c.-1751T>C	1686
#N/D	#N/D	#N/D	#N/D	#N/D	upstream_gene_varian	MODIFIER	c.-3458A>G	2998
#N/D	#N/D	#N/D	#N/D	#N/D	upstream_gene_varian	MODIFIER	c.-9093A>G	357
#N/D	#N/D	#N/D	#N/D	#N/D	upstream_gene_varian	MODIFIER	c.-5648A>G	4988
#N/D	#N/D	#N/D	#N/D	#N/D	upstream_gene_varian	MODIFIER	c.-5126T>G	4525
#N/D	#N/D	#N/D	#N/D	#N/D	upstream_gene_varian	MODIFIER	c.-8640C>G	2652
#N/D	#N/D	#N/D	#N/D	#N/D	upstream_gene_varian	MODIFIER	c.-15002A>G	3810
#N/D	#N/D	#N/D	#N/D	#N/D	upstream_gene_varian	MODIFIER	c.-4009C>G	3979
#N/D	#N/D	#N/D	#N/D	#N/D	upstream_gene_varian	MODIFIER	c.-82A>G	41
#N/D	#N/D	#N/D	#N/D	#N/D	upstream_gene_varian	MODIFIER	c.-8184T>C	4644
#N/D	#N/D	#N/D	#N/D	#N/D	upstream_gene_varian	MODIFIER	c.-993G>C	981
#N/D	#N/D	#N/D	#N/D	#N/D	upstream_gene_varian	MODIFIER	c.-1267A>G	1056
#N/D	#N/D	#N/D	#N/D	#N/D	upstream_gene_varian	MODIFIER	n.-3151T>G	3151
#N/D	#N/D	#N/D	#N/D	#N/D	upstream_gene_varian	MODIFIER	c.-6678A>T	4070
#N/D	#N/D	#N/D	#N/D	#N/D	upstream_gene_varian	MODIFIER	n.-860A>G	860
#N/D	#N/D	#N/D	#N/D	#N/D	upstream_gene_varian	MODIFIER	c.-1309A>G	1298
#N/D	#N/D	#N/D	#N/D	#N/D	upstream_gene_varian	MODIFIER	c.-11963A>G	1603
#N/D	#N/D	#N/D	#N/D	#N/D	upstream_gene_varian	MODIFIER	c.-11103T>C	4568

#N/D	#N/D	#N/D	#N/D	#N/D	upstream_gene_variant	MODIFIER	c.-4209T>G	2443
#N/D	#N/D	#N/D	#N/D	#N/D	upstream_gene_variant	MODIFIER	c.-19696T>C	3161
#N/D	#N/D	#N/D	#N/D	#N/D	upstream_gene_variant	MODIFIER	c.-9234T>C	4582
#N/D	#N/D	#N/D	#N/D	#N/D	upstream_gene_variant	MODIFIER	c.-1003A>C	1003
#N/D	#N/D	#N/D	#N/D	#N/D	upstream_gene_variant	MODIFIER	c.-6240A>G	4572
#N/D	#N/D	#N/D	#N/D	#N/D	upstream_gene_variant	MODIFIER	c.-74557A>C	190
#N/D	#N/D	#N/D	#N/D	#N/D	upstream_gene_variant	MODIFIER	c.-13190T>C	1622
#N/D	#N/D	#N/D	#N/D	#N/D	upstream_gene_variant	MODIFIER	c.-4359G>C	3912
#N/D	#N/D	#N/D	#N/D	#N/D	upstream_gene_variant	MODIFIER	c.-3625G>C	3522
#N/D	#N/D	#N/D	#N/D	#N/D	upstream_gene_variant	MODIFIER	c.-8087A>C	3296
#N/D	#N/D	#N/D	#N/D	#N/D	upstream_gene_variant	MODIFIER	n.-1868A>C	1868
#N/D	#N/D	#N/D	#N/D	#N/D	upstream_gene_variant	MODIFIER	n.-2020T>C	2020
#N/D	#N/D	#N/D	#N/D	#N/D	upstream_gene_variant	MODIFIER	c.-39191A>C	2049
#N/D	#N/D	#N/D	#N/D	#N/D	upstream_gene_variant	MODIFIER	c.-7680T>A	4607
#N/D	#N/D	#N/D	#N/D	#N/D	upstream_gene_variant	MODIFIER	c.-2932T>A	2314
#N/D	#N/D	#N/D	#N/D	#N/D	upstream_gene_variant	MODIFIER	c.-3621T>C	2553
#N/D	#N/D	#N/D	#N/D	#N/D	upstream_gene_variant	MODIFIER	c.-3745A>G	2677
#N/D	#N/D	#N/D	#N/D	#N/D	upstream_gene_variant	MODIFIER	c.-4371A>G	3303
#N/D	#N/D	#N/D	#N/D	#N/D	upstream_gene_variant	MODIFIER	c.-5515G>C	4447
#N/D	#N/D	#N/D	#N/D	#N/D	upstream_gene_variant	MODIFIER	c.-28815A>G	543
#N/D	#N/D	#N/D	#N/D	#N/D	upstream_gene_variant	MODIFIER	c.-5967T>C	3698
#N/D	#N/D	#N/D	#N/D	#N/D	upstream_gene_variant	MODIFIER	c.-16669A>G	299
#N/D	#N/D	#N/D	#N/D	#N/D	upstream_gene_variant	MODIFIER	n.-2863A>G	212
#N/D	#N/D	#N/D	#N/D	#N/D	upstream_gene_variant	MODIFIER	c.-4548T>C	4434
#N/D	#N/D	#N/D	#N/D	#N/D	upstream_gene_variant	MODIFIER	c.-9750A>T	1279
#N/D	#N/D	#N/D	#N/D	#N/D	upstream_gene_variant	MODIFIER	c.-4933T>C	2799
#N/D	#N/D	#N/D	#N/D	#N/D	upstream_gene_variant	MODIFIER	c.-4457T>C	4441
#N/D	#N/D	#N/D	#N/D	#N/D	upstream_gene_variant	MODIFIER	c.-2976A>G	1050
#N/D	#N/D	#N/D	#N/D	#N/D	upstream_gene_variant	MODIFIER	n.-4970A>G	4970
#N/D	#N/D	#N/D	#N/D	#N/D	upstream_gene_variant	MODIFIER	c.-11719A>G	3374
#N/D	#N/D	#N/D	#N/D	#N/D	upstream_gene_variant	MODIFIER	c.-5827A>G	3667
#N/D	#N/D	#N/D	#N/D	#N/D	upstream_gene_variant	MODIFIER	c.-5070T>C	2643
#N/D	#N/D	#N/D	#N/D	#N/D	upstream_gene_variant	MODIFIER	n.-801T>C	801
#N/D	#N/D	#N/D	#N/D	#N/D	upstream_gene_variant	MODIFIER	c.-6834T>C	3038
#N/D	#N/D	#N/D	#N/D	#N/D	upstream_gene_variant	MODIFIER	c.-5289G>C	3020
#N/D	#N/D	#N/D	#N/D	#N/D	upstream_gene_variant	MODIFIER	c.-1063T>C	709

#N/D	#N/D	#N/D	#N/D	#N/D	upstream_gene_variant	MODIFIER	n.-2460A>G	2460
#N/D	#N/D	#N/D	#N/D	#N/D	upstream_gene_variant	MODIFIER	c.-3354A>G	392
#N/D	#N/D	#N/D	#N/D	#N/D	upstream_gene_variant	MODIFIER	c.-6138A>T	3176
#N/D	#N/D	#N/D	#N/D	#N/D	upstream_gene_variant	MODIFIER	c.-10723T>C	1987
#N/D	#N/D	#N/D	#N/D	#N/D	upstream_gene_variant	MODIFIER	c.-1363T>G	1363
#N/D	#N/D	#N/D	#N/D	#N/D	upstream_gene_variant	MODIFIER	c.-947T>G	616
#N/D	#N/D	#N/D	#N/D	#N/D	upstream_gene_variant	MODIFIER	c.-22445C>G	635
#N/D	#N/D	#N/D	#N/D	#N/D	upstream_gene_variant	MODIFIER	c.-25793A>G	4566
#N/D	#N/D	#N/D	#N/D	#N/D	upstream_gene_variant	MODIFIER	c.-644A>G	526
#N/D	#N/D	#N/D	#N/D	#N/D	upstream_gene_variant	MODIFIER	c.-3797A>C	2012
#N/D	#N/D	#N/D	#N/D	#N/D	upstream_gene_variant	MODIFIER	c.-10795T>C	1609
#N/D	#N/D	#N/D	#N/D	#N/D	upstream_gene_variant	MODIFIER	c.-12101A>G	2478
#N/D	#N/D	#N/D	#N/D	#N/D	upstream_gene_variant	MODIFIER	c.-4891T>C	4238
#N/D	#N/D	#N/D	#N/D	#N/D	upstream_gene_variant	MODIFIER	c.-8497T>C	4207
#N/D	#N/D	#N/D	#N/D	#N/D	upstream_gene_variant	MODIFIER	c.-4342A>C	4342
#N/D	#N/D	#N/D	#N/D	#N/D	upstream_gene_variant	MODIFIER	n.-4096T>G	4096
#N/D	#N/D	#N/D	#N/D	#N/D	upstream_gene_variant	MODIFIER	c.-8350C>G	39
#N/D	#N/D	#N/D	#N/D	#N/D	upstream_gene_variant	MODIFIER	c.-58717A>T	3997
#N/D	#N/D	#N/D	#N/D	#N/D	upstream_gene_variant	MODIFIER	c.-9578A>G	2797
#N/D	#N/D	#N/D	#N/D	#N/D	upstream_gene_variant	MODIFIER	c.-16018A>C	4736
#N/D	#N/D	#N/D	#N/D	#N/D	upstream_gene_variant	MODIFIER	c.-20824T>C	4482
#N/D	#N/D	#N/D	#N/D	#N/D	upstream_gene_variant	MODIFIER	c.-1452A>C	1256
#N/D	#N/D	#N/D	#N/D	#N/D	upstream_gene_variant	MODIFIER	n.-796T>C	796
#N/D	#N/D	#N/D	#N/D	#N/D	upstream_gene_variant	MODIFIER	c.-58528T>C	2573
#N/D	#N/D	#N/D	#N/D	#N/D	upstream_gene_variant	MODIFIER	c.-2913T>C	1093
#N/D	#N/D	#N/D	#N/D	#N/D	upstream_gene_variant	MODIFIER	c.-1511T>C	1437
#N/D	#N/D	#N/D	#N/D	#N/D	upstream_gene_variant	MODIFIER	c.-4392C>G	3448
#N/D	#N/D	#N/D	#N/D	#N/D	upstream_gene_variant	MODIFIER	c.-4239A>G	2201
#N/D	#N/D	#N/D	#N/D	#N/D	upstream_gene_variant	MODIFIER	n.-1600A>G	1600
#N/D	#N/D	#N/D	#N/D	#N/D	upstream_gene_variant	MODIFIER	c.-5404T>C	4996
#N/D	#N/D	#N/D	#N/D	#N/D	upstream_gene_variant	MODIFIER	c.-3340T>G	3308
#N/D	#N/D	#N/D	#N/D	#N/D	upstream_gene_variant	MODIFIER	n.-3447T>G	3447
#N/D	#N/D	#N/D	#N/D	#N/D	upstream_gene_variant	MODIFIER	c.-5054T>C	3923
#N/D	#N/D	#N/D	#N/D	#N/D	upstream_gene_variant	MODIFIER	c.-8118A>G	2920
#N/D	#N/D	#N/D	#N/D	#N/D	upstream_gene_variant	MODIFIER	c.-2585T>C	2548
#N/D	#N/D	#N/D	#N/D	#N/D	upstream_gene_variant	MODIFIER	c.-4014A>C	1913

#N/D	#N/D	#N/D	#N/D	#N/D	upstream_gene_variant	MODIFIER	c.-5401T>G	4348
#N/D	#N/D	#N/D	#N/D	#N/D	upstream_gene_variant	MODIFIER	c.-33328T>G	4109
#N/D	#N/D	#N/D	#N/D	#N/D	upstream_gene_variant	MODIFIER	c.-11049T>C	3183
#N/D	#N/D	#N/D	#N/D	#N/D	upstream_gene_variant	MODIFIER	c.-18048A>T	2605
#N/D	#N/D	#N/D	#N/D	#N/D	upstream_gene_variant	MODIFIER	c.-9085A>G	458
#N/D	#N/D	#N/D	#N/D	#N/D	upstream_gene_variant	MODIFIER	n.-57A>G	57
#N/D	#N/D	#N/D	#N/D	#N/D	upstream_gene_variant	MODIFIER	c.-4425T>C	2101
#N/D	#N/D	#N/D	#N/D	#N/D	upstream_gene_variant	MODIFIER	c.-57064T>C	179
#N/D	#N/D	#N/D	#N/D	#N/D	upstream_gene_variant	MODIFIER	c.-58910A>G	2025
#N/D	#N/D	#N/D	#N/D	#N/D	upstream_gene_variant	MODIFIER	c.-59257A>G	2372
#N/D	#N/D	#N/D	#N/D	#N/D	upstream_gene_variant	MODIFIER	c.-61733T>G	4848
#N/D	#N/D	#N/D	#N/D	#N/D	upstream_gene_variant	MODIFIER	c.-4874A>G	4790
#N/D	#N/D	#N/D	#N/D	#N/D	upstream_gene_variant	MODIFIER	c.-3409T>C	1048
#N/D	#N/D	#N/D	#N/D	#N/D	upstream_gene_variant	MODIFIER	c.-2277A>G	2277
#N/D	#N/D	#N/D	#N/D	#N/D	upstream_gene_variant	MODIFIER	c.-13704A>C	2076
#N/D	#N/D	#N/D	#N/D	#N/D	upstream_gene_variant	MODIFIER	c.-19192T>C	1045
#N/D	#N/D	#N/D	#N/D	#N/D	upstream_gene_variant	MODIFIER	c.-1207A>G	1130
#N/D	#N/D	#N/D	#N/D	#N/D	upstream_gene_variant	MODIFIER	c.-5007T>C	4968
#N/D	#N/D	#N/D	#N/D	#N/D	upstream_gene_variant	MODIFIER	c.-3051T>C	2073
#N/D	#N/D	#N/D	#N/D	#N/D	upstream_gene_variant	MODIFIER	c.-3223T>C	3190
#N/D	#N/D	#N/D	#N/D	#N/D	upstream_gene_variant	MODIFIER	c.-3112T>C	863
#N/D	#N/D	#N/D	#N/D	#N/D	upstream_gene_variant	MODIFIER	c.-1405A>G	1266
#N/D	#N/D	#N/D	#N/D	#N/D	upstream_gene_variant	MODIFIER	c.-4777A>G	1311
#N/D	#N/D	#N/D	#N/D	#N/D	upstream_gene_variant	MODIFIER	c.-8515A>G	3512
#N/D	#N/D	#N/D	#N/D	#N/D	upstream_gene_variant	MODIFIER	c.-3287A>G	3287
#N/D	#N/D	#N/D	#N/D	#N/D	upstream_gene_variant	MODIFIER	c.-3153T>C	2827
#N/D	#N/D	#N/D	#N/D	#N/D	upstream_gene_variant	MODIFIER	c.-4259A>G	4127
#N/D	#N/D	#N/D	#N/D	#N/D	upstream_gene_variant	MODIFIER	c.-468G>C	365
#N/D	#N/D	#N/D	#N/D	#N/D	upstream_gene_variant	MODIFIER	c.-5099T>C	3466
#N/D	#N/D	#N/D	#N/D	#N/D	upstream_gene_variant	MODIFIER	c.-1418T>C	1212
#N/D	#N/D	#N/D	#N/D	#N/D	upstream_gene_variant	MODIFIER	c.-11747A>T	2848
#N/D	#N/D	#N/D	#N/D	#N/D	upstream_gene_variant	MODIFIER	c.-11381C>G	4799
#N/D	#N/D	#N/D	#N/D	#N/D	upstream_gene_variant	MODIFIER	c.-860T>G	860
#N/D	#N/D	#N/D	#N/D	#N/D	upstream_gene_variant	MODIFIER	c.-22242A>G	2314
#N/D	#N/D	#N/D	#N/D	#N/D	upstream_gene_variant	MODIFIER	c.-5050T>G	3871
#N/D	#N/D	#N/D	#N/D	#N/D	upstream_gene_variant	MODIFIER	c.-8464A>G	2123

#N/D	#N/D	#N/D	#N/D	#N/D	upstream_gene_variant	MODIFIER	c.-1635A>T	389
#N/D	#N/D	#N/D	#N/D	#N/D	upstream_gene_variant	MODIFIER	c.-11754T>C	4383
#N/D	#N/D	#N/D	#N/D	#N/D	upstream_gene_variant	MODIFIER	c.-4277A>G	2628
#N/D	#N/D	#N/D	#N/D	#N/D	upstream_gene_variant	MODIFIER	c.-10895C>G	764
#N/D	#N/D	#N/D	#N/D	#N/D	upstream_gene_variant	MODIFIER	c.-1562T>C	1542
#N/D	#N/D	#N/D	#N/D	#N/D	upstream_gene_variant	MODIFIER	c.-5529A>G	2158
#N/D	#N/D	#N/D	#N/D	#N/D	upstream_gene_variant	MODIFIER	c.-3364A>C	2925
#N/D	#N/D	#N/D	#N/D	#N/D	upstream_gene_variant	MODIFIER	c.-30036T>C	1689
#N/D	#N/D	#N/D	#N/D	#N/D	upstream_gene_variant	MODIFIER	c.-872A>C	797
#N/D	#N/D	#N/D	#N/D	#N/D	upstream_gene_variant	MODIFIER	n.-1392A>G	1392
#N/D	#N/D	#N/D	#N/D	#N/D	upstream_gene_variant	MODIFIER	c.-589A>G	513
#N/D	#N/D	#N/D	#N/D	#N/D	upstream_gene_variant	MODIFIER	c.-22982T>C	1708
#N/D	#N/D	#N/D	#N/D	#N/D	upstream_gene_variant	MODIFIER	c.-3045A>G	2743
#N/D	#N/D	#N/D	#N/D	#N/D	upstream_gene_variant	MODIFIER	c.-228T>C	228
#N/D	#N/D	#N/D	#N/D	#N/D	upstream_gene_variant	MODIFIER	c.-5894T>C	1638
#N/D	#N/D	#N/D	#N/D	#N/D	upstream_gene_variant	MODIFIER	c.-1424T>A	1239
#N/D	#N/D	#N/D	#N/D	#N/D	upstream_gene_variant	MODIFIER	c.-8363T>C	117
#N/D	#N/D	#N/D	#N/D	#N/D	upstream_gene_variant	MODIFIER	n.-2350T>C	2350
#N/D	#N/D	#N/D	#N/D	#N/D	upstream_gene_variant	MODIFIER	c.-7074T>G	309
#N/D	#N/D	#N/D	#N/D	#N/D	upstream_gene_variant	MODIFIER	c.-8909A>G	2770
#N/D	#N/D	#N/D	#N/D	#N/D	upstream_gene_variant	MODIFIER	c.-1285A>G	1256
#N/D	#N/D	#N/D	#N/D	#N/D	upstream_gene_variant	MODIFIER	c.-3951A>G	3935
#N/D	#N/D	#N/D	#N/D	#N/D	upstream_gene_variant	MODIFIER	c.-5437A>G	1448
#N/D	#N/D	#N/D	#N/D	#N/D	upstream_gene_variant	MODIFIER	c.-10502A>G	2914
#N/D	#N/D	#N/D	#N/D	#N/D	upstream_gene_variant	MODIFIER	c.-416T>C	304
#N/D	#N/D	#N/D	#N/D	#N/D	upstream_gene_variant	MODIFIER	c.-24013T>C	1863
#N/D	#N/D	#N/D	#N/D	#N/D	upstream_gene_variant	MODIFIER	n.-1042A>G	1042
#N/D	#N/D	#N/D	#N/D	#N/D	upstream_gene_variant	MODIFIER	c.-2723G>C	2717
#N/D	#N/D	#N/D	#N/D	#N/D	upstream_gene_variant	MODIFIER	c.-1357T>C	1140
#N/D	#N/D	#N/D	#N/D	#N/D	upstream_gene_variant	MODIFIER	c.-1915T>C	1718
#N/D	#N/D	#N/D	#N/D	#N/D	upstream_gene_variant	MODIFIER	c.-4103G>C	3906
#N/D	#N/D	#N/D	#N/D	#N/D	upstream_gene_variant	MODIFIER	c.-5164G>C	4603
#N/D	#N/D	#N/D	#N/D	#N/D	upstream_gene_variant	MODIFIER	c.-14888T>G	2684
#N/D	#N/D	#N/D	#N/D	#N/D	upstream_gene_variant	MODIFIER	c.-5642A>T	3818
#N/D	#N/D	#N/D	#N/D	#N/D	upstream_gene_variant	MODIFIER	c.-17826A>C	299
#N/D	#N/D	#N/D	#N/D	#N/D	upstream_gene_variant	MODIFIER	c.-12978A>G	4213

#N/D	#N/D	#N/D	#N/D	#N/D	upstream_gene_variant	MODIFIER	n.-4058C>G	4058
#N/D	#N/D	#N/D	#N/D	#N/D	upstream_gene_variant	MODIFIER	c.-5897T>C	4937
#N/D	#N/D	#N/D	#N/D	#N/D	upstream_gene_variant	MODIFIER	c.-4031A>C	4031
#N/D	#N/D	#N/D	#N/D	#N/D	upstream_gene_variant	MODIFIER	c.-6972A>C	2716
#N/D	#N/D	#N/D	#N/D	#N/D	upstream_gene_variant	MODIFIER	n.-4772T>C	4772
#N/D	#N/D	#N/D	#N/D	#N/D	upstream_gene_variant	MODIFIER	c.-6537A>C	3155
#N/D	#N/D	#N/D	#N/D	#N/D	upstream_gene_variant	MODIFIER	c.-11778A>G	4941
#N/D	#N/D	#N/D	#N/D	#N/D	upstream_gene_variant	MODIFIER	c.-4975A>G	4797
#N/D	#N/D	#N/D	#N/D	#N/D	upstream_gene_variant	MODIFIER	c.-366A>G	366
#N/D	#N/D	#N/D	#N/D	#N/D	upstream_gene_variant	MODIFIER	c.-1803T>C	1478
#N/D	#N/D	#N/D	#N/D	#N/D	upstream_gene_variant	MODIFIER	c.-19093C>G	4284
#N/D	#N/D	#N/D	#N/D	#N/D	upstream_gene_variant	MODIFIER	c.-2198A>G	2155
#N/D	#N/D	#N/D	#N/D	#N/D	upstream_gene_variant	MODIFIER	c.-3769T>C	536
#N/D	#N/D	#N/D	#N/D	#N/D	upstream_gene_variant	MODIFIER	c.-2590A>G	2286
#N/D	#N/D	#N/D	#N/D	#N/D	upstream_gene_variant	MODIFIER	c.-18270A>G	2993
#N/D	#N/D	#N/D	#N/D	#N/D	upstream_gene_variant	MODIFIER	c.-2312T>C	2312
#N/D	#N/D	#N/D	#N/D	#N/D	upstream_gene_variant	MODIFIER	n.-4368A>C	4368
#N/D	#N/D	#N/D	#N/D	#N/D	upstream_gene_variant	MODIFIER	c.-3750C>G	3037
#N/D	#N/D	#N/D	#N/D	#N/D	upstream_gene_variant	MODIFIER	c.-4244G>C	4129
#N/D	#N/D	#N/D	#N/D	#N/D	upstream_gene_variant	MODIFIER	c.-3731G>C	3709
#N/D	#N/D	#N/D	#N/D	#N/D	upstream_gene_variant	MODIFIER	c.-8623T>C	2787
#N/D	#N/D	#N/D	#N/D	#N/D	upstream_gene_variant	MODIFIER	c.-14601C>G	4854
#N/D	#N/D	#N/D	#N/D	#N/D	upstream_gene_variant	MODIFIER	c.-2949G>C	2793
#N/D	#N/D	#N/D	#N/D	#N/D	upstream_gene_variant	MODIFIER	c.-6291T>C	4024
#N/D	#N/D	#N/D	#N/D	#N/D	upstream_gene_variant	MODIFIER	c.-4842A>G	4744
#N/D	#N/D	#N/D	#N/D	#N/D	upstream_gene_variant	MODIFIER	c.-4306T>C	4115
#N/D	#N/D	#N/D	#N/D	#N/D	upstream_gene_variant	MODIFIER	c.-10203T>C	3600
#N/D	#N/D	#N/D	#N/D	#N/D	upstream_gene_variant	MODIFIER	n.-1081A>G	1081
#N/D	#N/D	#N/D	#N/D	#N/D	upstream_gene_variant	MODIFIER	c.-3601T>C	3516
#N/D	#N/D	#N/D	#N/D	#N/D	upstream_gene_variant	MODIFIER	c.-7974C>G	1242
#N/D	#N/D	#N/D	#N/D	#N/D	upstream_gene_variant	MODIFIER	c.-10790C>G	3884
