# Supplementary material for: Fine scale population structure of Acropora palmata and Acropora cervicornis in the Colombian Caribbean
Source: PeerJ. 2022 Aug 30;10:e13854. doi: 10.7717/peerj.13854 (PMC9438773; doi:10.7717/peerj.13854)
Supplement: Supplemental Information 12 [file peerj-10-13854-s012.pdf]

| ID | Affy probe id | STAGdb probe i | chrom | pos | Ordered Alleles | pca_pvalue_p1 | pca_qvalue | PCA_outlier |
|---|---|---|---|---|---|---|---|---|
| 1282 | AX-212291561 | AP_var_0202 | NW_015441062.1 | 636634 | C // G | 3,04E-11 | 4,76E-08 | outlier |
| 5906 | AX-198060765 | Apop_00661 | NW_015441132.1 | 144090 | T // G | 4,84E-05 | 0,009461947 | outlier |
| 7742 | AX-197963721 | Apop_00870 | NW_015441169.1 | 262004 | A // C | 1,20E-05 | 0,00327725 | outlier |
| 10420 | AX-198061659 | Apop_01220 | NW_015441250.1 | 386766 | A // T | 9,52E-07 | 0,000397426 | outlier |
| 12564 | AX-212313719 | Apop_06222 | NW_015441333.1 | 138182 | A // G | 1,03E-07 | 5,96E-05 | outlier |
| 8453 | AX-212311724 | Apop_04238 | NW_015441186.1 | 571998 | T // C | 4,96E-06 | 0,00165428 | outlier |
| 954 | AX-212312038 | Apop_04552 | NW_015441059.1 | 2074347 | A // C | 2,11E-06 | 0,000776727 | outlier |
| 4854 | AX-197981535 | Apop_02623 | NW_015441112.1 | 689 | T // C | 1,62E-05 | 0,003752732 | outlier |
| 15862 | AX-212314078 | Apop_06594 | NW_015441504.1 | 269728 | A // G | 5,02E-06 | 0,00165428 | outlier |
| 13311 | AX-212294965 | AP_var_3618 | NW_015441366.1 | 217105 | A // G | 2,59E-07 | 0,000115623 | outlier |
| 13314 | AX-212308255 | AP_var_3622 | NW_015441366.1 | 301328 | A // G | 1,05E-07 | 5,96E-05 | outlier |
| 8980 | AX-212313712 | Apop_06215 | NW_015441199.1 | 392473 | C // G | 1,20E-05 | 0,00327725 | outlier |
| 10417 | AX-212307375 | AP_var_2742 | NW_015441250.1 | 380670 | C // G | 1,50E-05 | 0,003622678 | outlier |
| 10308 | AX-198061653 | Apop_01215 | NW_015441248.1 | 82967 | T // C | 1,08E-06 | 0,000421032 | outlier |
| 3296 | AX-212312229 | Apop_04743 | NW_015441086.1 | 844810 | A // T | 2,80E-05 | 0,006041909 | outlier |
| 17471 | AX-212311897 | Apop_04411 | NW_015441667.1 | 212108 | T // C | 1,04E-05 | 0,003086096 | outlier |
| 3232 | AX-212315152 | Apop_07651 | NW_015441084.1 | 481056 | T // G | 4,08E-08 | 2,84E-05 | outlier |
| 5704 | AX-197963323 | Apop_00638 | NW_015441128.1 | 646387 | A // G | 2,42E-05 | 0,005413597 | outlier |
| 17120 | AX-212309531 | AP_var_4901 | NW_015441631.1 | 152298 | T // C | 8,01E-06 | 0,002507675 | outlier |
| 14068 | AX-212298114 | Apop_03914 | NW_015441395.1 | 352472 | A // G | 1,02E-11 | 2,12E-08 | outlier |
| 4652 | AX-212292466 | AP_var_1116 | NW_015441106.1 | 262062 | T // C | 1,67E-08 | 1,31E-05 | outlier |
| 14072 | AX-212308431 | AP_var_3798 | NW_015441395.1 | 378982 | A // G | 1,02E-11 | 2,12E-08 | outlier |
| 13312 | AX-212308253 | AP_var_3620 | NW_015441366.1 | 257443 | T // C | 1,88E-09 | 1,68E-06 | outlier |
| 1166 | AX-212312423 | Apop_04938 | NW_015441061.1 | 1159352 | A // G | 1,56E-07 | 8,12E-05 | outlier |
| 12378 | AX-212307964 | AP_var_3331 | NW_015441322.1 | 108834 | A // G | 4,77E-05 | 0,009461947 | outlier |
| 14071 | AX-212295144 | AP_var_3797 | NW_015441395.1 | 365079 | A // C | 1,87E-09 | 1,68E-06 | outlier |
| 1316 | AX-197940800 | Afix_02022 | NW_015441062.1 | 918163 | T // C | 1,89E-07 | 9,10E-05 | outlier |
| 12831 | AX-212308167 | AP_var_3534 | NW_015441352.1 | 44330 | A // C | 3,39E-05 | 0,007084476 | outlier |
| 14074 | AX-212295147 | AP_var_3800 | NW_015441395.1 | 398611 | C // G | 8,04E-10 | 1,01E-06 | outlier |
| 18263 | AX-212309906 | AP_var_5276 | NW_015441820.1 | 18619 | A // T | 3,14E-13 | 1,97E-09 | outlier |
| 10309 | AX-212307349 | AP_var_2716 | NW_015441248.1 | 86832 | T // C | 1,33E-05 | 0,003340638 | outlier |
| 10311 | AX-212307350 | AP_var_2717 | NW_015441248.1 | 101541 | T // C | 1,33E-05 | 0,003340638 | outlier |
| 3131 | AX-212301852 | Apop_07639 | NW_015441082.1 | 1097586 | A // C | 0,240837903 | 0,761889603 | n.s. |
| 4839 | AX-212302125 | Apop_07912 | NW_015441110.1 | 343884 | A // G | 0,241289664 | 0,761889603 | n.s. |
| 10277 | AX-212300925 | Apop_06728 | NW_015441244.1 | 144431 | A // G | 0,580531191 | 0,864718735 | n.s. |
| 12377 | AX-212307963 | AP_var_3330 | NW_015441322.1 | 99775 | A // G | 0,000704461 | 0,045947476 | n.s. |
| 17627 | AX-212313350 | Apop_05853 | NW_015441701.1 | 103365 | T // C | 0,00011925 | 0,019145622 | n.s. |
| 19525 | AX-212304622 | Apop_10392 | NW_015442478.1 | 31541 | T // C | 0,534014113 | 0,854986819 | n.s. |

| OUTFLANK_fst | OUTFLANK_qva | OUTFLANK outli | CDS annotation | Protein accessic | mRNA accessior | Adig genome v2 |
|---|---|---|---|---|---|---|
| 0,418444647 | 0,000189071 | outlier | A disintegrin and metalloproteinase with throm | XP_015759874.: | XM_015904388 | aug_v2a.16603 |
| 0,166261269 | 0,338652177 | n.s. | #N/D | #N/D | #N/D | #N/D |
| 0,187854083 | 0,23753233 | n.s. | #N/D | #N/D | #N/D | #N/D |
| 0,221408267 | NA | NA | #N/D | #N/D | #N/D | #N/D |
| 0,261221929 | NA | NA | #N/D | #N/D | #N/D | #N/D |
| 0,284081935 | 0,021720461 | outlier | #N/D | #N/D | #N/D | #N/D |
| 0,170036477 | 0,338652177 | n.s. | #N/D | #N/D | #N/D | #N/D |
| 0,229780484 | 0,070163694 | n.s. | #N/D | #N/D | #N/D | #N/D |
| 0,249473851 | 0,056886971 | n.s. | #N/D | #N/D | #N/D | #N/D |
| 0,415342969 | 0,000302536 | outlier | #N/D | #N/D | #N/D | #N/D |
| 0,330551407 | 0,004407907 | outlier | SAFB-like transcription modulator | XP_015769099.: | XM_015913613 | aug_v2a.10066 |
| 0,155852297 | 0,338652177 | n.s. | ras-associated and pleckstrin homology domains | XP_015760915.: | XM_015905429 | aug_v2a.15306 |
| 0,221408267 | NA | NA | uncharacterized LOC107342869 | XP_015763869.: | XM_015908383 | aug_v2a.00275 |
| 0,182723698 | 0,255443476 | n.s. | tumor necrosis factor receptor superfamily mem | XP_015763745.: | XM_015908259 | aug_v2a.14076 |
| 0,190702638 | 0,111366918 | n.s. | fibropellin-1-like%2C transcript variant X3 | XP_015751614.: | XM_015896128 | aug_v2a.14876 |
| 0,287797709 | 0,021720461 | outlier | uncharacterized LOC107355283 | XR_001567007.: | #N/D | #N/D |
| 0,213176962 | 0,131242883 | n.s. | cyclic AMP-responsive element-binding protein 3 | XP_015751395.: | XM_015895909 | aug_v2a.20705 |
| 0,127370291 | 0,467084743 | n.s. | zinc finger protein 429-like | XP_015755900.: | XM_015900414 | aug_v2a.05150 |
| 0,220769492 | 0,080098952 | n.s. | uncharacterized LOC107354539 | XP_015776505.: | XM_015921019 | aug_v2a.11224 |
| 0,417475142 | 0,000189071 | outlier | CREB-binding protein-like%2C transcript variant | XP_015770280.: | XM_015914794 | aug_v2a.03049 |
| 0,212239652 | 0,139005298 | n.s. | putative ATP-dependent RNA helicase TDRD9 | XP_015753856.: | XM_015898370 | aug_v2a.13797 |
| 0,417475142 | 0,000189071 | outlier | non-histone protein 10-like isoform X1 | XP_015770286.: | XM_015914800 | aug_v2a.03047 |
| 0,443152724 | 0,000189071 | outlier | beta-1,3-galactosyltransferase 5-like | XP_015769103.: | XM_015913617 | aug_v2a.00094 |
| 0,298870492 | 0,013481792 | outlier | #N/D | #N/D | #N/D | #N/D |
| 0,346599869 | 0,002674883 | outlier | #N/D | #N/D | #N/D | #N/D |
| 0,417475142 | 0,000189071 | outlier | #N/D | #N/D | #N/D | #N/D |
| 0,032442041 | 0,98588884 | n.s. | #N/D | #N/D | #N/D | #N/D |
| 0,093753381 | NA | NA | #N/D | #N/D | #N/D | #N/D |
| 0,348348777 | NA | NA | #N/D | #N/D | #N/D | #N/D |
| 0,552571601 | NA | NA | #N/D | #N/D | #N/D | #N/D |
| 0,184537579 | 0,255443476 | n.s. | #N/D | #N/D | #N/D | #N/D |
| 0,184537579 | 0,255443476 | n.s. | #N/D | #N/D | #N/D | #N/D |
| 0,26063029 | 0,021720461 | outlier | #N/D | #N/D | #N/D | #N/D |
| 0,222879293 | 0,044410229 | outlier | #N/D | #N/D | #N/D | #N/D |
| 0,228443796 | 0,006891154 | outlier | #N/D | #N/D | #N/D | #N/D |
| 0,361077589 | 0,001744204 | outlier | #N/D | #N/D | #N/D | #N/D |
| 0,395850323 | 0,000460863 | outlier | #N/D | #N/D | #N/D | #N/D |
| 0,228224708 | 0,023050969 | outlier | #N/D | #N/D | #N/D | #N/D |

| GO Terms | KEGG | SNPeff prediction | Putative_impac | DNA change | AA change | cDNA_position , |
|---|---|---|---|---|---|---|
| GO:0004222 GC | #N/D | 5_prime_UTR_variant | MODIFIER | c.-169G>C | | |
| #N/D | #N/D | downstream_gene_variant | MODIFIER | c.*8386T>G | | |
| #N/D | #N/D | downstream_gene_variant | MODIFIER | n.*3504T>G | | |
| #N/D | #N/D | downstream_gene_variant | MODIFIER | c.*2819A>T | | |
| #N/D | #N/D | downstream_gene_variant | MODIFIER | c.*3633A>G | | |
| #N/D | #N/D | downstream_gene_variant | MODIFIER | c.*8627T>C | | |
| #N/D | #N/D | intergenic_region | MODIFIER | n.2074347A>C | | |
| #N/D | #N/D | intergenic_region | MODIFIER | n.689T>C | | |
| #N/D | #N/D | intergenic_region | MODIFIER | n.269728A>G | | |
| #N/D | #N/D | intergenic_region | MODIFIER | n.217105A>G | | |
| GO:0016021; G( | #N/D | intron_variant | MODIFIER | c.160-1313A>G | | |
| GO:0007165 | K23488 | intron_variant | MODIFIER | c.979-205G>C | | |
| #N/D | #N/D | synonymous_variant | LOW | c.162G>C | p.Ala54Ala | 622/3189 |
| GO:0005576 | K20825 | 3_prime_UTR_variant | MODIFIER | c.*537T>C | | |
| GO:0005102 GC | #N/D | intron_variant | MODIFIER | c.*2-8463T>A | | |
| #N/D | #N/D | intron_variant | MODIFIER | n.336+1030A>G | | |
| GO:0003677 GC | K09048 | intron_variant | MODIFIER | c.519+33T>G | | |
| GO:0003676 GC | K09214 | synonymous_variant | LOW | c.306T>C | p.Asp102Asp | 405/5002 |
| GO:0001527 GC | #N/D | intron_variant | MODIFIER | c.1028-350A>G | | |
| GO:0000123 GC | K04498 | intron_variant | MODIFIER | c.2661+24A>G | | |
| GO:0000166 GC | K18408 | missense_variant&splice_region_variant | MODERATE | c.907A>G | p.Arg303Gly | 1014/1994 |
| GO:0000404 GC | K11670 | synonymous_variant | LOW | c.888T>C | p.Phe296Phe | 921/1099 |
| GO:0000139 GC | K02175 | synonymous_variant | LOW | c.963A>G | p.Met321Met | 1182/1464 |
| #N/D | #N/D | upstream_gene_variant | MODIFIER | c.-23295A>G | | |
| #N/D | #N/D | upstream_gene_variant | MODIFIER | n.-4098T>C | | |
| #N/D | #N/D | upstream_gene_variant | MODIFIER | c.-9636A>C | | |
| #N/D | #N/D | upstream_gene_variant | MODIFIER | c.-2356A>G | | |
| #N/D | #N/D | upstream_gene_variant | MODIFIER | c.-2145T>G | | |
| #N/D | #N/D | upstream_gene_variant | MODIFIER | c.-7761C>G | | |
| #N/D | #N/D | upstream_gene_variant | MODIFIER | c.-24608A>T | | |
| #N/D | #N/D | upstream_gene_variant | MODIFIER | c.-8268T>C | | |
| #N/D | #N/D | intergenic_region | MODIFIER | n.101541T>C | | |
| #N/D | #N/D | upstream_gene_variant | MODIFIER | c.-10737A>C | | |
| #N/D | #N/D | downstream_gene_variant | MODIFIER | n.*702A>G | | |
| #N/D | #N/D | upstream_gene_variant | MODIFIER | c.-1625T>C | | |
| #N/D | #N/D | upstream_gene_variant | MODIFIER | c.-1062T>C | | |
| #N/D | #N/D | upstream_gene_variant | MODIFIER | n.-3055T>C | | |
| #N/D | #N/D | upstream_gene_variant | MODIFIER | c.-9379T>C | | |

| CDS_position / ( | Protein_positio | Distance to feature: |
|---|---|---|
| | | 2146 |
| | | 3992 |
| | | 3504 |
| | | 950 |
| | | 1974 |
| | | 3022 |
| | | |
| | | |
| | | |
| | | |
| | | |
| | | |
| 162/2118 | 54/705 | |
| | | 537 |
| | | |
| | | |
| | | |
| 306/912 | 102/303 | |
| | | |
| | | |
| 907/1887 | 303/628 | |
| 888/918 | 296/305 | |
| 963/996 | 321/331 | |
| | | 4892 |
| | | 4098 |
| | | 4279 |
| | | 2332 |
| | | 1920 |
| | | 4535 |
| | | 2053 |
| | | |
| | | |
| | | 4394 |
| | | 702 |
| | | 1625 |
| | | 880 |
| | | 3055 |
| | | 1220 |
